# *Operando* Electrochemical Fluorescence Microscopy Highlights Local Buffering and Mass Transport Effects in Polymer-Promoted Electrocatalysis

**DOI:** 10.1021/jacs.6c11297

**Published:** 2026-10-06

**Authors:** Steffen Hardt, Katherine J. Levey, Mark Aarts, Bonho Koo, Lars J. C. Jeuken, Marc T. M. Koper

**Affiliations:** 4496 Leiden University, Faculty of Science, Leiden Institute of Chemistry, Einsteinweg 55, Leiden 2333 CC, The Netherlands

## Abstract

Many electrocatalytic reactions proceed via proton-coupled electron transfer (PCET) mechanisms, which can be promoted by protic polymer coatings. However, polymers modulate the local reaction environment in many ways. To deconvolute the effect of local pH in protic polymer coatings from other contributions, we developed a method for *operando* determination of in-polymer pH values using electrochemical fluorescence microscopy. Polyethylenimine (PEI) was equipped with a fluorescent pH probe, coated on polycrystalline gold, and electrochemical hydrogen evolution was applied as a pH-sensitive model reaction. Temporal changes in the average pH values across the entire thickness of the polymer film were quantified by widefield microscopy, while the spatial distribution of pH profiles within the films was determined by confocal laser scanning microscopy (CLSM). Complementing the experimental results with finite-element modeling identified two polymer-induced effects: (i) local differences in the mass transport of the proton donors and (ii) a pronounced local buffering by the highly concentrated ammonium groups inside the polymer coating. The latter was corroborated by linear sweep voltammetry (LSV): while the buffering PEI coating decreased the onset potential of hydrogen evolution, the control polymers poly­(vinyl alcohol) and PiperION, which cannot be deprotonated in the aqueous pH window, did not promote electrocatalysis. These findings highlight the *operando* pH modulation by protic polymer coatings, and understanding this phenomenon will be crucial for rational design of polymer coatings for PCET reactions.

## Graphical abstract

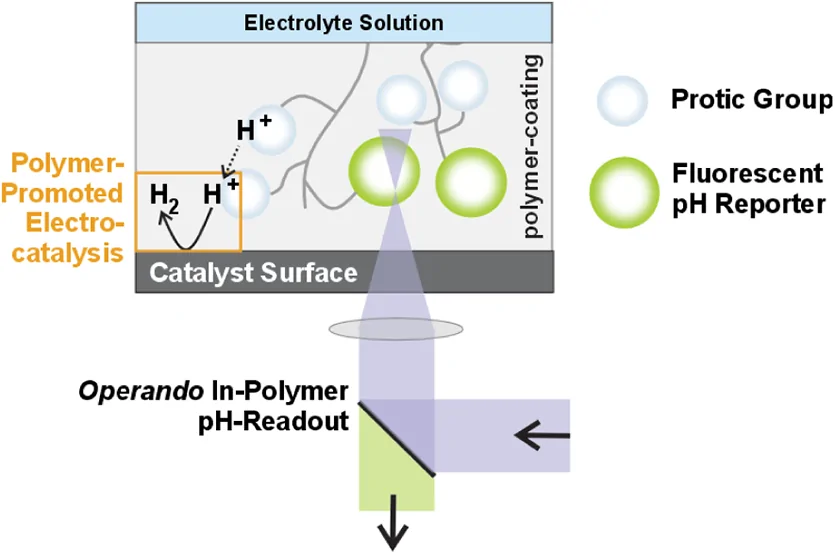

## Introduction

Environmentally relevant electrochemical reactions like hydrogen evolution, CO_2_-reduction, and N_2_-reduction proceed via proton-coupled electron transfer (PCET) processes.^1–3^ Therefore, an efficient and selective electrocatalytic process depends on both favorable electron transfer kinetics and well-balanced proton transfer, which is challenging to achieve in heterogeneous electrocatalysis.^4^ Nature has solved this issue by the evolution of oxidoreductase enzymes, such as hydrogenase, formate reductase, or nitrogenase, wherein proton delivery to the active site is ensured by dedicated proton channels.^5^ These enable enzymes to operate as an electrocatalyst at high turnover rates^6^ and have inspired the design of artificial catalysts equipped with proton-conducting groups.^7–9^ Recently, this concept has been transferred to heterogeneous electrocatalysis by polymer coatings that mimic the complex reaction environment of an enzyme in a very simplified manner. Using this strategy, significant promotion of electrochemical CO_2_-reduction, nitrite reduction, and hydrogen evolution was realized.^10–17^ The proposed explanations are manifold: modulation of the local water structure,^16^ changes in local hydrophobicity/hydrophilicity,^12^ and the facilitation of gas release.^14^ Since PCET reactions are inherently pH-dependent, there is an urgent need for precise knowledge on the in-polymer pH to draw reliable conclusions on the origin of beneficial polymer effects. Information on the local pH has previously been obtained by several methods at unmodified electrodes,^18^ but examples of in-polymer pH detection are scarce and typically lack spatial resolution.^19–21^

Polymers are highly adaptable synthetic platforms and have, among others, already successfully been equipped with morphology switches,^22^ redox mediators,^23^ (chiral) catalysts,^24^ and pharmaceutically active functionalities.^25^ This adaptability can be exploited to gain information about the in-polymer pH. Of particular interest for local and time-resolved pH determination is their modification with pH-responsive dyes (ref^26^ and references therein). Dye-functionalized membranes enabled *in situ* UV–vis spectroscopy for qualitative visualization of pH changes.^19,20^ However, spatial information has only been obtained by an ensemble of different polymer coatings deposited in an elaborate layer-by-layer process.^21^ This excludes the detection of possible in-polymer inhomogeneities in individual films. This limitation can be overcome by the use of fluorescent pH probes combined with appropriate microscopy techniques. In electrochemistry, fluorescent pH detection has been successfully applied for the activity mapping of surfaces^27,28^ and local hotspots of corrosion.^29^ *Operando* pH profiles were visualized by suitable dyes during HER on the macroscale,^30–32^ while investigations using confocal laser scanning microscopy (CLSM) allowed zooming into proton diffusion and transport processes on the microscale.^33–37^ The variety of targets that can be investigated is huge, spanning from model metal surfaces^33^ over microelectrodes,^34,36^ electrolyzers,^31^ and fuel cells^32^ to electroenzymatic^37,38^ and microbial electrodes.^39^

We herein developed a strategy to deconvolute the effects of the local pH from other contributions induced by polymer coatings. As a model system, we use the HER at polycrystalline gold coated with thin layers of polyethylenimine (PEI), which exhibits a considerable increase of the HER activity as compared to bare gold electrodes. By modifying the PEI with 6-carboxyfluorescein, we map the spatiotemporal pH changes *operando* by fluorescence microscopy. While widefield fluorescence microscopy quantifies the temporal development of the in-polymer pH, CLSM can visualize the spatial pH profiles inside the polymer film. The spatiotemporal pH changes are simulated by a finite-element model that couples the rates of electrochemical reaction to mass transport by diffusion and to the acid–base buffer chemistry of the polymer film and the electrolyte. The model validated the method and elucidated the role of the polymer in modulating the pH by a local buffering effect (Figure 1 A). We consider the presented method a valuable and versatile tool, offering great opportunities in heterogeneous, homogeneous, and bioelectrocatalysis beyond the presented HER model reaction (e.g., CO_2_RR,^40^ nitro-group reduction,^41^ ammoximation^42^).

**Figure 1:**
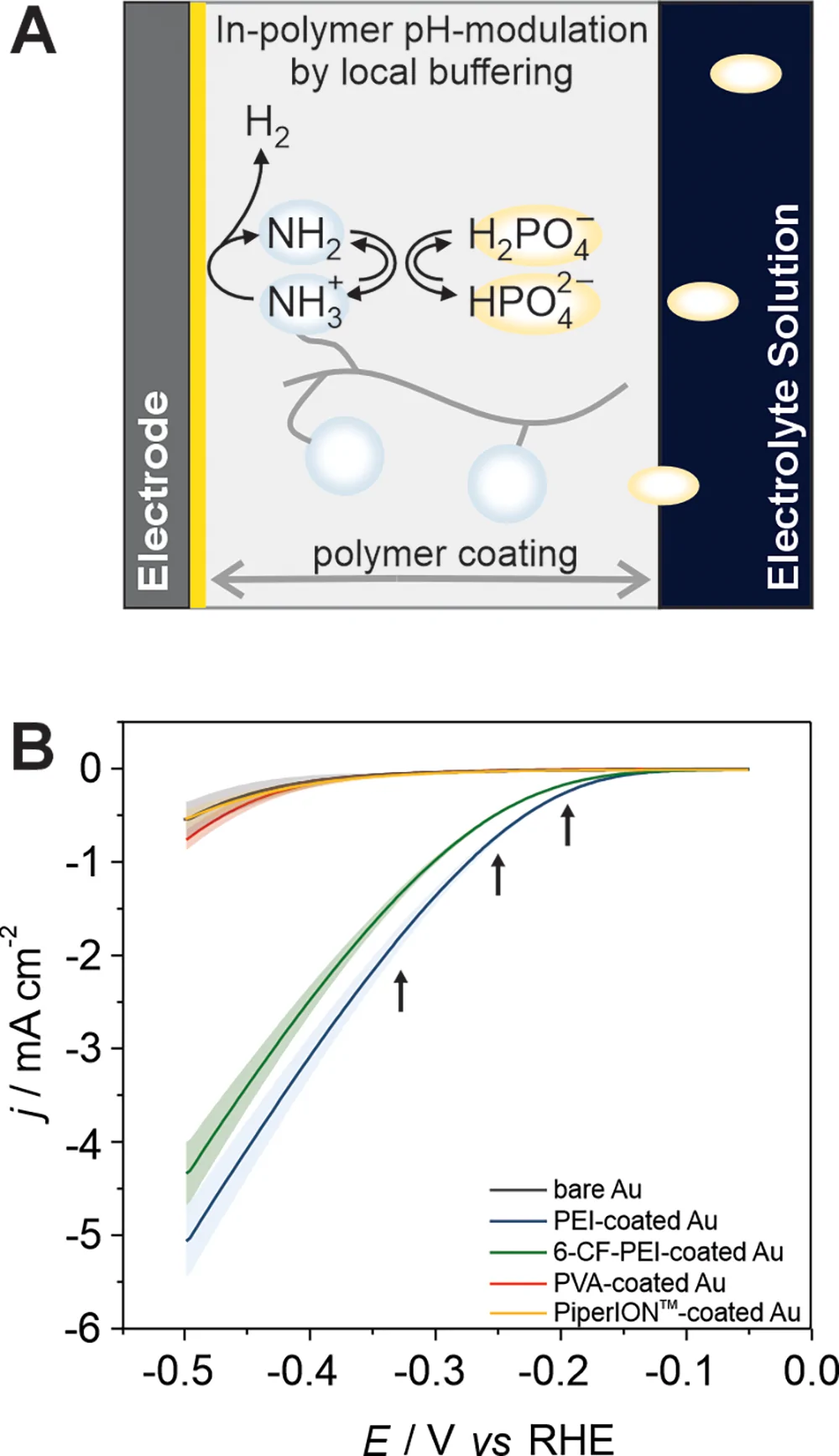
HER acceleration by PEI coatings. A. Schematic of polymer-induced local buffering accelerating the HER with the polymer’s amine groups depicted as gray spheres and the phosphate species in the electrolyte as orange spheres. B. LSVs (ν = 10 mV s^–1^, *A* = 0.19 cm^2^) of Au-RDEs without coating (black), with a PEI coating (blue), a 6-CF-PEI coating (green), a PVA coating (red), or a PiperION coating (yellow) in potassium phosphate buffer (0.1 M, pH 6.2) under 2,000 rpm rotation. Currents are normalized to the ECSA determined from the AuO reduction peak after the experiment (Figure S5). Current densities are given as average values (solid lines) ± standard deviation (shaded area) of three independent experiments. The arrows indicate the potentials observed during *operando* pH detection in Figure 2.

## Results and Discussion

Hydrogen evolution on polycrystalline gold rotating disc electrodes (Au-RDEs) is a well-understood PCET reaction, with previous investigations having targeted the role of the local pH.^43–45^ The acceleration of the HER by amine-containing polymer coatings has previously been shown at various metal surfaces.^10,13,14^ Therefore, we deposited films of the amine-containing polymer PEI on polycrystalline gold electrodes by drop-casting an aqueous polymer solution in the presence of a succinimidyl cross-linker (BS-3, for details see Scheme S1 and Experimental Section). The obtained polymer films were very stable, under both operation (no observable loss upon an hour of chronoamperometry, Figure S1 D) and storage (no observable loss after 8 days in KH_2_PO_4_ solution, Figure S1 A–C). The polymer-coated electrodes were characterized by linear sweep voltammetry (LSV) at a disk rotation rate of ω = 2,000 rpm in potassium phosphate buffer (0. 1 M) at pH 6.2 and exhibited a 10-fold current increase at −0.5 V vs RHE as compared to uncoated Au-RDEs (blue vs black trace in Figure 1 B). This promotional effect was independent of the degree of cross-linking (Figure S2) and changed only marginally when the bulk pH was decreased to 4.5 (Figure S3). Online gas chromatography revealed a high faradaic efficiency of 91.8 ± 4.7% (*n* = 8) at a low overpotential (*E*_app_ = −0.3 V vs RHE), confirming the absence of undesired side reactions at the polymer-coated electrode (Figure S4). A control coating with the polymer poly­(vinyl alcohol) (PVA), which cannot be deprotonated within the aqueous pH window, exhibited the same LSV response as the bare gold electrode (Figure 1 B, red trace). This allowed us to exclude effects of the polyvinyl backbone. To evaluate the role of charges within a polymer coating, gold electrodes were covered with PiperION, a positively charged polymer without buffer capacity. This polymer showed no promotion of the HER (Figure 1 B, yellow trace). From this observation, we conclude that PEI, although positively charged at slightly acidic pH, does not behave like a common anion exchange membrane (AEM). Instead, it promotes the HER by local buffering by its three (de)­protonation equilibria with p*K*_a1_ = 4.5–5 (primary amines), p*K*_a2_ = 6.7–8.5 (secondary amines), and p*K*_a3_ = 10.5–11.6 (tertiary amines) (Figure 1 A).^46,47^

To measure these polymer-induced local pH effects, PEI was equipped with the fluorescent pH reporter 6-carboxyfluorescein (6-CF). When excited at λ_ex_ = 490 nm, this compound is highly fluorescent in the deprotonated state, but weakly fluorescent in the protonated state (Figure 2 A, right panel), has a suitable p*K*_a_ of 6.4 within the buffering region of the electrolyte and the polymer, and allows for simple covalent attachment to the polymer backbone (Scheme S2, for a detailed synthesis procedure and full spectral characterization, see Supplementary Section 2).^48,49^ After covalent attachment of 6-CF to PEI, its absorption/emission spectra (Figure S7) and pH sensitivity (Figure S8) were maintained. Addition of the fluorophore to the polymer backbone only led to minor changes in the LSV response (green vs blue traces in Figure 1 B), indicating that the influence of the fluorophore on the local pH and the reaction kinetics is acceptably small. The small current loss observed cannot be solely attributed to primary amines blocked by fluorophore binding as discussed in Supplementary section 2.

**Figure 2:**
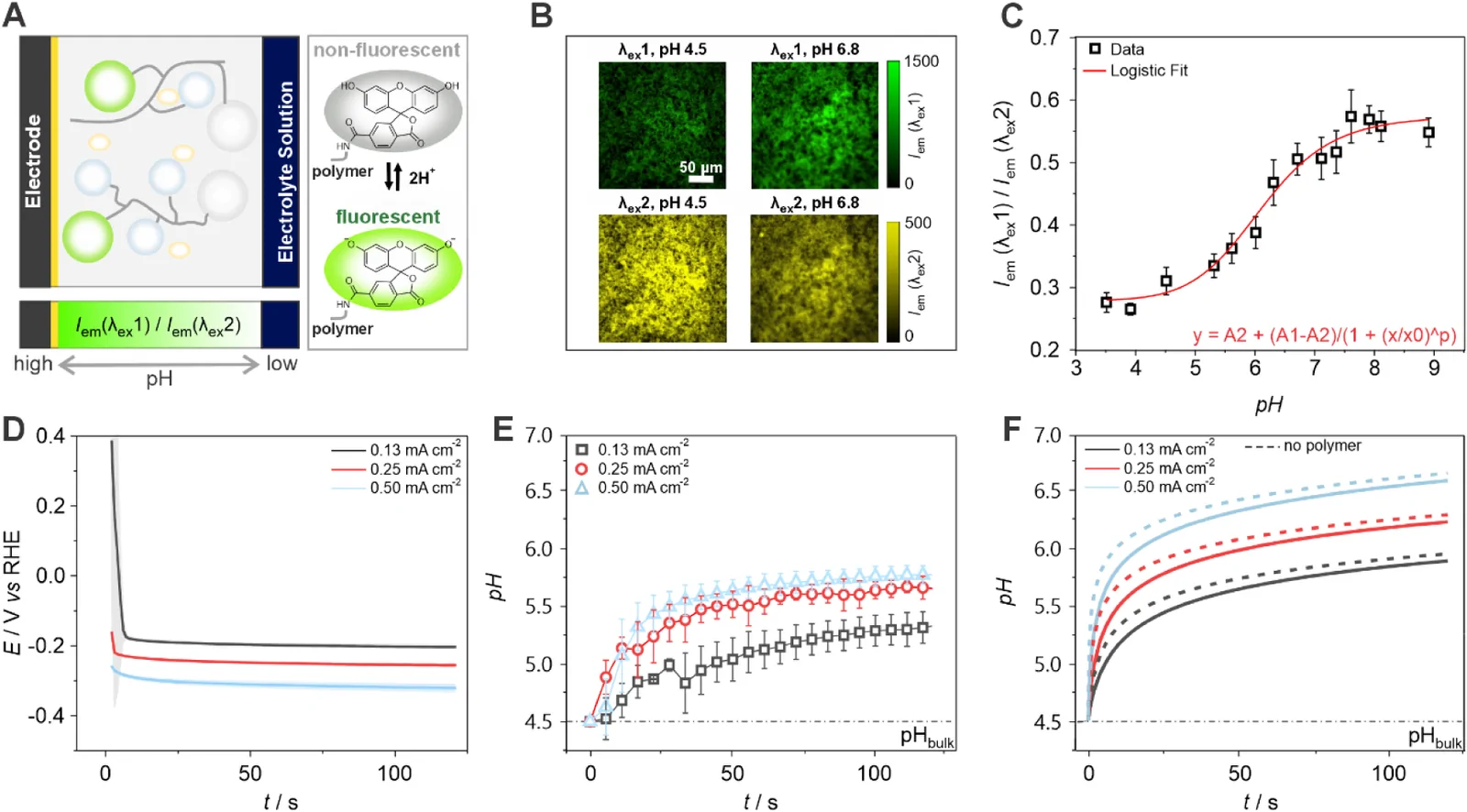
*Operando* pH readout. A. Conceptional Figure of the 6-CF-PEI film (gray, shaded area) containing amine groups (blue spheres) and 6-CF molecules (green spheres) for pH readout. Expected pH gradients are measured using the pH-dependent emission ratio of the fluorophore (gradient bar). The right side of panel A shows the (de)­protonation equilibrium of the fluorophore with the fluorescent state being depicted in green and the nonfluorescent state in gray. B. Exemplary pictures recorded at two different excitation wavelength ranges (λ_ex_1 = 467–498 nm, λ_em_1 = 513–556 nm) (λ_ex_2= 400 – 440 nm, λ_em_2 > 450 nm) at two different pH values as indicated. C. pH-Calibration obtained from potassium phosphate buffer solutions (0.1 M) at different pH values. Intensity ratios (black squares) were extracted from the mean intensities of images such as those in panel B and fitted by a logistic function (red curve, R^2^ = 0.93, p*K*_a_ = 6.1, equation as depicted in the panel). The data are presented as average values ± standard deviation of six individual data points from three polymer films. The nonaveraged data can be found in Figure S12 A. D. Chronopotentiometric transients (CPs) (A = 0.39 cm^2^, j_app_ as indicated) in KH_2_PO_4_ solution (0.1 M, pH 4.5). The data are presented as average values (solid line) vs standard deviation (shaded area) from three individual measurements at two different films. The high reproducibility is seen from the small standard deviations, making the shaded areas hardly visible. E. Temporal development of the pH values inside the 6-CF-PEI film corresponding to the CPs in panel D. The pH values are averaged over the whole film thickness. The data are presented as average (open symbols) ± standard deviation from three individual measurements on two polymer films. The nonaveraged data can be found in Figure S12 B. An extension to higher overpotentials and currents is given in Figure S12 C and D. F. Finite-element model of the temporal pH changes applying the same current densities as in panel E. The dotted lines represent simulations of bare gold electrodes. For the solid lines, the polymer was considered as an immobilized buffer species as outlined in Supplementary Section 5. The corresponding simulated CPs, with far more negative potentials needed for the uncoated case, agree with the experiments and can be found in Figure S12 E.

Temporal changes in the in-polymer pH were followed by widefield fluorescence microscopy. pH calibration of the polymer film’s fluorescence was accomplished by extracting the mean intensities of images (representative examples shown in Figure 2 B) at different pH values using an excitation-ratiometric approach (for further details, see Experimental Section).^50,51^ Control experiments confirmed that, while the absolute emission intensities were affected by photobleaching, the ratiometric pH readout was not, thus ensuring a robust pH determination even under prolonged light exposure (Figure S9). The obtained intensity ratios were fitted by a logistic function (Figure 2 C). The inflection point of the fit (pH = 6.14 ± 0.06) only marginally differed from the p*K*_a_ value of the unbound fluorophore (p*K*_a_ = 6.4), indicating that the pH response of 6-CF-PEI remained mostly unaffected after being deposited as a cross-linked film. In the next step, the fit function served as a calibration curve for the subsequent *operando* experiments.

The *operando* experiments were realized by using a tailor-made electrochemical cell compatible with an inverted fluorescence microscope (Figure S10 A and Figure S11).^52^ The working electrode, which simultaneously served as the bottom layer of the electrochemical cell, was a glass coverslip (*Menzel*, diameter: 25 mm, thickness: 0.13–0.16 mm) functionalized with a 30 nm layer of gold (Figure S10 C). The low thickness of the Au layer ensured the light transparency required for microscopy, while still exhibiting the typical electrochemical response of a polycrystalline Au surface (Figure S10 B). Atomic force microscopy (AFM) confirmed a flat gold surface, which avoids optical modulation effects that can occur at micro- or nanostructured gold (Figure S16).^53^ An initial pH (pH_ini_ = 4.5) was chosen as it marks the lower edge of the detectable pH region, and a pH increase is expected under operation. Hydrogen was evolved for 2 min by chronopotentiometry at three different current densities (Figure 2 D). These were chosen such that the corresponding overpotentials, as indicated by the arrows in Figure 1 B, were low enough for the HER only originating from the polymer-covered sections without a contribution of the bare gold electrode. As expected, an increase in the local pH values was observed for all three current densities, and the pH changes inside the film were more pronounced at higher currents (i.e., at a higher consumption of protons) (Figure 2 E). A pronounced pH change at the beginning was followed by a plateau after ∼1 min. This was rationalized by the buildup of a steady pH profile with a continuous flux of protons, either by hopping or by mass transport of the proton donors. Accordingly, the local pH increase disappears over time upon switching off the current, a process that was accelerated by forced convection (Figure S13). The fact that intensity changes are mass-transport-dependent confirmed that pH is responsible for the fluorescence intensity changes.

Having an experimental readout method of in-polymer pH values, we applied finite-element modeling in *COMSOL Multiphysics* (for details, see Supplementary Section 5) to elucidate how the polymer modulates the local pH. To this end, we first simulated the temporal development of the pH in the absence of the polymer film, establishing a control case in which only mass transport and electrolyte buffering were considered. This resulted in higher local pH values and a steeper increase in pH over time than what was observed experimentally (Figure 2 F, dashed lines). We hypothesize that the buffering capacity of the polymer, represented in Figure 1 A, plays a crucial role in the development of the local pH. Hence, in the next step, the buffer capacity of the polymer film was integrated into the model by adding the acid–base reactions associated with the protonatable amine groups within the film (eqs. S19−S21). Considering the amount of drop-coated polymer (3.5 μg) and the size of the polymer film (thickness: 1.6 μm by CLSM, Figure S15, diameter ∼4.5 mm), an amine-group concentration of 3.8 M was estimated. The polymer consisted of a mixture of primary, secondary, and tertiary amines in a 1:2:1 ratio having p*K*_a_ values of approximately 5, 7 and 11, respectively.^46,47^ The polymer-bound amines are immobile and, hence, their diffusion coefficients were fixed to zero in the model. This description of the polymer coating reproduced the temporal change in pH during chronopotentiometry more accurately, confirming the contribution of the polymer layer to local pH (compare panels E and F in Figure 2). From the model, we identified a rapid deprotonation rate of the ammonium groups being beneficial for effective buffering (Figure S19 C and D). Presumably, this will become more pronounced at high-current application, where proton supply must compete with higher turnover rates. This is corroborated by the finding that the model suggests a region of increased pH within a few nanometers from the electrode, wherein the buffer species are out of equilibrium (Figure S21 A).

Furthermore, we found that the modeled CP transients were severely influenced by varying the p*K*_a_ of the secondary amines (Figure S19 E). These differences corresponded to a decreased in-film pH under *operando* conditions, when the polymer’s p*K*_a_ was lower (Figure S19 F). Tailoring the polymer’s p*K*_a_ to a target reaction beyond HER may, therefore, be a powerful tool to steer reactivity and selectivity.

Despite the inclusion of polymer buffering, the model and experiment were found to deviate by up to 1 pH unit. This is because (i) we restricted the parameters of the model to values that were either reported in the literature or estimated from separate experiments on bare gold electrodes to avoid overfitting of the results (Supplementary Section 5) and (ii) the partial coverage of the gold surface with the polymer film may induce deviations in the local current density.

Comparing the large overpotential decrease by the polymer coating in Figure 1 B with the moderate pH effect in Figure 2 F suggests that the promotion of the HER is not a pure local pH effect but accompanied by yet unexplored phenomena, possibly including local ion concentrations^54^ and coordination of intermediates by the amine groups.^13^

The average pH values throughout the whole thickness determined above deliver valuable information about the role of polymer-induced buffering. Still, a more detailed understanding of potentially limiting processes in electrocatalysis depends on spatial information on the pH distribution throughout the polymer film. Therefore, we extended the method to *operando* electrochemical confocal laser scanning microscopy (EC-CLSM) (for details, see Supporting Information Section 6). Chronopotentiometric transients identical to those in widefield microscopy were recorded (Figure S23 A and B). Z-scans were collected after 2 min of current flow, as a steady state is expected after this duration given the stable pH values in Figure 2 E. Depending on the polymer film and the individual positions inside a polymer film (Figure S23 F, I, L), two different cases were observed: polymer positions that exhibited a homogeneous pH distribution throughout the whole thickness of the polymer film (Figure 3 A) and positions exhibiting a pH profile with a high pH close to the electrode surface and a lower pH at the polymer/electrolyte interface (Figure 3 C). Both cases show the expected increase in the in-film pH with applied currents and show similar pH values at the film solution interface agreeing with those obtained by widefield microscopy in Figure 2. However, the significant spatial differences in pH are not covered by widefield microscopy, highlighting the additional insights obtained from CLSM. These are very valuable because severe local pH gradients increase the overpotential requirements of electrochemical processes, yet a correlation between the local pH and the chronopotentiometric transients would require a systematic rastering of the film. In this context, the increasing maturity of microscopy automation holds great potential.^55^ The possibility of temporal focus drift affecting the pH readout was excluded by the use of an automated focus control system (*Nikon PFS*). This was further confirmed by recording z-scans at OCP before and after the measurement, showing exactly the same film position in the z-direction (Figure S23 D, E, G, H, J, K and Experimental Section). Furthermore, pH fluctuations caused by convection are unlikely as discussed in Supplementary Section 6.

**Figure 3:**
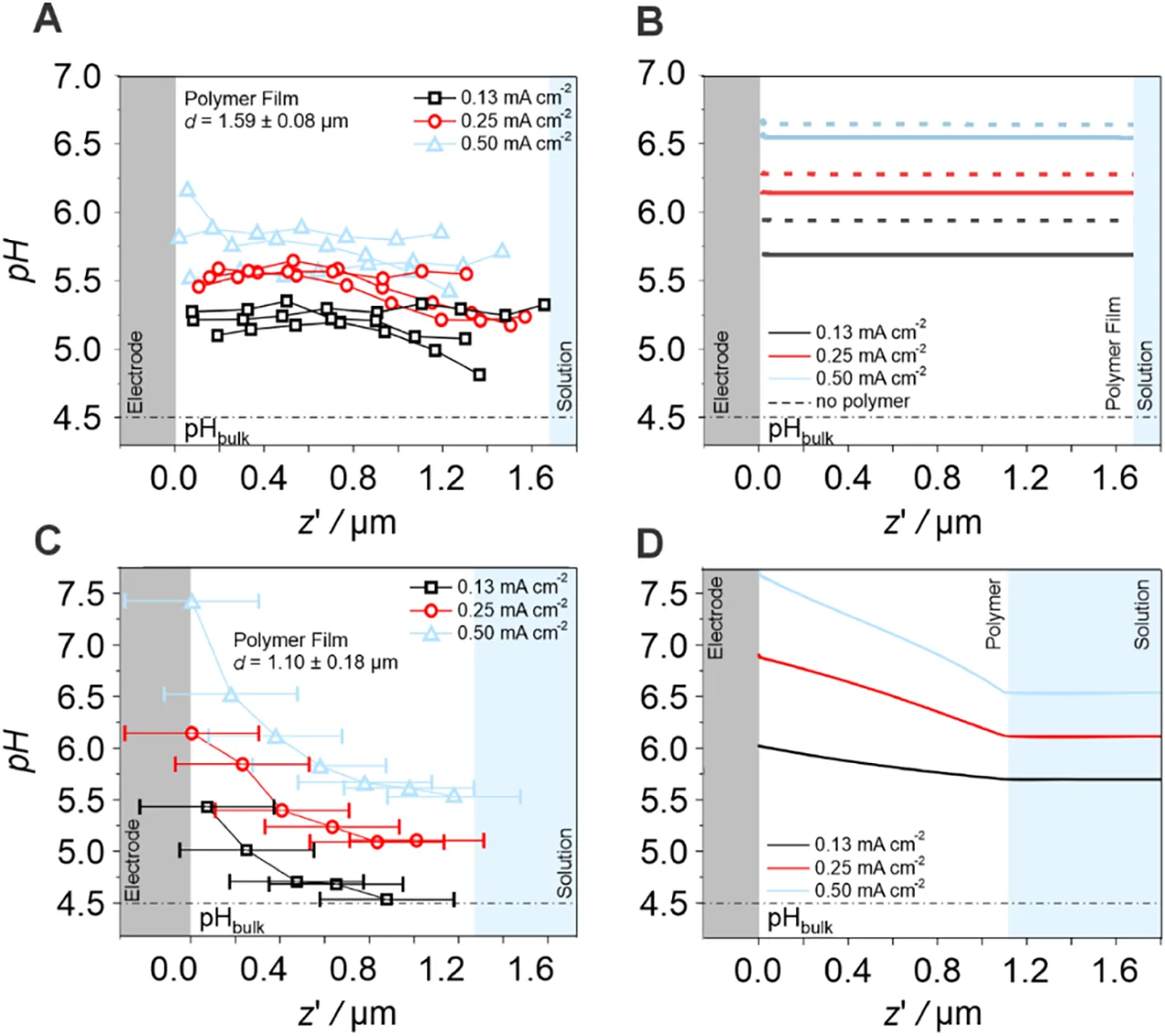
Results of EC-CLSM. A. pH Distribution collected after the chronopotentiometries (*t* = 2 min, *j*_app_ as indicated in the legend) given in Figure S23 showing either flat pH distributions (A) or concentration profiles (C). The dimensions of the polymer film are represented by the white background. The electrode is depicted in gray and the electrolyte in light blue. The bulk pH is given as the black dotted line. The average film thickness *d* is given in the respective panels. Panel A shows three individual film positions at each current density. Measurement insecurities induced by the z-resolution limit of the microscope (±0.3 μm) are represented by the x-error bars in panel C. The corresponding calibration procedure is presented in Figure S22 and Supplementary Section 6. Simulated pH distributions at a PEI-covered electrodes assume either unrestricted (B) or restricted mass transport through the polymer layer (D). Simulations of the polymer-free electrode are given as dashed lines in panel B. An extended view of the pH profile to the millimeter scale exhibits a complete pH relaxation to the bulk pH remote from the electrode surface (Figure S21 B and C).

The fact that, depending on the measured position, two different pH distributions can be observed, is most likely explained by local differences in the polymer film. These may be caused by local differences in the degree of cross-linking. A locally increased cross-linking results in a higher density of the film potentially restricting water and phosphate accessibility. A higher local density is corroborated by the finding that film positions that exhibit pH profiles are reproducibly thinner (*d* = 1.10 ± 0.018 μm) compared to those exhibiting a homogeneous pH value (*d* = 1.59 ± 0.08 μm). Furthermore, cross-linking converts the polymer’s amine groups into amides (Scheme S1) that do not participate in local buffering. Consequently, the amount of accessible proton donors is significantly decreased, resulting in a severe local pH increase.

We used the finite-element model to further test this hypothesis. The positions showing homogeneous pH distributions are, despite an offset of the absolute pH values, represented by the model when mass transport through the polymer layer is unrestricted (compare Figure 3 A and B). The lower local pH values observed in the experiments were, again, addressed by including the local buffering effect of the polymer coating (compare dashed and dotted lines in Figure 3 B). Restricting the mass transport of proton donors by decreasing the diffusion coefficients of the phosphate species (*D*_phos_) by 3 orders of magnitude (compare panels B and D in Figure 3) resulted in pronounced pH gradients, which qualitatively agree with the experimental findings. Complementing the thickness determination by AFM exhibited an even lower film thickness of ∼0.5 μm (Figure S16) possibly due to differences in film formation, when preparing the AFM sample (Supplementary Section 4). Implementing the lower thickness (and the higher amine concentration resulting from a lower film volume) lead to only insignificant changes in the simulation results and does, thus, not affect our conclusion on local buffering and locally restricted mass transport (Figure S20).

An additional contributor to differences in the local pH profiles may be inhomogeneities in the distribution of primary, secondary, and tertiary amines. While primary and secondary amines have a sufficiently low p*K*_a_ to contribute to buffering, this is not the case for tertiary amines.^46,47^ Therefore, polymer regions with an increased fraction of tertiary amines may contribute to higher local pH values. Restricted proton transport may also be caused by the formation of a rigid hydrogen bond network between the ammonium groups and the phosphates, similar to those reported in polyelectrolyte mixtures^11^ or in natural and artificial polypeptides.^56^ An in-depth investigation of the role of cross-linking amount, coating procedure, and drying conditions will be very valuable to clarify this hypothesis but is beyond the scope of this investigation.

## Conclusion and Outlook

In this work, we developed a method combining organic chemistry, optics, and electrochemistry for *operando* readout of in-polymer pH values. Fluorophore-decorated polymer coatings combined with electrochemical widefield and confocal laser scanning microscopy enabled us to investigate the spatiotemporal development of in-polymer pH during hydrogen evolution on gold as model reaction. Finite-element modeling identified the polymer’s amino/ammonium groups as highly concentrated local buffer. Experimental concentration profiles highlighted the complexity of polymer-induced effects on the local pH with individual polymer regions being exposed to markedly different local pH values. The spatiotemporal determination of the local pH demonstrated here addresses a very general and timely problem in electrochemistry, wherein local environment effects and catalyst reactivity cannot be determined independently. Being able to measure the *operando* in-polymer pH values bears the potential of deconvoluting local pH effects from other polymer-induced phenomena like modulation of the local water structure,^16^ changes in the local hydrophobicity/hydrophilicity^12^ and the facilitation of gas release.^14^

This will contribute to a more comprehensive understanding of polymer/electrocatalyst interactions, which is highly relevant considering the omnipresence of polymers in electrochemistry as membranes,^57^ immobilization matrices,^9,23^ binders in batteries,^58^ or capping agents for nanoparticles.^59,60^ Especially, the combination of confocal microscopy with porous model electrodes bears great potential to investigate the effects of local pH gradients.^61^ Additionally, in-polymer pH readout has broad relevance beyond surface electrocatalysis because polymer-induced effects on the reaction environments can, to a certain degree, be considered as a simplified model of outer-sphere effects commonly observed for biocatalysts.^5^ Our readout strategy can, therefore, contribute to the understanding and development of bioinspired electrocatalytic systems. Against this background, the versatility of fluorescent probes that can quantify a variety of analytes, e.g., reactive oxygen species,^62^ radicals,^63^ ions,^64^ water,^65^ and specific organic molecules^66^ provides exciting future opportunities to investigate reaction environments far beyond the commonly addressed local pH.

## Experimental Section

### Materials

Buffer and electrolyte salts were purchased from Sigma-Aldrich or Thermofisher Scientific in 99.99% purity and stored in an desiccator before use. Phosphoric acid (85 wt % in water, 99.99% purity) for the preparation of solutions of pH < 4.5 was purchased from Sigma-Aldrich. 6-Carboxyfluorescein (6-CF) was purchased from Merck Novabiochem in ≥ 95% purity. Polyethylene imine (PEI, M_W_ = 750 000 g/mol, *M*_n_ ≈ 60 000 g/mol) was purchased from Sigma-Aldrich as a 50 wt % aqueous solution. 1-Ethyl-3-[3-(dimethylamino)­propyl]­carbodiimide (EDC, 99%) was purchased from ABCR. *N*-Hydroxysuccinimide (98%), D_2_O (99%), as well as H_2_SO_4_ (p.a. 95–97%) were purchased from Sigma-Aldrich. The cross-linker bis­(sulfosuccinimidyl)­suberate (BS3, purity >93%) was purchased from Thermofisher Scientific Pierce. DCM, acetone, and *i*PrOH of p.a. purity (99.8–99.9%) were purchased from Honeywell Riedel-de Haën. Methanol for PVA workup was purchased from Macron Fine Chemicals (99.9%). PiperION dispersion (5%wt, 74820000) was purchased from Versorgen (Delaware). All aqueous solutions were prepared from Milli-Q water with a resistivity of 18.2 MΩ·cm. All chemicals were used without further purification. An exception was poly­(vinyl alcohol) (PVA), which was purchased from Sigma-Aldrich under the trading name Mowiol 10–98 (M = 61 000 g/mol, 98–98.8% purity) and purified before use in the following manner: Poly­(vinyl alcohol) (1 g) was dissolved in water (19 mL) and precipitated from a 1:1 solution of acetone and methanol (40 mL). The PVA was dried under vacuum at 60 °C overnight.

### Electrode Preparation

Polycrystalline Au rotating disc electrodes (PINE Instruments, diameter 5 mm) were used as working electrodes. They were polished three times with diamond suspensions of decreasing particle size (3 μm, 1 μm, 0.25 μm) for 2 min each, followed by sonication in Milli-Q water for 5 min. Subsequently, the electrodes were exposed to an electropolishing procedure (100 CV cycles, *E*_upper vertex_ = 1.75 V vs RHE, *E*_lower vertex_ = 0.05 V vs RHE, ν = 1 V/s) in H_2_SO_4_ (0.1 M). After electropolishing, a CV at 50 mV/s was recorded (10 cycles) in the same potential window. The polished working electrodes were then either transferred into a measuring cell containing potassium phosphate buffer (0.1 M) to conduct the control experiments with uncoated Au, or they were functionalized with a polymer coating.

The coating was formed by drop-casting a mixture of PEI or 6-CF-PEI (5 μL, 0.7 mg/mL) and BS3 cross-linker (5 μL, 0.7 mg/mL) onto the electrode surface (Figure S10 D). For the control experiments in Figure S2 the cross-linker concentration was reduced to 0.07 and 0.007 mg/mL for panels B and C, respectively. The films were allowed to dry for 72 h at 4 °C. After drying, the films were vigorously rinsed with Milli-Q water to remove eventual unbound species. PVA coatings were prepared by the same protocol without a cross-linker. PiperION films (3.5 μg) were coated from a 5%wt solution in an organic solvent (undisclosed by the supplier) and dried for 2 h at RT. Prior to use, the films were immersed in a phosphate buffer solution (0.1 M, pH 6.2) for 12 h.

### Electrochemical Experiments

Electrochemical experiments were conducted at a Biologic VSP 300 potentiostat controlled by EC Lab V11.60 software in an in-house-made glass measuring cell cleaned in advance with KMnO_4_ solution and diluted piranha acid. An annealed Au wire (*Matec*, 99.99%) was used as counter and a commercial reversible hydrogen electrode (*Gaskatel Hydroflex*) as the reference electrode. The electrolyte solutions were intensively bubbled with Ar for 10 min. A single-frequency impedance measurement at OCP at a frequency of *f* = 100 kHz was performed, and the solution resistance was calculated as R_Ω_ ≈ Re Z (ZIR method). The following electrochemical experiments were corrected for 85% of the ohmic drop in the EC Lab V11.60 software. CVs (ν = 10 mV/s, ω = 0, 3 cycles) were recorded in potassium phosphate buffer (0.1 M, pH 6.2 or pH 4.5) in a potential range of 0 to −1 V vs RHE. Subsequently, rotation was started (ω = 2,000 rpm) and CVs (ν = 10 mV/s, 3 cycles) in two potential ranges (0 to −0.5 V vs RHE and 0 to −1 V vs RHE) were recorded. The negative-going scans of the second cycle were plotted in Figures 1 B and S19 B, respectively. After the experiments, the electrodes were transferred to H_2_SO_4_ (0.1 M), and CVs were recorded (*E*_upper vertex_ = 1.75 V vs RHE, *E*_lower vertex_ = 0.05 V vs RHE, ν = 50 mV/s) to determine the electrochemically active surface area (ECSA) by integration of the AuO reduction signal (*E*_p_ = 1.15 V vs RHE) of the first cycle (Figure S5 A). The quantity of transferred charge was divided by 386 μC/cm^2^ and the obtained ECSAs were applied to normalize the currents.^67^ For both bare Au electrodes and polymer-coated electrodes, a fraction of the ECSA became blocked during the experiment (Figure S5 B and C), which is why it was decided to normalize the currents to the ECSAs determined after the full procedure.

### Gas Chromatography

An online gas chromatograph (GC 2014, Shimadzu) with an FID (Shincarbon column) and with a TCD detector (RTX-1 column) was utilized. The chromatograph was connected to the cathode compartment of an in-house-made H-cell (V ≈ 5 mL per compartment) under a flow of N_2_ (10 mL/min) controlled by a mass flow controller (*SLA5850, Brooks Instrument*). The counter electrode (dimensionally stable anode, *Magneto*) was separated by an anion exchange membrane (*ForBlue Selemion*, *AGEC*). The collection efficiency was determined by conducting electrochemical hydrogen evolution at a bare gold electrode. A PEI-coated gold electrode was connected as working electrode, and intact coating was ensured by CV. A chronoamperometry (Figure S4, black line) was conducted for 85 min. After 5 min, a first sample of the gas stream was injected, followed by further injections every 15 min. Afterward, the same electrode was used for a chronopotentiometric (Figure S4, red line) experiment and the GC procedure was repeated. A CV after both experiments revealed an identical response as before, suggesting that the film being unaffected by extended operation (Figure S4 C).

### Preparation of Electrochemistry-Coupled Fluorescence Microscopy

To prepare the microscopy-compatible electrodes, a previously reported procedure was adapted:^52^ a silicon wafer (*University Wafer, Inc., USA*) was cleaned by sonication in DCM followed by sonication in *i*PrOH for 5 min each. After an additional 30 min of ozone cleaning (Bioforce Nanoscience, USA) a 30 nm layer of Au was deposited onto the wafer by an e-beam evaporator (Plassys BESTEK MEB600SL). Gold was evaporated at 0.1 nm/s, and during deposition, the wafer was rotating at 10 rpm to establish a consistent thickness over the whole area. Glass coverslips (Menzel, diameter 25 mm, thickness 0.13–0.16 mm) were glued onto the Au-covered wafer using a nonfluorescent Epo tek 310 2FL glue (Epoxy Technologies, USA). The glue was hardened by placing the wafer onto a heat plate at 80 °C for 3 h. After cooling to RT, the coverslips were stripped off the wafer, transferring the Au layer to the glass. The smoothness of the Au layers was confirmed by AFM (black areas in Figure S16 A and C). Polymer films were deposited onto the electrode surface by the drop-coating procedure described above and were dried at 4 °C under a moist atmosphere. The electrode was vigorously rinsed with water to remove unbound species. The cleaned, polymer-functionalized electrode was positioned at the bottom of the measuring cell, which was tightly sealed with four screws. An Au wire was flattened at the end using a hydraulic press and was clamped between the electrode and the body of the measuring cell for electrical connection. Details on the measuring cell can be found in Supplementary Section 3. Polymer films were allowed to rest in potassium phosphate buffer solution (0.1 M, pH 6.2, 2 mL) overnight to guarantee full wetting. The horizontal arrangement of the working electrode avoided convectional effects being a common error source in optical local pH measurements.^32^

### Atomic Force Microscopy

AFM measurements in buffer solutions were conducted with a JPK NanoWizard 4 in QI mode, using *SNL-10* probes (Bruker, nominal radius 2 nm).

### *Operando* Widefield (EPI) Fluorescence Microscopy

The measuring cell was positioned onto an inverted fluorescence microscope (Nikon Eclipse Ti) equipped with a Nikon Plan objective (40x magnification, *NA* = 0.67), a Hamamatsu camera (*C13440 Orca Flash 4.0*) and a halogen lamp (*EXFO X-Cite Series 120*). The microscope was controlled by ImageJ add in MMStudio Version 2.0.0 beta 3. Every film was calibrated individually by the following procedure: The measuring cell was filled with H_3_PO_4_/KH_2_PO_4_ solution (0.1 M, pH 3.4, 2 mL) and images were recorded using two different Nikon filter cubes, FITC (λ_ex_ = 467–498 nm, λ_em_ = 513–556 nm) and BV2A (λ_ex_ = 400–440 nm, λ_em_ > 450 nm), at an exposure duration of 2 s and a relative light intensity of 25%. The pH was stepwise increased with increments of ca. 0.3 by exchanging the phosphate solution in the measuring cell. Upon every buffer exchange, the measuring cell was rinsed with the new buffer repeatedly (8 × 2 mL). Equilibration to the new pH value was rapid in agreement with the literature.^26^ To account for possible heterogeneities of the film, a set of images was recorded at three different positions per pH. The mean intensities of the images were extracted using Fiji ImageJ freeware. The obtained values were corrected by subtracting the background intensity recorded at a polymer-free spot of the Au electrode. The background intensity was found to be pH independent making one background measurement sufficient for the full measurement procedure of an electrode (Figure S14). The ratio of the background-corrected mean intensities was plotted against pH, leading to sigmoidal calibration curves fitted to a logistic function (Figure 2). After full calibration, the pH was set to the starting value of 4.5. Images of the film exhibited the same emission intensity ratio as at the beginning of the calibration, showing that the pH-induced intensity changes are reversible. Imaging a selected position under continuous light exposure for 2 min in the absence of an applied potential/current ensured the robustness of the ratiometric pH readout strategy against intensity decreases due to photobleaching (Figure S9).

After complete calibration, an Au-wire counter electrode and a Gaskatel Hydroflex RHE reference electrode were added. The electrodes were connected to an Autolab PGStat101 potentiostat controlled by Nova 2.1.7 software. pH changes were induced by chronoamperometry or chronopotentiometry with applied potentials or currents, as given in the respective figure legends. Images were recorded by the automized timelapse function of the software, and image acquisition was started simultaneously with the electrochemical experiment. The temporal resolution defined by the exposure duration and the time demand for automated change of the filter cube was 5.5 s between every set of images. Intensity ratios were determined by the aforementioned procedure and were translated into pH values using the logistic fit function of the respective calibration curve as detailed in the Supporting Information.

### Confocal Fluorescence Laser Scanning Microscopy

The same microscope was applied for EC-CLSM. Instead of a halogen lamp, a Coherent Obis Galaxy laser source (λ_ex_ = 514 nm, *I*_ex_ = 1%) connected to a Confocal.nl unit was applied. A Nikon oil objective (magnification 100x, *NA* = 1.87) was used. As control experiments revealed that even at the lowest laser intensities (*P* = 13 μW), bleaching could not be avoided (compare black and gray intensity distributions in Figure S23 D, E, G, H, J, K), a calibration procedure was developed that accounts for the bleaching as outlined in Supplementary Section 6. The lasers were controlled by Coherent Connection 3 software and the microscope by NIS-Elements AR 5.11.02 software. *Z*-stacks were recorded at a set of different positions using increments of 0.2 μm in KH_2_PO_4_ (0.1 M, pH 4.5), and the positions were stored. The mean intensity of each slice of the stack was extracted and corrected for the background intensity at a polymer-free position of the electrode. The background signal appeared to be independent of both pH and z-position (Figure S22 A and B). The intensities were plotted as a function of *z* and fitted to a Gaussian (Figure S22 D). The full width half maximum (fwhm) was determined and defined as the region inside the film, with the lower x-value at half height representing the electrode/film interface and the higher *x*-value of half height the film/solution interface, respectively (Figure S15).^68^ The pH was increased using the same buffer exchange protocol as for the widefield fluorescence measurements, and *z*-stacks at the predefined positions were recorded. Mean intensities were extracted, background corrected and normalized as detailed in Supplementary Section 6. This resulted in a flat distribution of intensity ratio throughout the whole thickness of the film (Figure S22 F) with small deviations being present only at high pH (Figure S22 E). Thus, the interdependence of normalized intensity and pH can be described by a 2D logistic fit function for every position within the *z*-stack (Figure S22 G).

For the *operando* measurements, the same electrochemical equipment as that for the widefield fluorescence experiments was utilized. The focus point was moved to a fresh unbleached position. Then, a *z*-stack was recorded at an open-circuit potential (OCP) (= before a current was flowing). Subsequently, a CP was recorded for 200 s with *E*_app_ as specified in the respective figure legends. After 120 s, *z*-stacks were recorded at the same position. Comparison of these z-stacks shows either a good alignment of the intensity distribution in the presence (red trace) and absence (black trace) of an applied potential (Figure S23 D, G, J) or a shift of the intensity maximum toward the electrode surface (Figure S23 E, H, K). Normalization of the intensities by their initial values as detailed in Supplementary section 6 followed by recalculation to pH gave the pH distributions obtained in Figure 3 A and C. The readout method is critically dependent on the absence of focus drifts and pH-induced swelling/shrinking because both would render the normalization invalid. This was ensured by extensive control experiments. The absence of swelling was confirmed by CLSM (Figure S22 C) and AFM (Figure S24) at different pH values. The absence of focus drifts was assured by using a *Nikon Perfect Focus System* (PFS), which readjusts the sample position based on the glass reflection induced by an auxiliary laser. Control experiments recording intensity profiles at OCP at the measured film position 20 min after the experiment confirmed the absence of a focus drift (compare the black and gray traces in Figure S23).

### Finite-Element Model

A one-dimensional finite-element model was developed that couples the buffer chemistry of the film and electrolyte with the mass transport of species in both the domains (Figure S18). The model assumes that diffusion is the sole mode of mass transport, migration, and convection are neglected. The protonatable head groups are assumed to be uniformly distributed throughout the film, and steric effects associated with the finite size of species are not considered. The HER is implemented as a boundary equation at the electrode surface, considering both proton and water reduction pathways, with the reaction rates described by Butler–Volmer kinetics. The coupled time-dependent equations were solved using *COMSOL Multiphysics* (*version 6.2*) with the Transport of Dilute Species interface. A full description of the model is included in Supplementary Section 5, along with details of the mesh, boundary conditions, and parameter choice.

## Supplementary Material











## Data Availability

Data sets for this article are available in the Zenodo repository under the Digital Object Identifier (DOI): 10.5281/zenodo.22773588

## Notes

Details on polymer film formation, thickness and stability, polymer synthesis and characterization, *operando* widefield and confocal laser scanning fluorescence microscopy (including the experimental setup, data treatment, and calibration procedure), and modeling details (PDF)
*COMSOL* modeling report (PDF)
*Excel* sheet for recalculation of emission intensities to pH (XLSX)
*MatLab* script for data readout and evaluation (ZIP)
^1^H NMR data (ZIP)
