# Supplementary material for: Operando Electrochemical Fluorescence Microscopy Highlights Local Buffering and Mass Transport Effects in Polymer-Promoted Electrocatalysis
Source: J Am Chem Soc. 2026 Oct 6;148(41):44300–9. doi: 10.1021/jacs.6c11297 (PMC13693391; doi:10.1021/jacs.6c11297)
Supplement: Supplementary file 1 [file ja6c11297_si_001.pdf]

Supporting Information

Operando Electrochemical Fluorescence Microscopy Highlights Local Buffering and Mass Transport Effects in Polymer-Promoted Electrocatalysis

Steffen Hardt*, Katherine J. Levey, Mark Aarts, Bonho Koo, Lars J.C. Jeuken, Marc T.M. Koper*

Leiden University, Faculty of Science, Leiden Institute of Chemistry, Einsteinweg 55, 2333 CC Leiden, The Netherlands.
**Correspondence:* s.hardt@umail.leidenuniv.nl
m.koper@lic.leidenuniv.nl

Supplementary Section 1 – Polymer Film Formation and Stability, Electrochemical Control Experiments 2

Supplementary Section 2 – Synthesis and Characterization of the Fluorescent Polymer 6-Carboxyfluorescein polyethylene imine (6-CF-PEI) 5

Supplementary Section 3 – Fluorescence Microscopy 10

Supplementary Section 4 – Film Thickness Determination 14

Supplementary Section 5 – Computational Details and Parameter Extraction 17

Supplementary Section 6 – Evaluation of Confocal Laser Scanning Microscopy 29

References 37

Supplementary Section 1 – Polymer Film Formation and Stability, Electrochemical Control Experiments

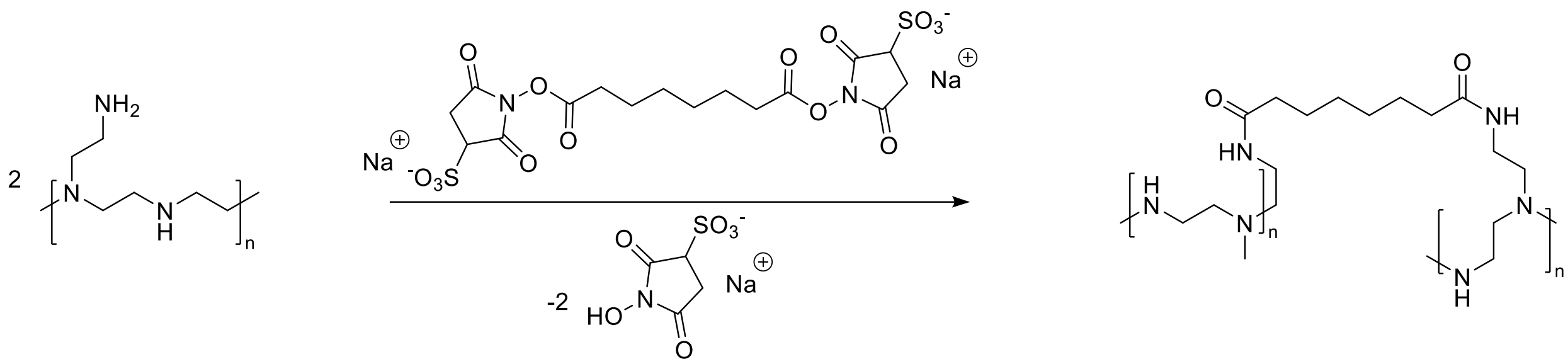

Scheme S1. Crosslinking reaction. Note that the reaction is depicted only at the primary amines for the sake of clarity. It will simultaneously take place at the secondary amines.

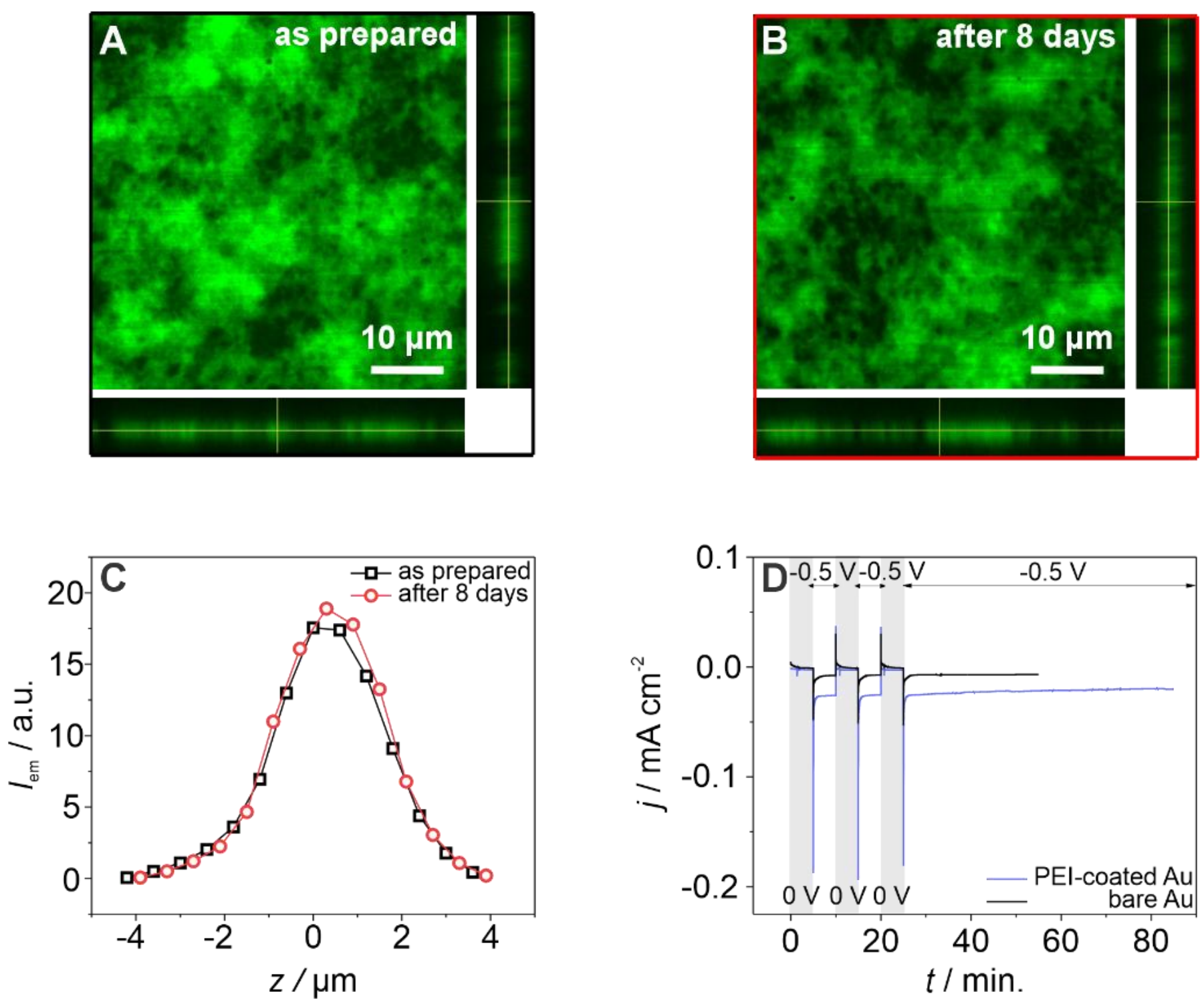

Figure S1: Stability of the crosslinked PEI-coating. CLSM-images and orthogonal views of a 6-CF-PEI film showing a representative region of interest directly after drying (**A**) and after 8 days of storing in KH_2PO_4 solution (0.1 M, pH 4.5) (**B**). **C**. Intensity profiles of the z-scans belonging to the images in **A** and **B**. No significant change in the mean intensities is observed after 8 days of storing in solution (red) as compared to the as prepared film (black). **D.** Stability of PEI-films under operation conditions. Chronoamperometry (CA, E_{app} *vs*. RHE as indicated) of a PEI-coated (blue) and bare gold electrode (black, $A = 0.28$ cm^2) under stationary conditions (ω = 0 rpm). The promotional effect of the polymer coating remained for more than 80 minutes. Intermittent operation did not decrease the performance. The film remained optically visible after the experiment.

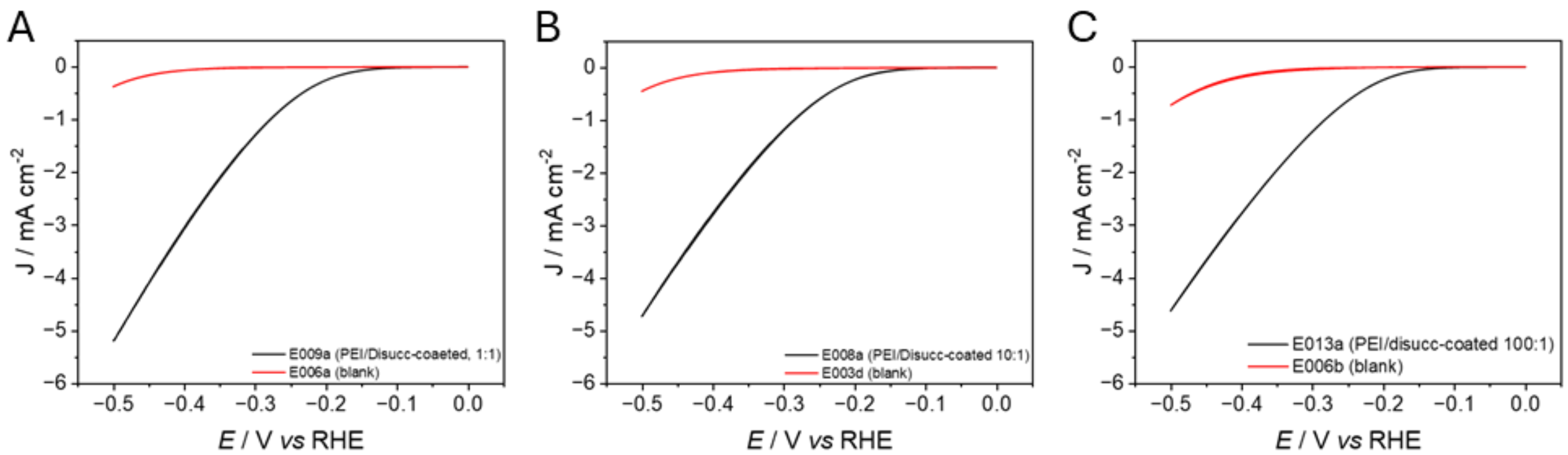

Figure S2. Influence of crosslinker. CVs (ν = 10 mV s^{-1}, ω = 2,000 rpm, A = 0.19 cm^2) of Au-RDEs coated with crosslinked PEI (black traces) compared to the respective bare Au-electrodes (red traces) at three different mass ratios of polymer and crosslinker: 1:1 (**A**), 10:1 (**B**) and 100:1 (**C**).

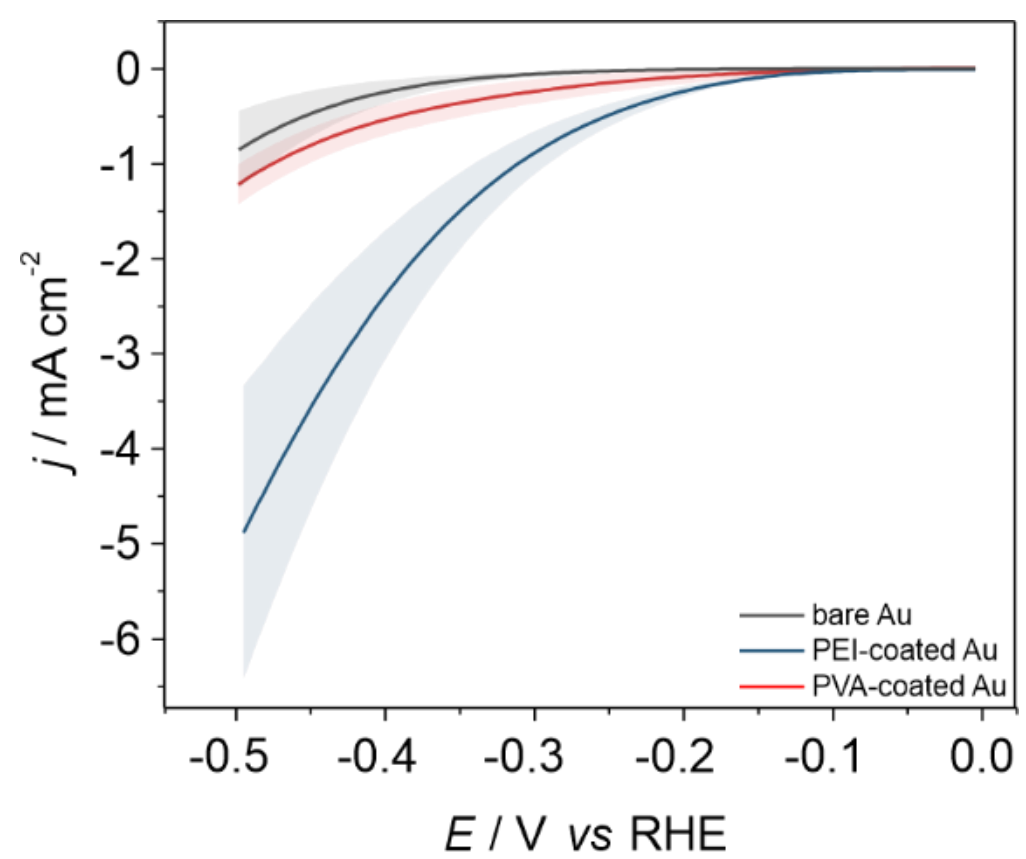

Figure S3: Effect of decreased pH. LSVs (ν = 10 mV s^{-1}, A = 0.19 cm^2) of Au-RDEs without coating (black), with a PEI-coating (blue), or with a PVA-coating (red) in KH_2PO_4-solution (0.1 M, pH 4.5) under 2,000 rpm rotation. The results are presented as average values (solid lines) ± standard deviation (shaded areas) from three individual electrodes.

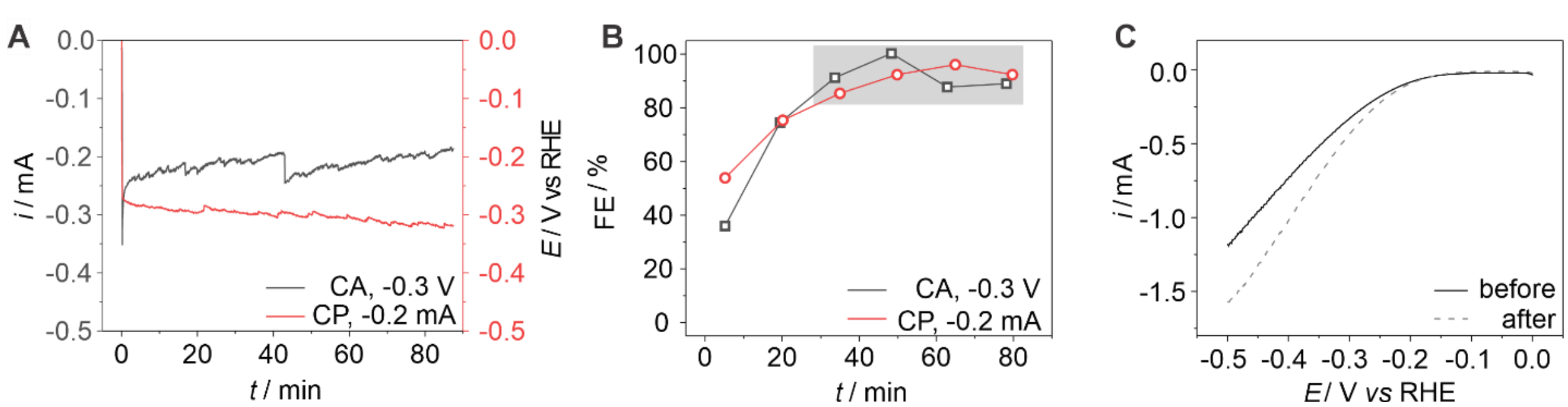

Figure S4: Reaction product of the PEI-coated electrodes. A. Chronoamperometric (black) and chronopotentiometric transients (red) of a PEI-coated gold electrode (A = 0.28 cm^2) under a constant flow of N_2 (10 mL / min) with E_{app} and i_{app} as indicated. After ~ 5, 20, 35, 50, 65, and 80 minutes, GC samples were injected (**B**) from the gas stream with the black and the red trace belonging to the CA and CP experiment, respectively. After an initial equilibration period of 35 minutes faradaic efficiencies of 91.8 ± 4.7 % were reached (average value from the 8 measuring points highlighted by the gray box). The corresponding chromatograms can be found in the supplementary data. **C**. LSVs of the PEI-coated Au-electrode (ν = 10 mV s^{-1}) before and after the GC-experiment. Gas bubbles were removed by shaking the electrode prior to recording of the LSV. The performance of the electrode did not decrease throughout 160 minutes of electrolysis, rendering polymer degradation unlikely. Fluctuations in the CA-current and gradual overpotential increase in the CP were assigned to bubble formation. The slight increase in current in the LSV after electrolysis may be related to a temporal increase of electrolyte content and/or accessible electrode surface area.

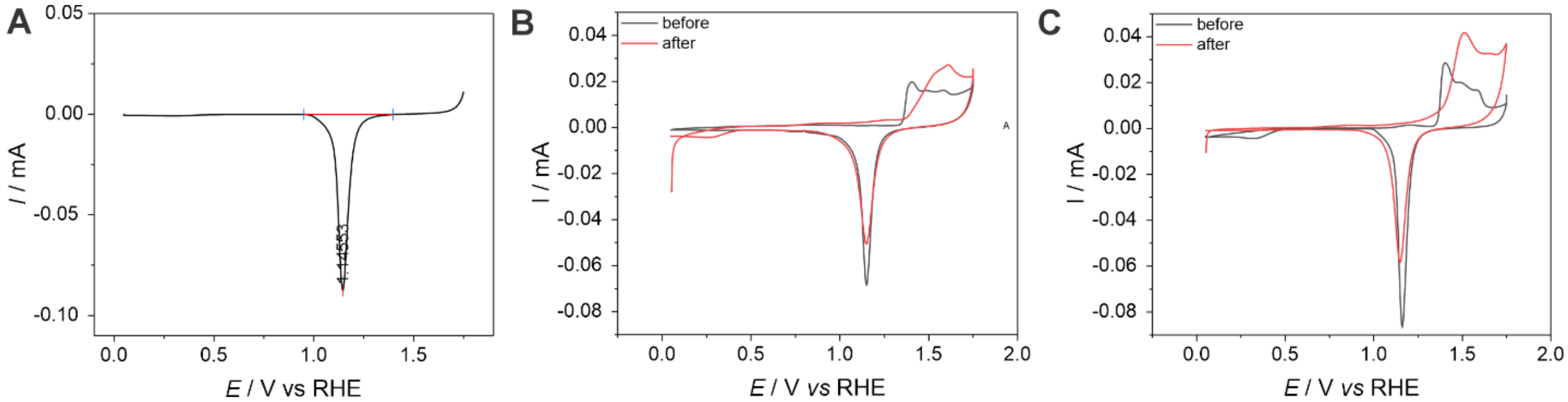

Figure S5. ECSA for current normalization. A. Exemplary depiction of the integrated area for ECSA determination. CVs in H_2SO_4 (0.1 M) before (black trace) and after (red trace) the electrochemical HER experiment depicted in **Figure 1 B** for the uncoated (**B**) and the coated (**C**) case. In **C** the black trace represents a bare Au before adding the polymer coating. The decreases in accessible surface area are most likely caused by a combination of active site blocking by the polymer and by adsorbing phosphate ions.

Supplementary Section 2 – Synthesis and Characterization of the Fluorescent Polymer 6-Carboxyfluorescein polyethylene imine (6-CF-PEI)

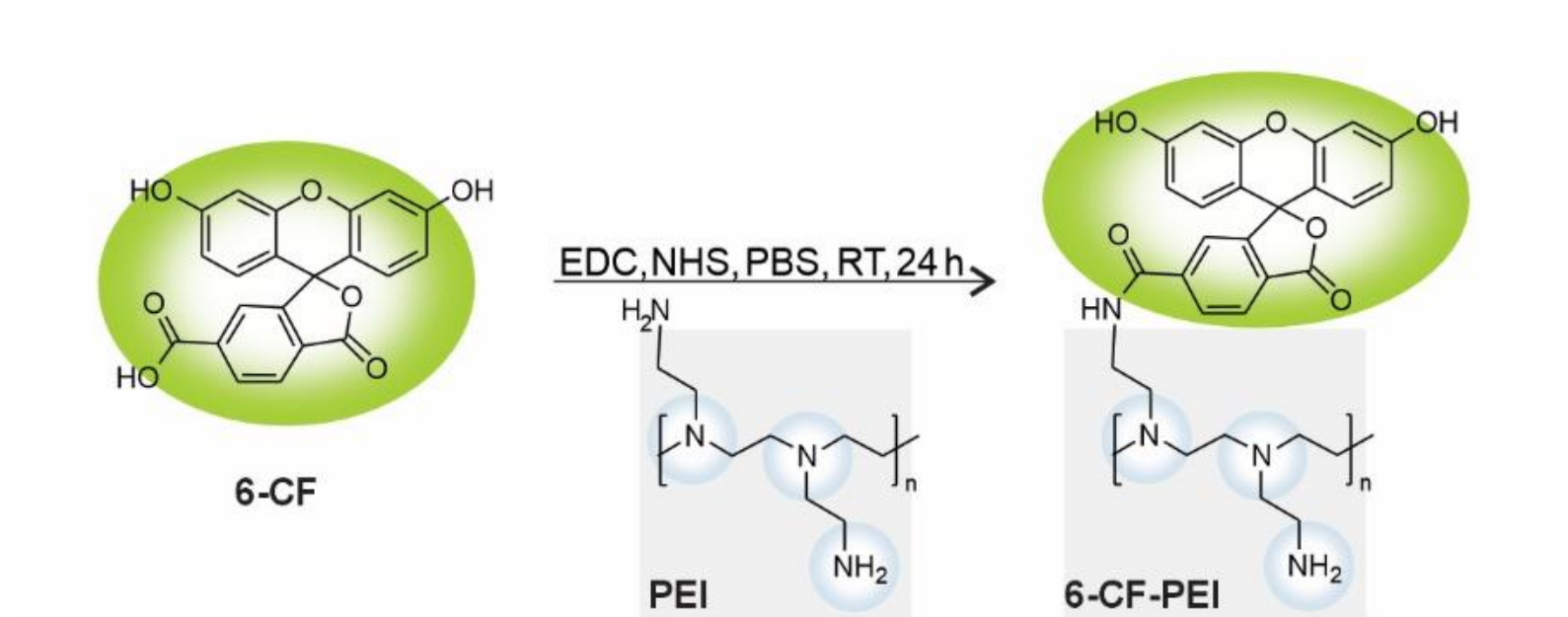

Scheme S2. Synthesis of fluorescent polymer 6-CF-PEI with abbreviations EDC: 1-Ethyl-3-[3-dimethylamino-propyl]carbodiimide, NHS: N-hydroxysuccinimide, PBS: phosphate buffer saline.

The synthesis of the polymer was adapted from a literature-known procedure[1]: To a solution of 6-carboxyfluorescein (m = 50 mg, M = 376.32 g·mol^{-1}, n = 0.13 mmol) in phosphate buffer saline (PBS, 21 ml, 0.15 M Na-PB, 0.15 M NaCl, pH 6.2), an excess of N-Hydroxysuccinimide (m = 261 mg, M = 115.09 g mol^{-1}, n = 2.3 mmol) and 1-Ethyl-3-[3-dimethylaminopropyl]carbodiimide (m = 435 mg, M = 191.7 g mol^{-1}, n = 2.3 mmol) was added. The mixture was stirred at RT for 165 minutes until a bright orange suspension had formed. A solution of PEI in PBS (1.63 mL, 13 mg ml^{-1}) was added and the solution was allowed to stir at RT for another 24 h. The resulting suspension was transferred into a centrifugation tube and the liquid phase was separated from the precipitate by centrifugation at 4,000 rcf for 1 minute. The solution was concentrated in a Vivaspin filtration tube (V = 40 mL, pore size 100 kDa) and washed with Na_2SO_4 solution (1 M, 10 x 35 mL), followed by MilliQ-water (10 x 35 mL). The solution was dried under vacuum delivering the target polymer (8.4 mg, 22 % yield).

The polymer was characterized by ^{1}H-NMR spectroscopy in D_2O (**Figure S6 A**). The spectrum revealed the presence of both, the fluorophore signals in the aromatic region (green boxes) and the polymer signals in the aliphatic region (gray boxes). Stable, covalent binding between 6-CF and

the polymer backbone was ensured by changing the NMR solvent D_2O/H_2O (1:9). Under these conditions a triplet at δ = 5.32 ppm was assigned to the newly formed amide proton (**Figure S6 B**, **C**). This was corroborated by H,H-COSY-NMR-spectroscopy showing the ^3J-coupling between the amide proton and the neighboring CH_2-group of the polymer backbone (**Figure S6 D**). The integration value of 0.1 of the amide protons implies that only every 10th amide group carried a proton. This can be explained by the excess of secondary amines in the polymer backbone. Their reaction with the fluorophore results in tertiary amides that do not carry a nitrogen-bound proton.

Interrelation of current loss and amine blocking.

In addition to structural characterization, we applied ^1H-NMR-spectroscopy to investigate if the current loss after fluorophore binding (**Figure 1 B**) can be quantitatively attributed to the loss of primary amines becoming unable to contribute to proton shuttling after fluorophore binding. Using the integral ratios of the aromatic and aliphatic signals of the NMR-spectrum in **Figure S6 A**, we estimated a ~4 % decrease of free amino/ammonium groups. The integral value of the triplet at 5.32 ppm assigned to the amide (0.1, **Figure S6 C**) suggests that 10 % of the fluorophore-binding occurs via primary amines, while the rest binds to the more nucleophilic secondary amines. Given that 25 % of the overall amines in the polymer are primary amines, it follows that ~1.6 % of the primary amines are engaged in fluorophore binding. The average current decrease after the addition of the fluorophore at E_{app} = -0.5 V *vs.* RHE, however, was 15 %. This discrepancy is too big to be solely assigned to the loss of primary amines. Possible contributions to the current loss are: (i) a significant involvement of the secondary amines in proton-shuttling as corroborated by **Figure S19 E** and **F** becoming inaccessible for (de)protonation after fluorophore binding, (ii) a disruption of the dense hydrogen bond network by the bulky fluorophore, (iii) changes in

hydrophilicity/hydrophobicity and (iv) a decrease of conformational flexibility of the polymer chains.

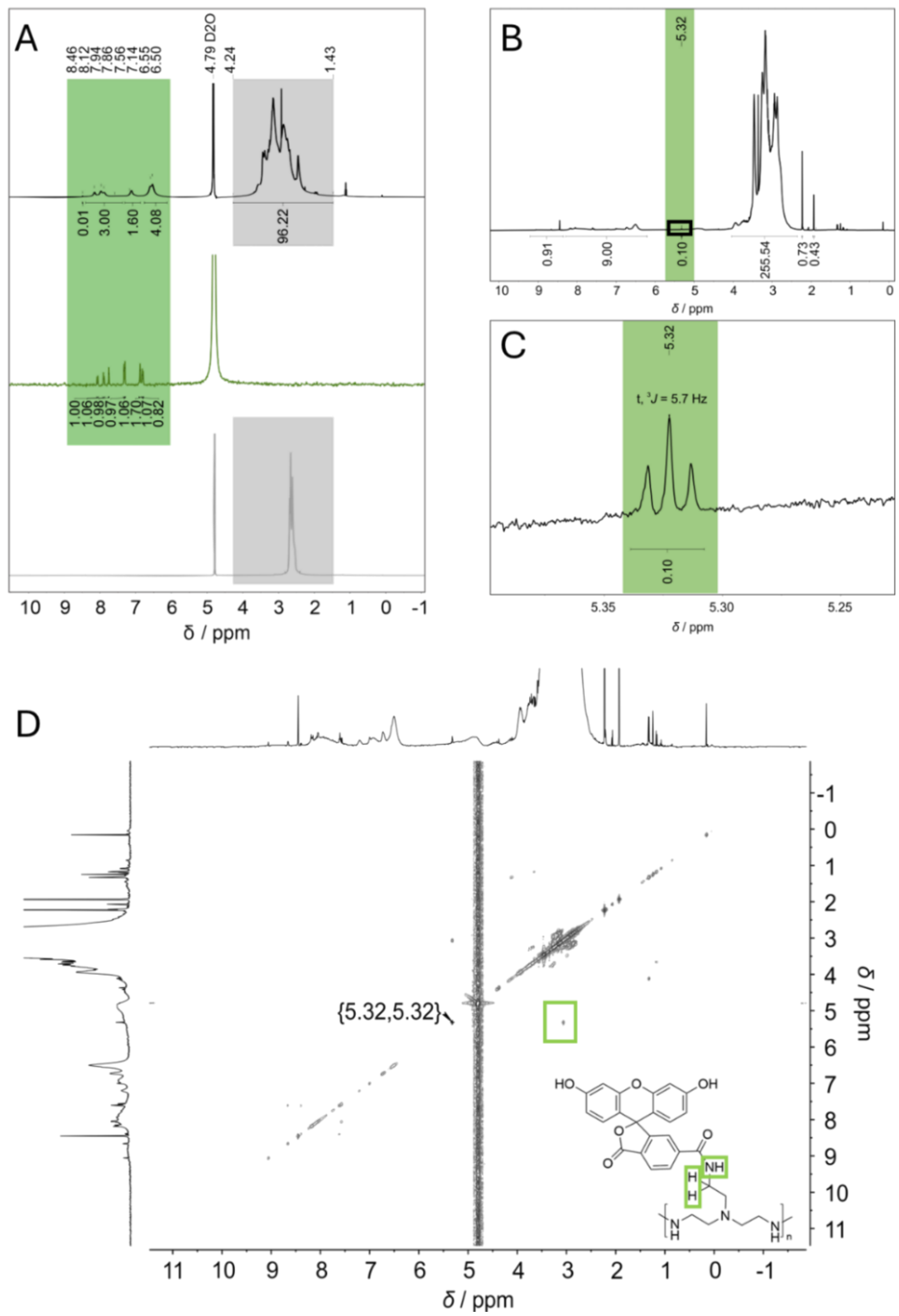

Figure S6. NMR-characterization of 6-CF-PEI. A. Comparison of the ^{1}H-NMR-spectra (*Bruker AV-400 liquid*, 400 MHz, D_2O) of 6-CF-PEI (top panel) with the starting materials 6-CF (middle panel) and PEI (bottom panel) shows the relevant proton signals of the fluorophore (green area) and of the polymer backbone (gray area). **B.** ^{1}H-NMR (*Bruker AV-III-600,* 600 MHz) in H_2O/D_2O 9:1 resolved the amide proton at 5.32 ppm (green area) with a coupling constant ^3J = 5.7 Hz (see zoomed in panel **C**) being in agreement with reported values for amides.[2] The low integral of 0.1 is most likely due to a high amount of polymer being attached to the more nucleophilic secondary amines. The resulting secondary amide lacks a proton for characterization. **D**. H-H COSY 2D-NMR (*Bruker AV-III-600,* 600 MHz) showing the ^3J-coupling between the amide proton and the neighboring CH_2-group of the polymer backbone (green boxes).

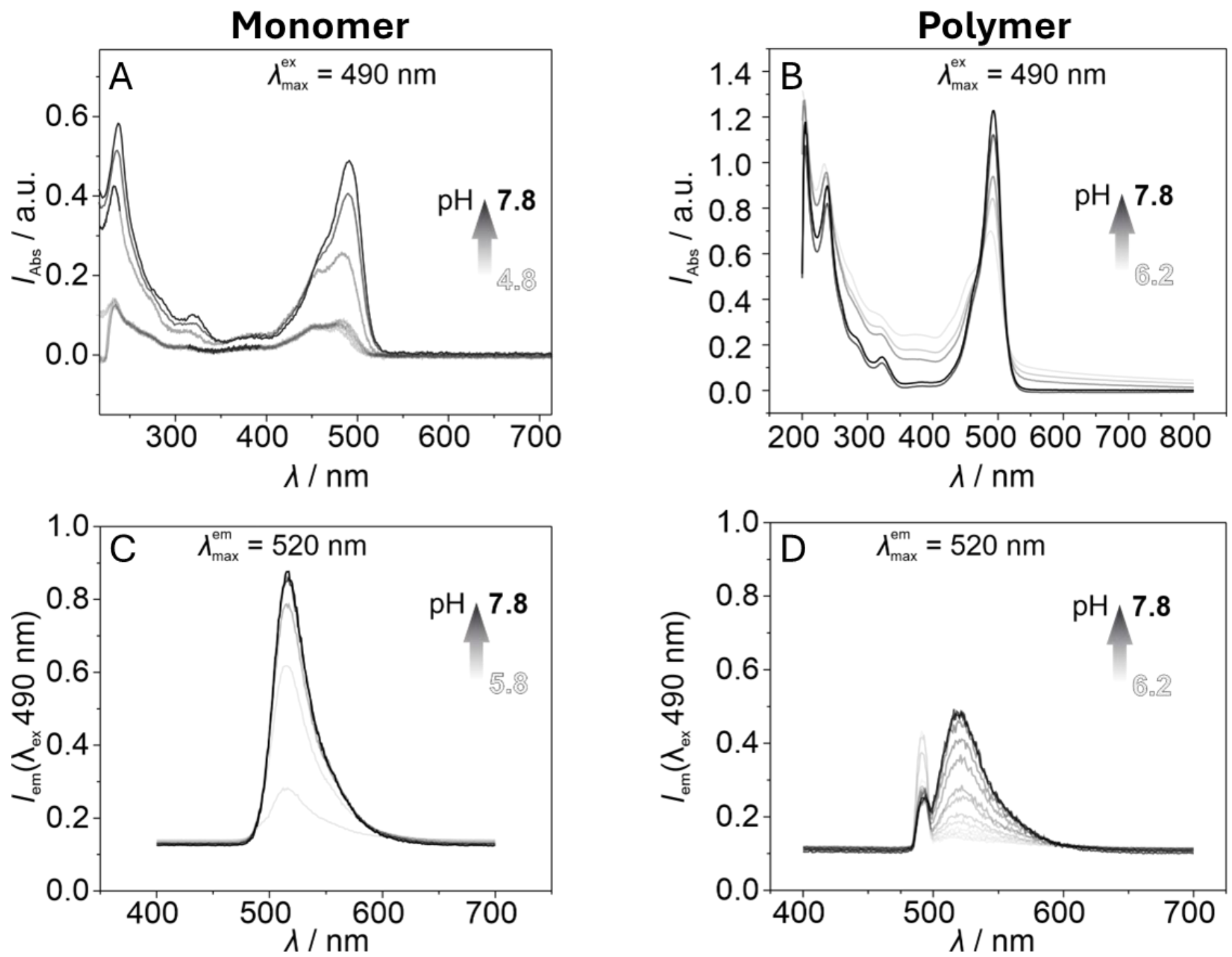

Figure S7. Comparison of monomer and polymer spectra. Absorption (**A**, **B**) and Emission (**C**, **D**) spectra (λ_{ex} = 490 nm) of 6-CF (**A**, **C**) and 6-CF-PEI (c_m = 60 µg/mL) (**B**, **D**) dissolved in potassium phosphate/citrate buffer (0.05 M). Both, emission and absorption signals increase in intensity with increasing pH (gray to black traces). Spectra were recorded at a *Jasco J-1500 CD* spectrometer at a detector voltage of HT = 950 V and a digital integration time of 250 ms.

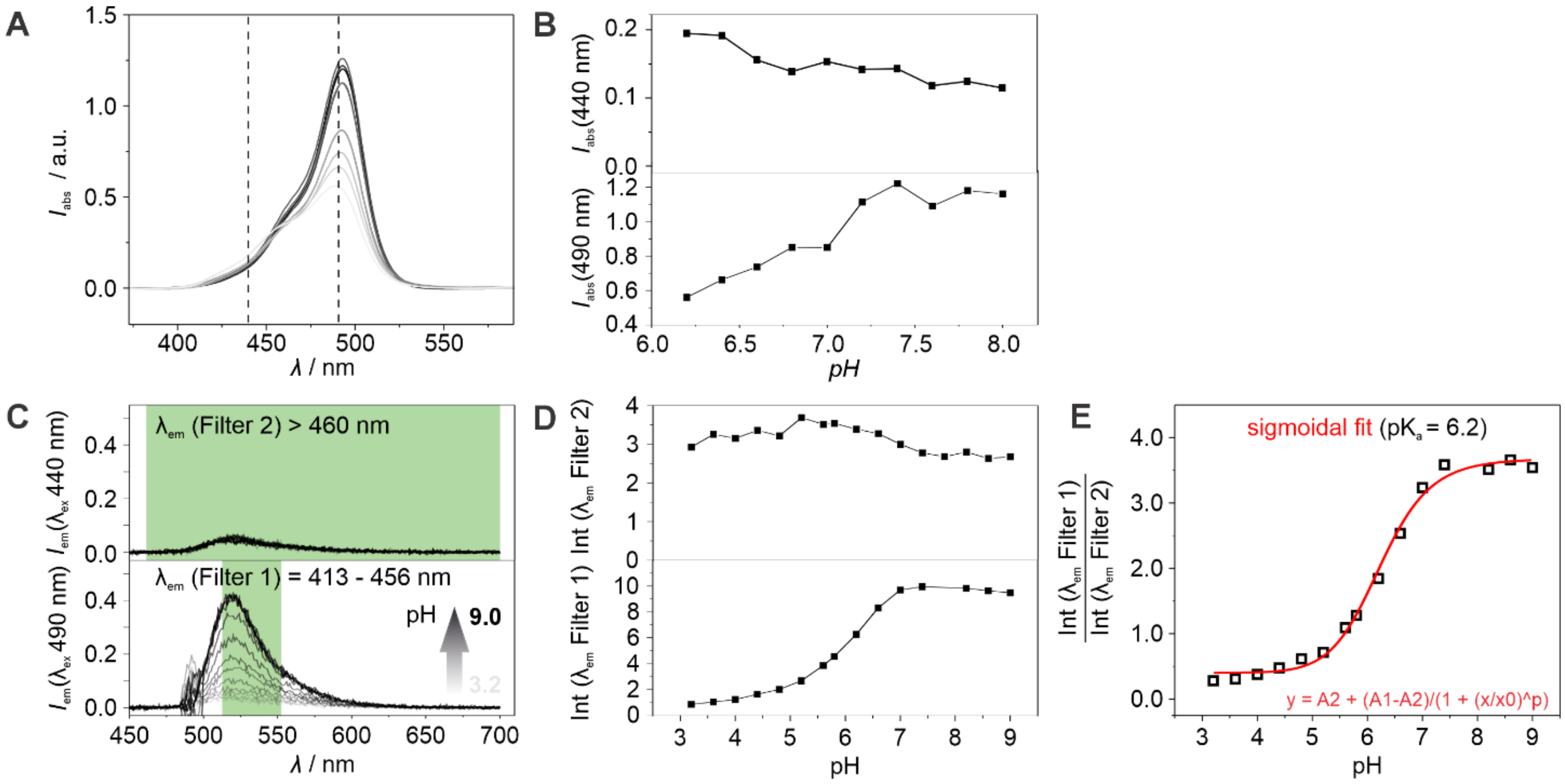

Figure S8. pH-response of 6-CF-PEI in solution. **A**. Background corrected absorption spectra of 6-CF-PEI (c_m = 60 µg/mL) in potassium phosphate buffer (0.1 M). The dashed, vertical lines indicate λ = 440 nm and λ = 490 nm and the intensities at these wavelengths were plotted *vs.* pH in panel **B**. **C.** Emission spectra of 6-CF-PEI (c_m = 12 µg/mL) dissolved in potassium phosphate/citrate buffer (0.05 M) at λ_{ex} = 490 nm (lower panel) or λ_{ex} = 440 nm (upper panel). The peaks of the emission spectra were integrated over the wavelength regions highlighted by the green boxes. These wavelengths correspond to the wavelength cut-offs of the filter cubes used in fluorescence microscopy. The integral values obtained from **C** are plotted *vs.* pH in panel **D**, showing a sigmoidal pH response at λ_{ex1} and no systematic pH trend at λ_{ex2} = 440 nm. This agrees with the trends seen in the absorption spectra. The integral values at λ_{ex2} were used for normalization leading to the ratiometric calibration curve in panel **C**. The data points (black, hollow squares) of 6-CF-PEI were fitted by a logistic function (red line) exhibiting an inflection point at pH 6.2. This corresponds to the fluorophore's pK_a, which had not changed significantly after polymer binding. The logistic function used for fitting is given in panel **E**.

Supplementary Section 3 – Fluorescence Microscopy

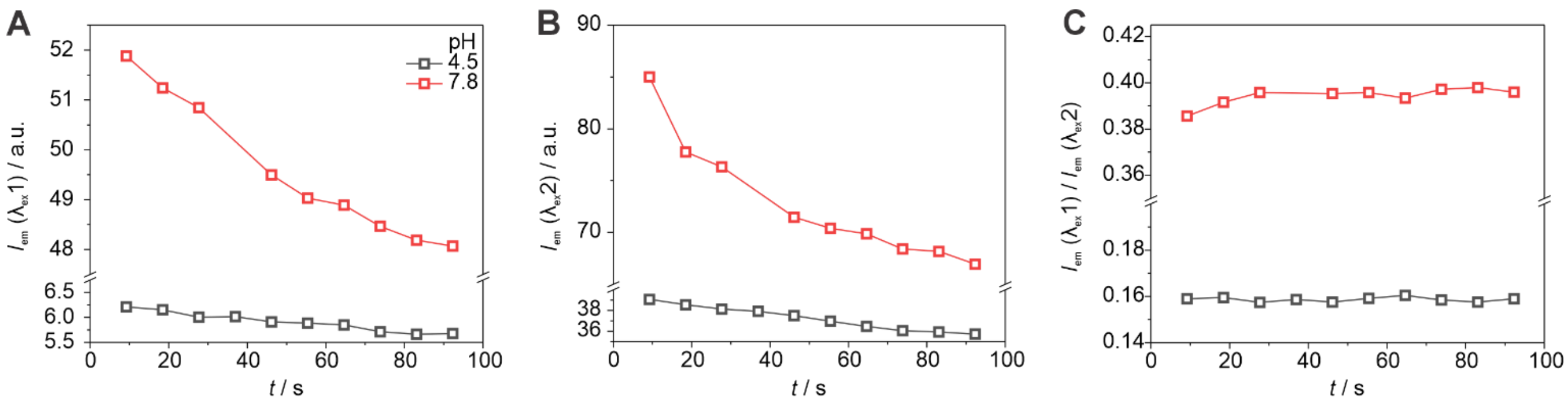

Figure S9. Robustness of ratiometric pH-read-out against photobleaching. Mean intensities extracted from images collected at λ_{ex1} = 490 nm (**A**) and λ_{ex2} = 440 nm (**B**) during widefield fluorescence experiments at 25 % light exposure at two exemplary pH values as indicated in the panels. **C**. Constant intensity ratio over time.

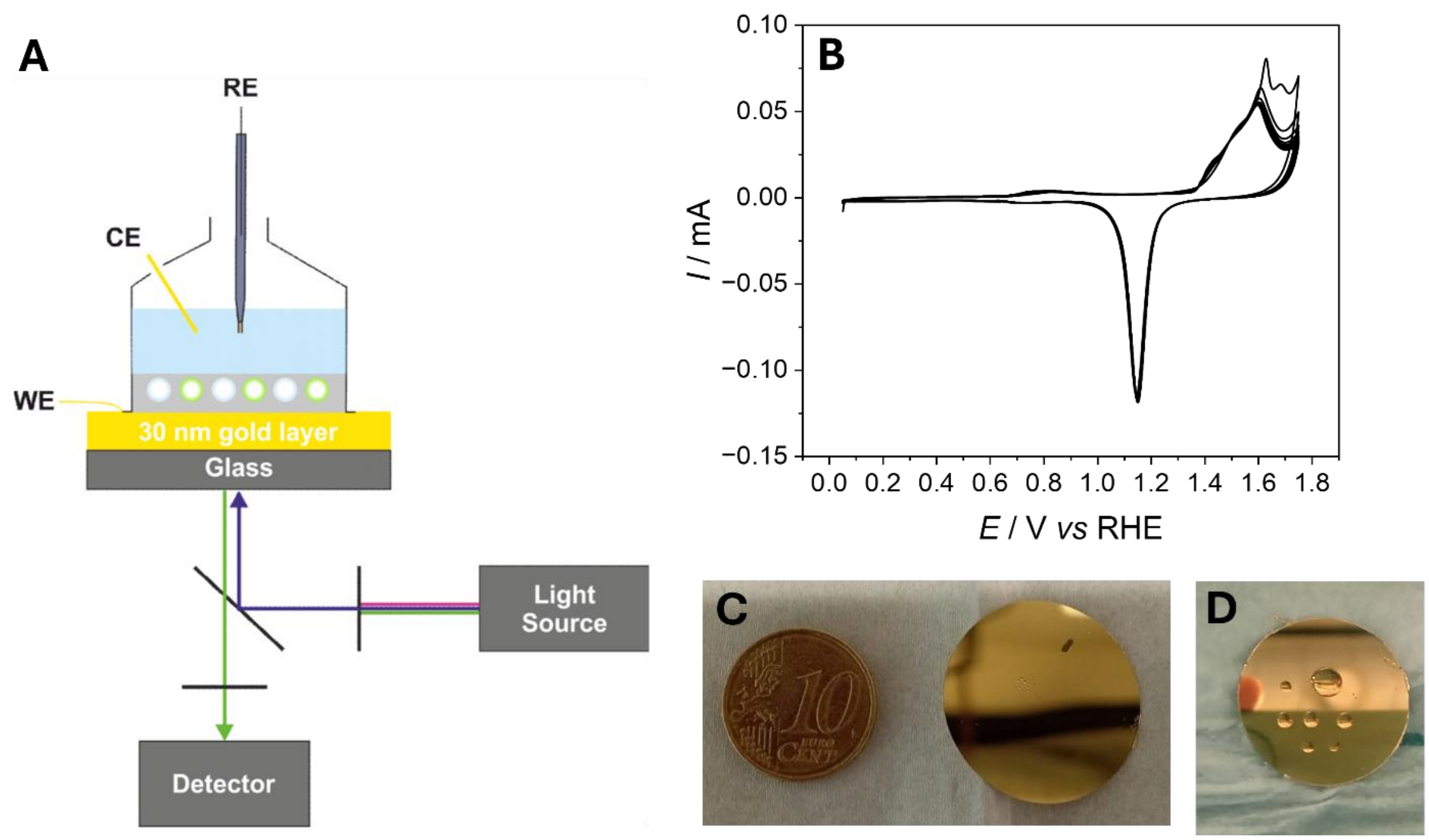

Figure S10: Technical details on operando electrochemical microscopy. A. Schematic Representation of the experimental setup showing the measuring cell arrangement and the light path. **B**. CV (ν = 50 mV/s, 10 cycles) of the bare microscope electrode in H_2SO_4 (0.1 M). The CV shows the characteristics of a polycrystalline Au surface including the AuO reduction signal at 1.15 V. **C.** Photograph of the WE (diameter = 2.5 cm) with a 10 Eurocent coin as size reference. **D**. Exemplary image of a WE after drop-coating with the fluorescent polymer solution before drying.

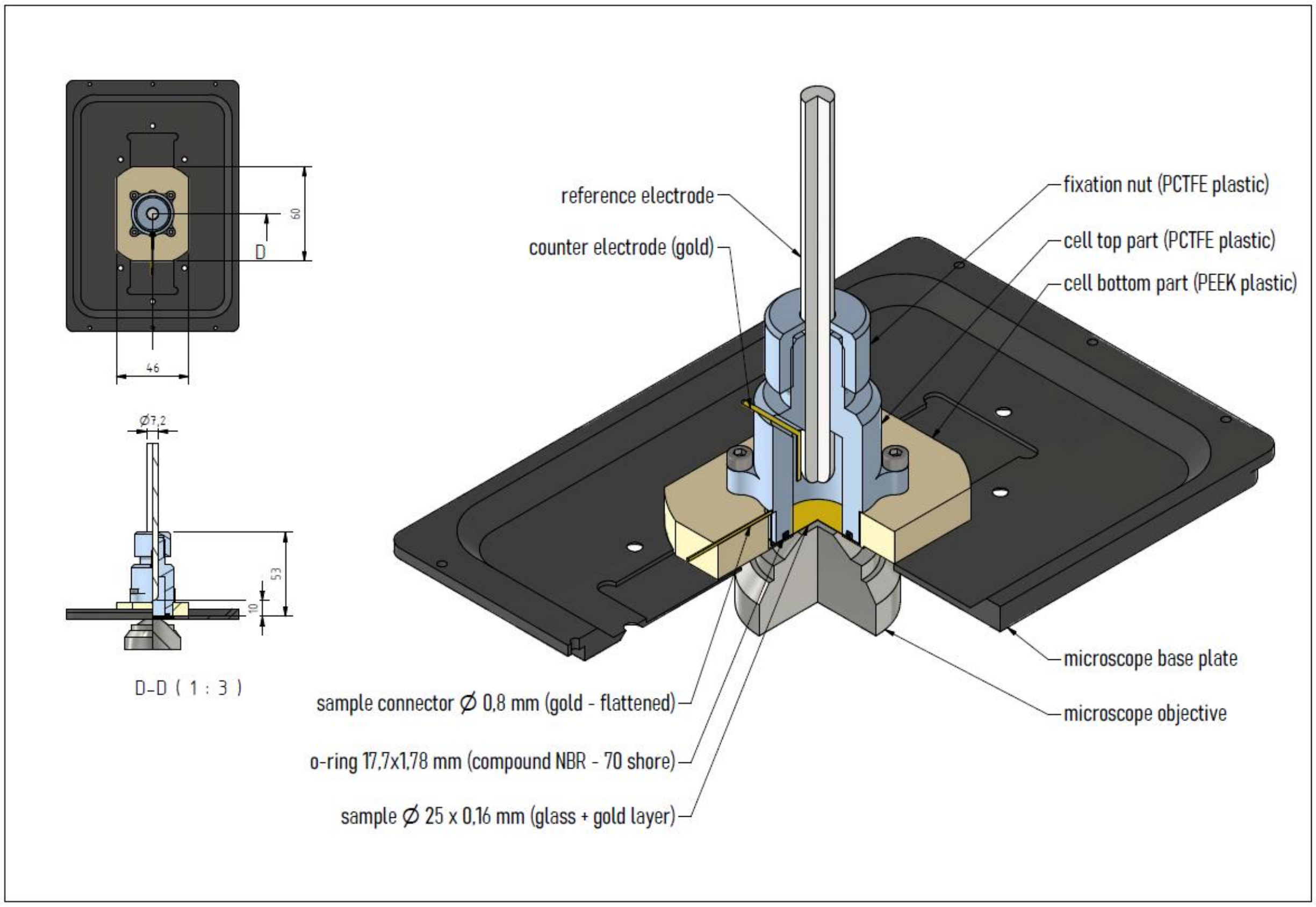

Figure S11. Details on measuring cell materials and dimensions.

The electrochemical cell is composed of two parts, a cylindrical reaction vessel made of a semi-transparent polychlorotrifluoroethylene (PCTFE) and a polyether ether ketone (PEEK) bottom part. The bottom part has a rectangular outer shape (60 x 46 x 10 mm) with its edges cut off. This characteristic shape is compatible with the *Nikon* microscope base plate. In the middle, it has a 25 mm broad hole, which narrows to 17.7 mm at the bottom to be compatible with a NBR O-ring for sealing (17.7 x 1.78 mm, 70 shore). Below the O-ring, the WE is positioned. A groove in the bottom plate allows connecting the WE with a flattened Au-wire (*Matec*, diameter 0.8 mm, 99.99 % purity). The measuring cell has four fixation nuts to tighten it on the working electrode. The preferred electrolyte volume was 3 mL resulting in a filling height of ~1.5 cm. At the top, the vessel tapers. At this edge, a 2.5 mm hole is drilled that allows insertion of a Au wire (*Matec*, thickness: 1 mm) serving as the counter electrode. The end of the wire is positioned ~ 1 cm above

the WE. Above the tapering, the cell is equipped with a screw cap. The screw cap has a hole compatible with a *Gaskatel Mini-Hydroflex* RE. The screw cap allows varying the distance between the tip of the RE and the WE and is adjusted to ~1 cm throughout our experiments. The hole for the RE is also used to introduce a Teflon tube (1 mm diameter) for degassing the electrolyte with N_2.

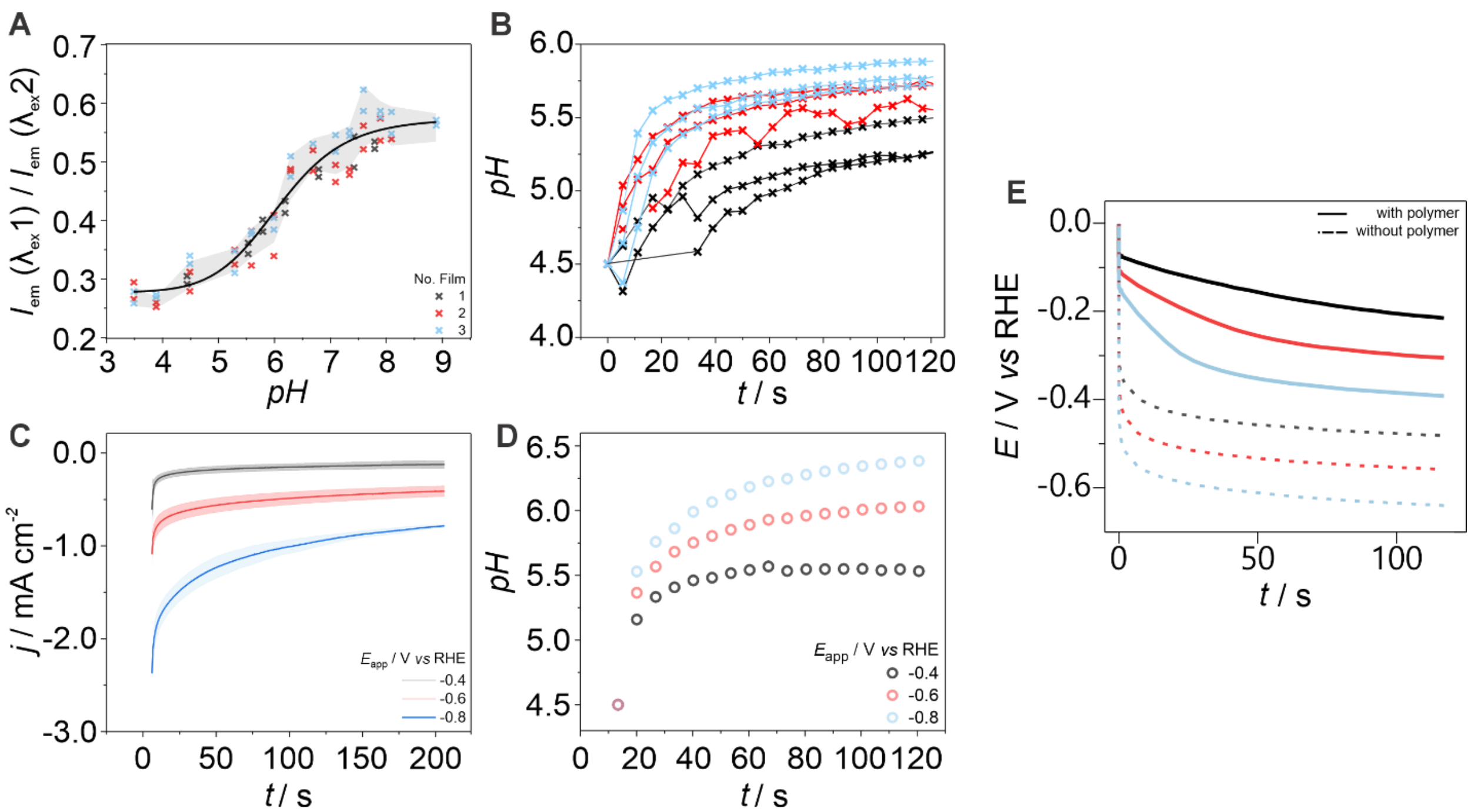

Figure S12: Supporting measurements for Figure 2. A. Representation of individual measuring points leading to the calibration curve in **Figure 2 C**. The calibration was reproducible from three individual films using two positions each. **B**. Non-averaged representation of the pH-measurement in **Figure 2 E**. **C**. Chronoamperometries (CAs) at three different potentials as indicated in the legend. At these high potentials, contribution of both, polymer-covered and uncovered gold areas is expected. Therefore, the currents were normalized by the full area of the microscopy-compatible electrode (A_{geo} = 3.14 cm^2). Data are given as average values (solid lines) ± standard deviation from three individual measurements of one electrode. **D**. Temporal development of the in-polymer pH values, corresponding to the CAs in panel **C**. **E**. Simulated chronopotentiometries of polymer-coated (solid lines) and bare gold electrodes (dotted lines) corresponding to the pH-values presented in **Figure 2 F**.

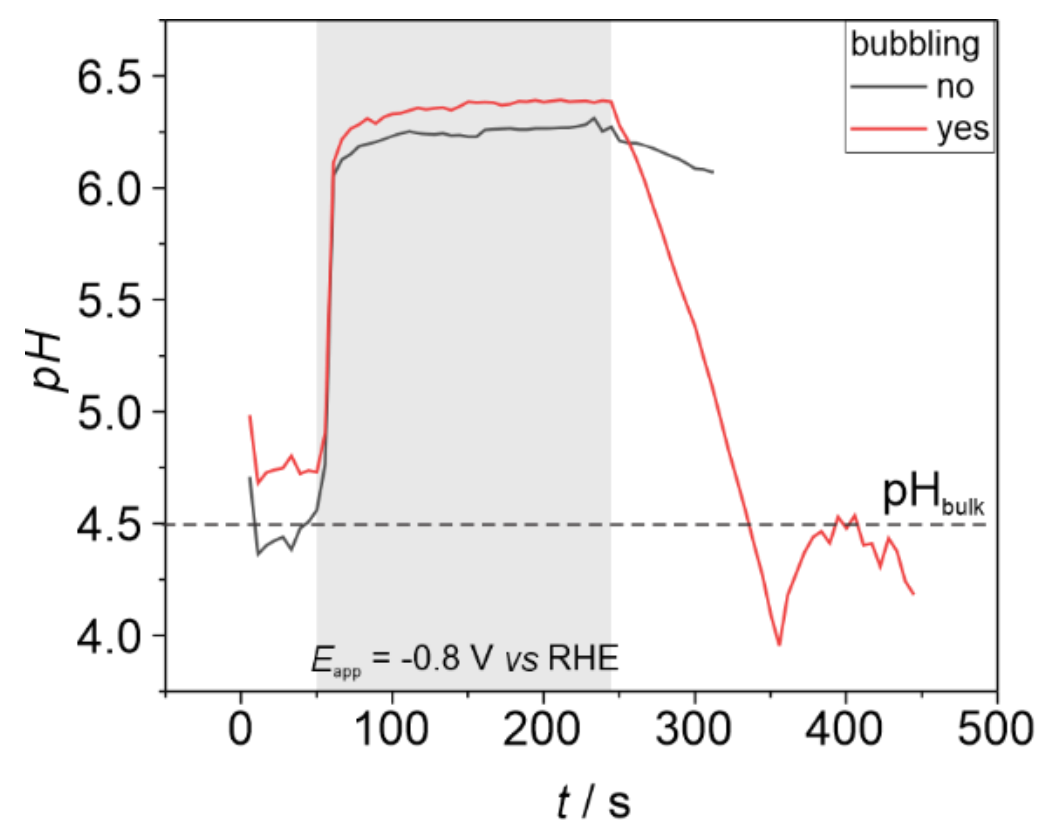

Figure S13: Influence of potential and convection. pH-values were followed by EPI fluorescence microscopy under OCP (white areas) or at an applied potential of -0.8 V *vs.* RHE (gray areas) in the presence (red) and absence (black) of forced convection induced by N_2 bubbling.

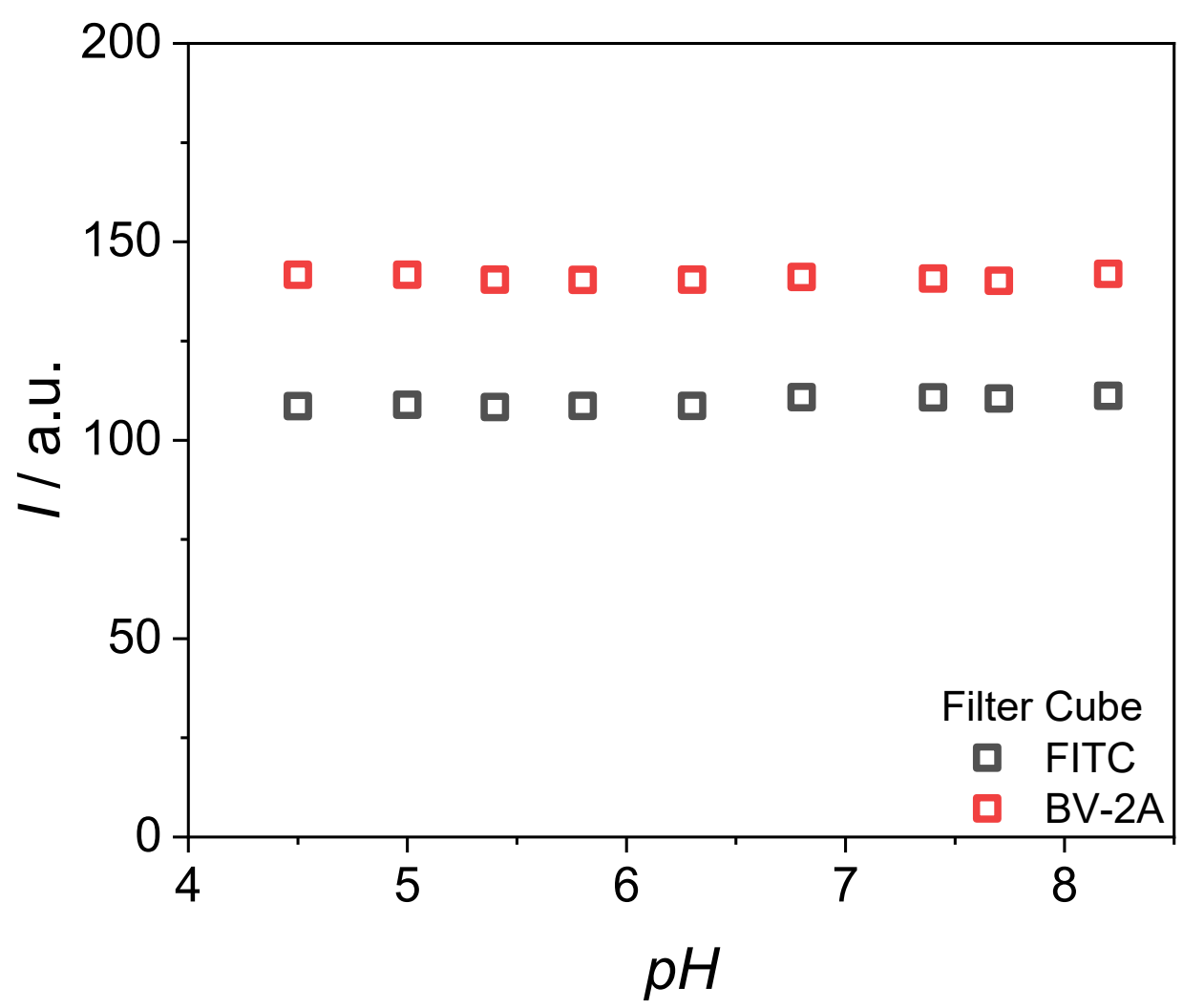

Figure S14. Determination of background emission in widefield-fluorescence mode. Images were recorded at a polymer-free position at different pH values using both filter cubes.

Supplementary Section 4 – Film Thickness Determination

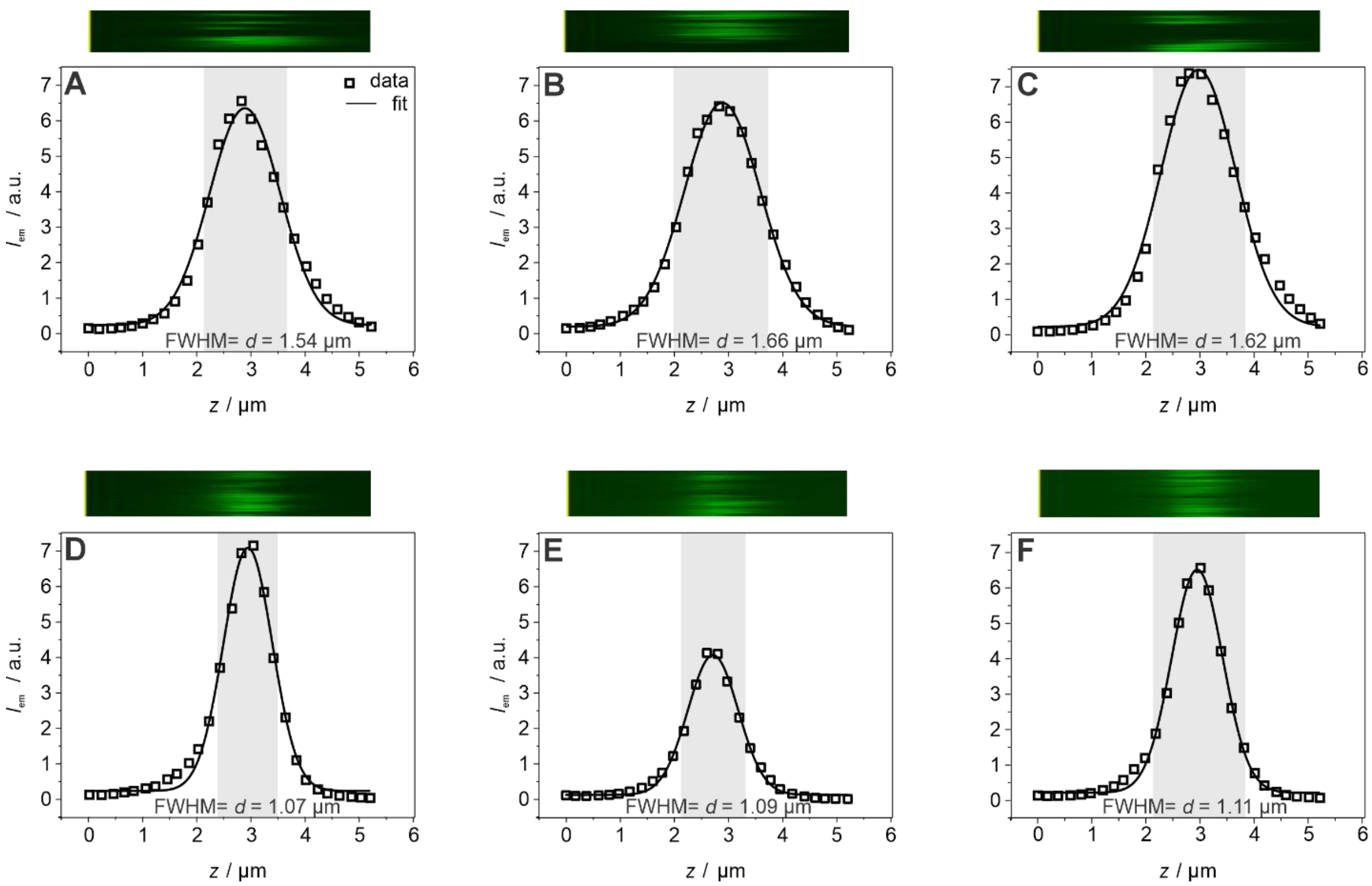

Figure S15: Thickness determination by confocal laser scanning microscopy. Mean intensities of each slice were plotted *vs.* *z*-position in the film and the intensity distribution was fitted by a Gauss function. The film thickness was determined by the full-width-half-maximum criterion. Films are inherently inhomogeneous. Two different types of positions were identified. Porous sections with a film thickness of ~ 1.6 µm (**A** – **C)** and dense positions with a thickness of ~ 1.1 µm (**D** – **F**). The corresponding orthogonal views through the polymer film are depicted on top of each panel. The images were recorded in KH_2PO_4 solution (0.1 M, pH 4.5).

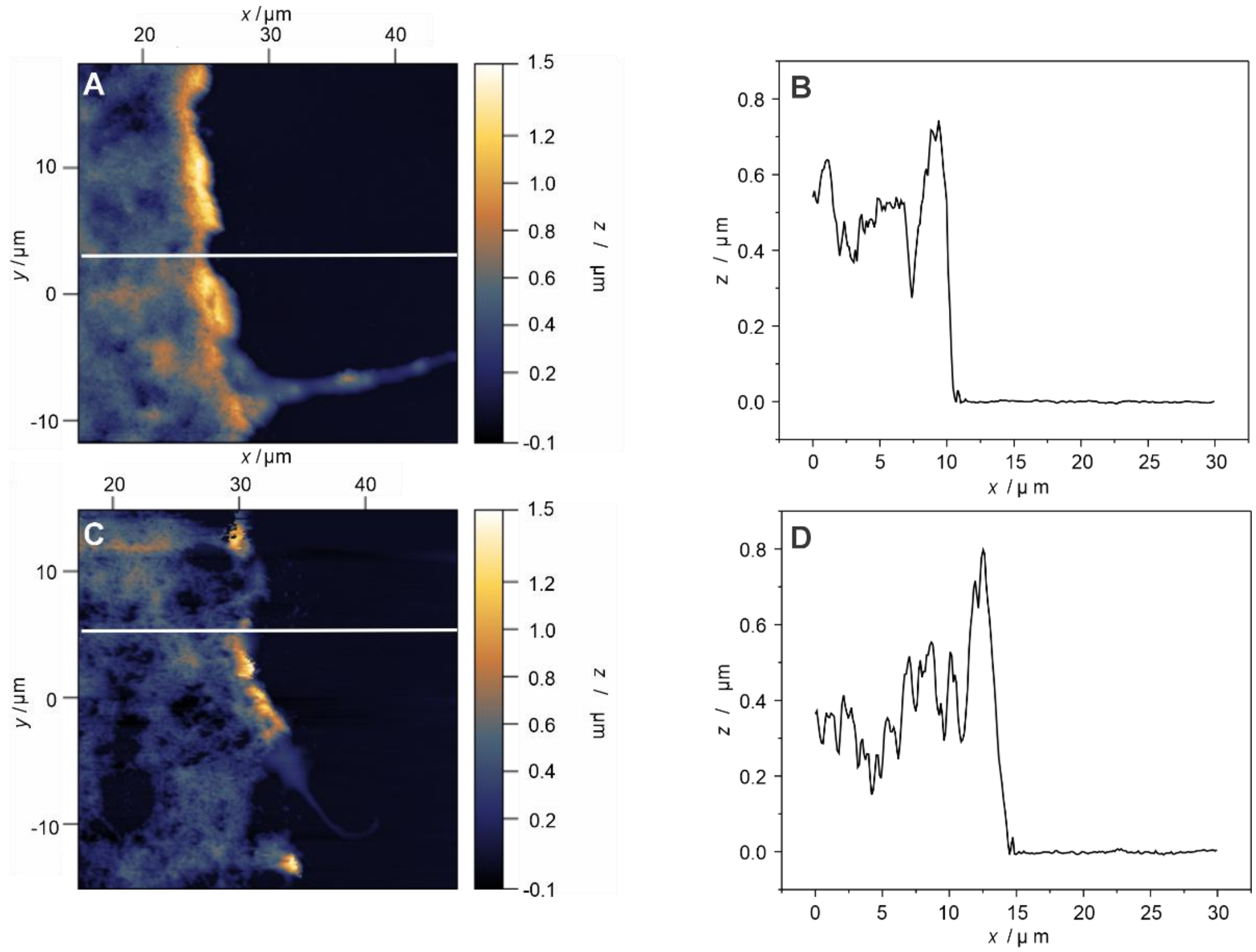

Figure S16: Thickness determination by liquid atomic force microscopy (AFM) in KH_2PO_4 solution (0.1 M, pH 4.5) for two individual films. Panels **A** and **C** show the height map with the dark, right region being the flat gold electrode and the rough polymer film at the left side. The height profile along the white lines is shown in panels **B** and **D**. AFM revealed a film thickness of ~ 0.5 µm. The non-coated Au-regions did not exhibit any significant roughness.

AFM images were recorded with a *JPK NanoWizard 4* in QI-mode, using SNL-10 probes (*Bruker*, nominal radius 2 nm). The scans were performed at Au-functionalized glass coverslips coated with the fluorescent polymer identical to the *operando* microscopy measurements (for preparation see experimental section). The polymer electrodes were partially covered with a stripe of scotch tape and crosslinked PEI films were deposited in a way that one half of the droplet was positioned at the tape, while the other half was positioned at the Au (**Figure S17 A**). The films were allowed to dry for 72 h at 4°C in a moist atmosphere. After drying, the scotch tape was ripped off, leaving behind a half circular polymer film with a sharp edge suitable for thickness evaluation (**Figure S17 B**). This procedure was applied because the "natural edges" of the polymer film are not sharp and thus prevent a reliable thickness determination within the field of view of a typical AFM

experiment. Scratching the polymer film in the middle lead to ambiguous results, likely due to moving and accumulation of material around the scratch. AFM images were recorded with the sample immersed in KH_2PO_4-solution (0.1 M, pH 4.5) (**Figure S16**) or in potassium phosphate buffer (0.1 M, pH 7.5) (**Figure S24**) showing a pH-independent thickness of ~ 0.5 µm.

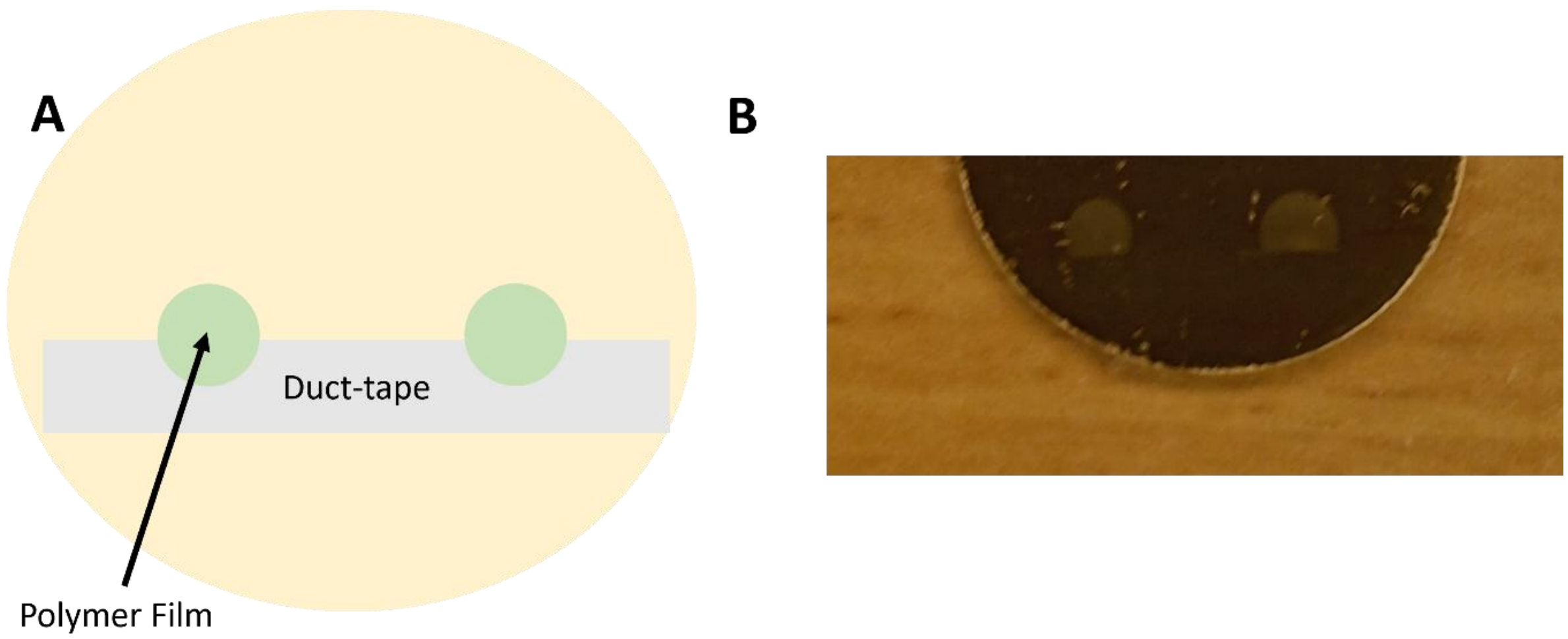

Figure S17: Preparation of films for thickness determination by AFM. A. Schematic of the drop-coating arrangement. **B**. Photograph of the polymer films after removing the duct tape. The AFM scans were recorded over the lower, horizontal edge of the polymer film.

Supplementary Section 5 – Computational Details and Parameter Extraction

To simulate the time-dependent concentration distributions across the polymer film and electrolyte solution, we used the commercial finite-element modelling software, *COMSOL Multiphysics* (*Version 6.2*). The model is designed to capture the dominant physical processes and replicate experimental trends, rather than to quantitatively match the experimental results. Mass transport was described by diffusion, with flux described by Fick's first law:

$$J_i(x,t) = -\,D_i\left[\frac{\partial c_i(x,t)}{\partial x}\right] \qquad Eq.S1$$

where $J_i(x,t)$ is the flux normal to the electrode surface, $c_i(x,t)$ is the concentration and D_i is the diffusion coefficient of species i. The species considered include the pH determining species (i = H^+ and OH^-) and phosphate buffer species (i = H_3PO_4, $H_2PO_4^-$, HPO_4^{2-}, PO_4^{3-}).

The rate of change of the concentration with time is described by the reaction-diffusion equation:

$$\frac{\partial c_i(x,t)}{\partial t} = D_i\left(\frac{\partial^2 c_i(x,t)}{\partial x^2}\right) + R_i(x,t) \qquad Eq.S2$$

where $R_i(x,t)$ is the net reaction rate of the homogenous buffer reactions. Unless otherwise stated, the values for all the parameters are summarized in **Tables S1-3.**

Geometry and Mesh

A schematic of the one-dimensional geometry for the case of the bare (**Figure S18 a**) and polymer covered electrode (**Figure S18 b**) is shown below. In both the bare and functionalized cases, the electrode boundary condition is defined at $x = 0$ and the total width of the model is defined by the parameter $L = 1$ cm. When considering the effects of the polymer the single domain of the bare electrode case was split to account for the polymer film with a thickness d. Unless otherwise stated

the film is assumed to have a thickness of 1.6 μm to correspond to the data in **Figure 3 A** of the main article.

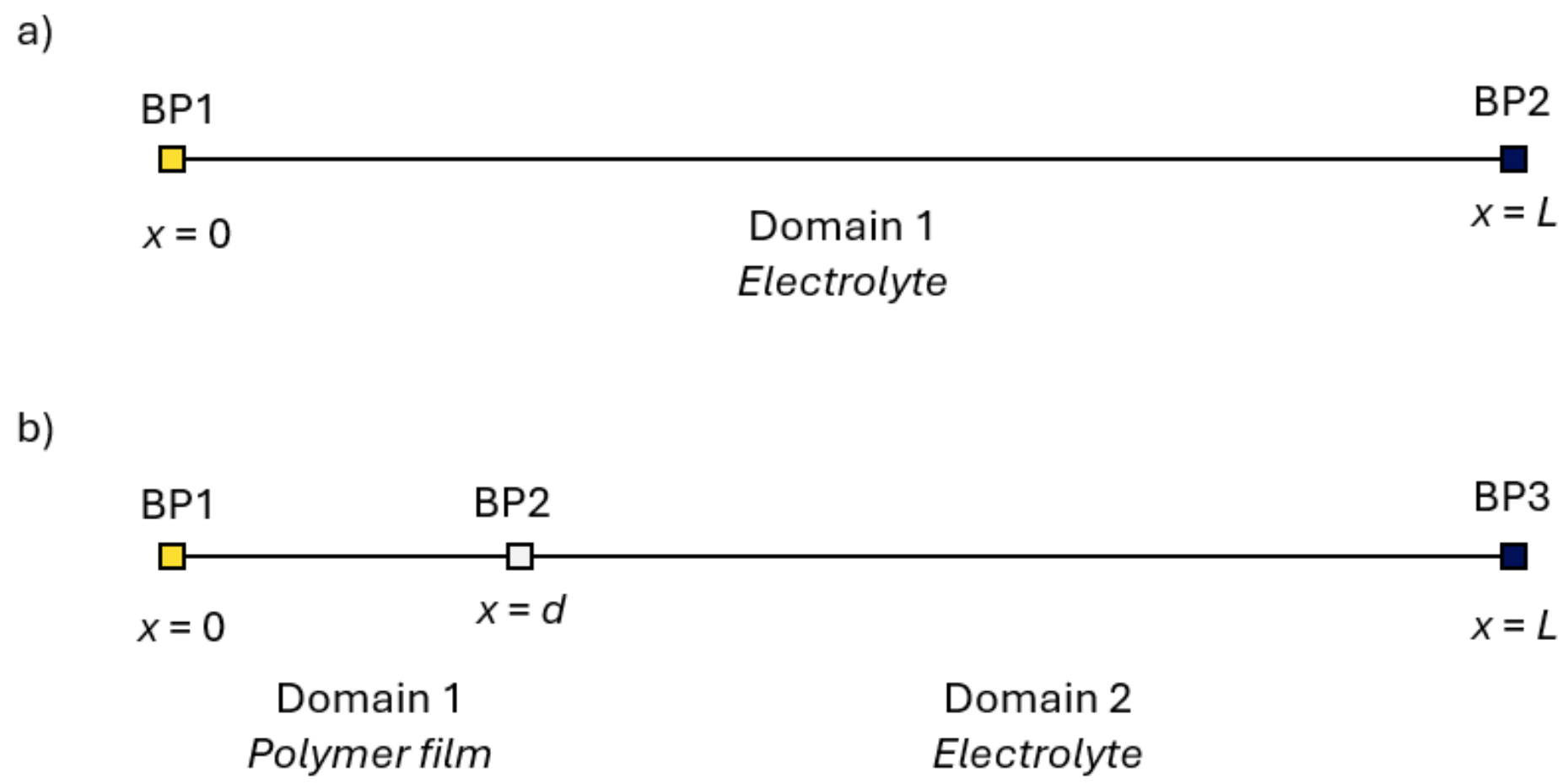

Figure S18: Schematic of the geometry used for the a) bare electrode and b) polymer film functionalized electrodes.

The simulations were solved using a mesh with a total of ~ 700 elements for both cases. The smallest element is near the electrode surface ($x = 0$), where the element size is 0.05 nm, increasing with a growth rate of 10%. The largest element size in the domain is 1/100th of the total length of the model. The equations were discretized using linear Lagrange elements.

Chemical Reaction Kinetics

The availability of protons was calculated by explicitly including the three buffer equilibria of the potassium phosphate buffer (***Eq.*** **S4 – S6**) together with the autoprotolysis (***Eq.*** **S7**) of water.[3]

$$H_3PO_4 \underset{k_{-1}}{\overset{k_1}{\rightleftharpoons}} H_2PO_4^- + H^+ \qquad pK_{a,1} = 2.16 \qquad Eq.S4$$

$$H_2PO_4^- \underset{k_{-2}}{\overset{k_2}{\rightleftharpoons}} HPO_4^{2-} + H^+ \qquad pK_{a,2} = 7.21 \qquad Eq.S5$$

$$\mathrm{HPO_4^{2-}} \underset{k_{-3}}{\overset{k_3}{\rightleftharpoons}} \mathrm{PO_4^{3-}} + \mathrm{H^+} \qquad \mathrm{pK_{a,3}} = 12.32 \qquad Eq.S6$$

$$\mathrm{H_2O} \underset{k_{-4}}{\overset{k_4}{\rightleftharpoons}} \mathrm{OH^-} + \mathrm{H^+} \qquad \mathrm{pK_{a,4}} = \mathrm{pK_w} = 14.00 \qquad Eq.S7$$

These reactions were implemented as kinetic rate expressions with finite rate constants for the forward deprotonation (k_i) and backward protonation (k_{-i}) reactions, which relate to their respective acid dissociation constants, K_{ai}.

$$K_{\mathrm{a},i} = \frac{k_i}{k_{-i}} \qquad Eq.S8$$

For the phosphate buffer reactions, the protonation rate constant was fixed for all three reactions to ensure that it remains physically realistic and below the diffusion controlled limit for a bimolecular reaction in aqueous solution. From the work of Eigen, the protonation rate constant should be in the region of ~ 1×10^{10} to 1×10^{11} M^{-1} s^{-1}.[4] Accordingly, k_{-i} was set within this range at 5×10^{10} M^{-1} s^{-1} and the corresponding deprotonation rate constant was calculated from the equilibrium constant using ***Eq.* S8**. For the autoprotolysis of water k_4 and k_{-4} correspond to the rate of dissociation and recombination of water, with values 2.5×10^{-5} s^{-1} and 1.4×10^{11} M^{-1} s^{-1} respectively.[4]

The individual chemical reactions for the phosphate buffer and water ionization are defined using the following rate equations for $0 \leq x \leq L$:

$$r_{\mathrm{H_3PO_4}} = k_1(c_{\mathrm{H_3PO_4}}(x,t)) - k_{-1}(c_{\mathrm{H^+}}(x,t) \times c_{\mathrm{H_2PO_4^-}}(x,t)) \qquad Eq.S9$$

$$r_{\mathrm{H_2PO_4^-}} = k_2(c_{\mathrm{H_2PO_4^-}}(x,t)) - k_{-2}(c_{\mathrm{H^+}}(x,t) \times c_{\mathrm{HPO_4^{2-}}}(x,t)) \qquad Eq.S10$$

$$r_{\mathrm{HPO_4{}^{2-}}} = k_3(c_{\mathrm{HPO_4^{2-}}}(x,t)) - k_{-3}(c_{\mathrm{H^+}}(x,t) \times c_{\mathrm{PO_4^{3-}}}(x,t)) \qquad Eq.S11$$

$$r_{\mathrm{water}} = k_4(c_{\mathrm{H_2O}}{}^{*}) - k_{-4}(c_{\mathrm{H^+}}(x,t) \times c_{\mathrm{OH^-}}(x,t)) \qquad Eq.S12$$

Eqs.* S9-12** are used to formulate the reaction source terms in Fick's second law (Eq.* S2**) as

follows:

$$R_{H^+}(x,t) = r_{H_3PO_4} + r_{H_2PO_4^-} + r_{HPO_4{}^{2-}} + r_{water} \qquad Eq. S13$$

$$R_{OH^-}(x,t) = r_{water} \qquad Eq. S14$$

$$R_{H_3PO_4}(x,t) = -\, r_{H_3PO_4} \qquad Eq. S15$$

$$R_{H_2PO_4{}^-}(x,t) = r_{H_3PO_4} - r_{H_2PO_4^-} \qquad Eq. S16$$

$$R_{HPO_4{}^{2-}}(x,t) = r_{H_2PO_4^-} - r_{HPO_4{}^{2-}} \qquad Eq. S17$$

$$R_{PO_4{}^{3-}}(x,t) = r_{HPO_4{}^{2-}} \qquad Eq. S18$$

In the polymer layer (Domain 1 of **Figure S18**), the amino side groups act as a locally confined buffering reservoir via the protonation/deprotonation reactions shown in ***Eqs.* S19-21**.

$$\mathrm{RNH_3^+} \underset{k_{-5}}{\overset{k_5}{\rightleftharpoons}} \mathrm{RNH_2} + \mathrm{H^+} \qquad \mathrm{p}K_{a5} = 11 \qquad Eq. S19$$

$$\mathrm{R_2NH_2^+} \underset{k_{-6}}{\overset{k_6}{\rightleftharpoons}} \mathrm{R_2NH} + \mathrm{H^+} \qquad \mathrm{p}K_{a6} = 7 \qquad Eq. S20$$

$$\mathrm{R_3NH^+} \underset{k_{-7}}{\overset{k_7}{\rightleftharpoons}} \mathrm{R_3N} + \mathrm{H^+} \qquad \mathrm{p}K_{a7} = 5 \qquad Eq. S21$$

The species are modelled as immobile, with their diffusion coefficients fixed as 0 m^2s^{-1}. The total concentration of the side groups ($c_{\mathrm{Amine,tot}}$) was set as 3.8 M, estimated from the size of the film and the amount of the polymer drop cast onto the electrode.

According to literature, the polymer exhibits three distinct pK_a regions of 4.5 – 5 , 6.7 – 8.5 and 10.5 – 11.6 with a molar ratio of 1:2:1 corresponding to the distribution of primary, secondary and tertiary amino-groups.[5,6] For the sake of simplicity, these broad pK_a regions were narrowed down to three pK_a values of 5, 7 and 11. The chemical reactions are defined with the following rate equations for $0 \leq x \leq d$:

$$r_{\text{primary}} = k_5(c_{\text{RNH}_3^+}(x,t)) - k_{-5}(c_{\text{H}^+}(x,t) \times c_{\text{RNH}_2}(x,t)) \qquad Eq.S22$$

$$r_{\text{secondary}} = k_6(c_{\text{R}_2\text{NH}_2^+}(x,t)) - k_{-6}(c_{\text{H}^+}(x,t) \times c_{\text{R}_2\text{NH}}(x,t)) \qquad Eq.S23$$

$$r_{\text{tertiary}} = k_7(c_{\text{R}_3\text{NH}^+(x,t)}) - k_{-7}(c_{\text{H}^+}(x,t) \times c_{\text{R}_3\text{NH}}(x,t)) \qquad Eq.S24$$

Electron Transfer Reactions

We assume the HER can proceed via both the proton reduction and water reduction pathways, as described by ***Eqs.* S25 and S26**.

$$2H^+ + 2e^- \rightarrow \text{H}_2 \qquad Eq.S25$$

$$2\text{H}_2\text{O} + 2e^- \rightarrow \text{H}_2 + 2\text{OH}^- \qquad Eq.S26$$

Using a simple Butler-Volmer formalism of the electron transfer kinetics and assuming that the Volmer step is rate determining on gold, we use ***Eq.* S27** to describe the rate of the proton reduction pathway, r_{PR} to generate hydrogen. This rate depends explicitly on the local concentration of protons at the electrode surface, $c_{\text{H}^+}(x = 0)$:

$$r_{\text{PR}} = c_{\text{H}^+}(0,t) k_{\text{PR}}^0 \exp\left(-\frac{\alpha_{PR} F \eta}{RT}\right) \qquad Eq.S27$$

where k_{PR}^0 is the rate constant and α_{PR} is the charge transfer coefficient for the proton reduction pathway. F, R and T denote Faraday's constant, the ideal gas constant and temperature respectively. The overpotential, η, is defined as $E(t) - E^0$, where $E(t)$ is the applied electrode potential and all potentials are reported versus the RHE.

When relating the rate of electron transfer to the flux of protons it is important to consider that in *COMSOL*:

$$-\mathbf{n}.\mathbf{J} = J_i \qquad Eq.S28$$

where $\mathbf{n}$ is the outward normal unit vector on the boundary and $\mathbf{J}_i$ is the flux vector. J_i therefore

represents the flux of species i into the electrolyte domain. This means when $J_i < 0$ species are consumed and when $J_i > 0$ species are generated. The flux of protons is defined as:

$$J_{\mathrm{H^+}}(0,t) = -r_{\mathrm{PR}} \qquad Eq.S29$$

For the water reduction pathway, hydroxide ions are generated at the electrode surface modifying the local pH. We assume that water is present in large excess; as a result, the concentration of water remains effectively constant throughout the simulation and the bulk concentration of water $c_{\mathrm{H_2O}}{}^*$ is used in ***Eq.* S30**.

$$r_{\mathrm{WR}} = c_{\mathrm{H_2O}}{}^* \, k_{\mathrm{WR}}^0 \exp\left(-\frac{\alpha_{WR} F\eta}{RT}\right) \qquad Eq.S30$$

The flux of OH- ions is:

$$J_{\mathrm{OH^-}}\,(0,t) = r_{\mathrm{WR}} \qquad Eq.S31$$

The total faradaic current density (j_{F}) is the sum of the current contribution from the proton reduction pathway and the water reduction pathways.

$$j_{\mathrm{F}} = F(J_{H^+} - J_{\mathrm{OH^-}}) \qquad Eq.S32$$

Boundary and Initial Conditions

In the model we assume that no phosphate species (H_3PO_4, $H_2PO_4^-$, HPO_4^{2-}, PO_4^{3-}) are consumed in the reactions at the electrode, therefore on boundary point 1 (BP1):

$$0 = J_{\mathrm{H_3PO_4}} = J_{\mathrm{H_2PO_4^-}} = J_{\mathrm{HPO_4^{2-}}} = J_{\mathrm{PO_4^{3-}}} \qquad Eq.S33$$

At the bulk boundary ($x = L$), BP2 in the bare electrode model and BP3 in the functionalized electrode model, which corresponds to the reference/counter electrode, the concentrations of all species are fixed to their bulk values by the following general expression.

$$c_i(L,t) = c_i^* \qquad Eq.S34$$

The same bulk concentrations are used to define the initial concentration of the electrolyte ions,

$$c_i(x,0) = c_i^* \qquad Eq.S35$$

with their corresponding values provided in **Table S2** when the bulk pH is 4.5 and 6.2.

Table S1: Initial and bulk concentrations of species *i* in the electrolyte solution to 3 significant digits.

Species *i*	Concentration / mM	
	Bulk pH 4.5	Bulk pH 6.2
H^+	3.16×10^{-2}	6.34×10^{-4}
OH^-	3.38×10^{-7}	1.69×10^{-5}
H_2O	55,500	55,000
H_3PO_4	0.454	8.31×10^{-3}
$H_2PO_4^-$	99.4	91.1
HPO_4^{2-}	0.194	8.90
PO_4^{3-}	2.93×10^{-9}	6.75×10^{-6}
P_{tot}	100	100

These concentrations are calculated analytically using ***Eqs.* S36-40** which are solved for in the model. Note that the pH of the initial 0.1 M KH_2PO_4 solution is modified with either 0.1 M K_2HPO_4 or 0.1 M H3PO4 to ensure the total concentration of phosphate species $c_{P,tot}$ is 0.1 M.

$$c_{P,tot} = c_{H_3PO_4} + c_{H_2PO_4^-} + c_{HPO_4^{2-}} + c_{PO_4^{3-}} \qquad Eq.S36$$

$$c_{H_3PO_4} = \frac{c_{P,tot}(c_{H^+})^3}{(c_{H^+})^3 + K_{a1}(c_{H^+})^2 + K_{a1}K_{a2}(c_{H^+}) + K_{a1}K_{a2}K_{a3}} \qquad Eq.S37$$

$$c_{H_2PO_4^-} = \frac{c_{P,tot}K_{a1}(c_{H^+})^2}{(c_{H^+})^3 + K_{a1}(c_{H^+})^2 + K_{a1}K_{a2}(c_{H^+}) + K_{a1}K_{a2}K_{a3}} \qquad Eq.S38$$

$$c_{HPO_4^{2-}} = \frac{c_{P,tot}K_{a1}K_{a2}(c_{H^+})}{(c_{H^+})^3 + K_{a1}(c_{H^+})^2 + K_{a1}K_{a2}(c_{H^+}) + K_{a1}K_{a2}K_{a3}} \qquad Eq.S39$$

$$c_{PO_4^{3-}} = \frac{c_{P,tot}K_{a1}K_{a2}K_{a3}}{(c_{H^+})^3 + K_{a1}(c_{H^+})^2 + K_{a1}K_{a2}(c_{H^+}) + K_{a1}K_{a2}K_{a3}} \qquad Eq.S40$$

The concentration of species in the polymer film is defined initially

$$c_i(x,0) = c_i^* \text{ for } 0 \le x \le d \qquad Eq.S41$$

Table S2*: Initial concentrations of protonated and deprotonated amine groups* in the polymer film to 3 significant digits. All polymer film experiments were performed and modeled at a bulk pH of 4.5.

Species *i*	Concentration / mM at bulk pH 4.5
RNH_3^+	950
RNH_2	3.00×10^{-4}
$R_2NH_2^+$	1.89×10^3
R_2NH	5.99
R_3NH^+	722
R_3NH	228

Voltammetry and Potentiometry

In the simulations, two electrochemical techniques are considered: linear sweep voltammetry (LSV) and chronopotentiometry (CP). Therefore, there are two separate boundary conditions that are implemented at the electrode surface (BP1) to account for this.

For the LSV measurements, the electrode potential is swept using the following equation,

$$E(t) = E_{\mathrm{Start}} - vt \qquad Eq. S42$$

where E_{Start} is the initial potential and v is the scan rate.

For CP measurements, where a fixed current density (j_{App}) is applied, the potential input for the Butler-Volmer equations is unknown and must be determined self-consistently. To achieve this a global differential equation is introduced to couple the electrode potential (E) to the applied current density j_{App}. The constraint ensures that for this 1D model the sum of the faradaic and capacitive charging currents, is equal to j_{App}.

$$0 = (j_{\mathrm{F}} + j_{\mathrm{C}}) - j_{\mathrm{App}}(t) \qquad Eq. S43$$

Here, the unknown variable, the applied electrode potential (E), is included in both the Butler-Volmer equations (***Eqs.*** **S27** and **S30**) used to determine j_{F} and the capacitive charging current, j_{C}:

$$j_{\mathrm{C}} = C_{\mathrm{dl}} \frac{dE}{dt} \qquad Eq. S44$$

where C_{dl} is the double layer capacitance. Including the capacitive charging is important for

ensuring the numerical stability and when neglected, the model did not solve at high current densities. A value of 20 μF/cm^2 is used for all the CP simulations, however increasing the value of the capacitance up to 100 μF/cm^2 to reflect the range of capacitance values measured for gold electrodes[7], made no significant difference to the pH profiles within the film.

Computational Software and Hardware

All of the simulations were performed using the *COMSOL Multiphysics version 6.2* with the transport of dilute species interface. Simulations were run on a computer with an *Intel(R) Xeon(R) Gold 5122 CPU* (3.60 GHz) and 256 GB RAM. To run a LSV over a 1 V range the simulations took ~ 2-3 minutes to solve. Running the CP simulation took ~ 20-30 seconds to generate 60 data points (one point every 2 s in a 120 s experiment). For more details of the simulations, including the numerical solver, the *COMSOL* Model report is provided.

Parameter Tables

The goal of the model is to replicate the experimental trends rather than provide quantitative agreement. This approach was chosen given the large number of parameters listed in **Table S3**, many of which are either estimated or taken from the literature and are not independent of each other.

Table S3: Model Parameters not previously defined in table 1 or 2.

Parameter	Value	Comments and references
d_{film}	1.6 or 1.1 μm	Measured by CLSM
$d_{\mathrm{film,AFM}}$	0.5 μm	Measured by AFM
L	1 cm	-
k^0_{PR}	10^{-6} m s^{-1}	Estimated from experiments on a bare gold electrode and increased to 10^{-4} m s^{-1} for the polymer-coated case
α_{PR}	0.5	-

k^0_{WR}	10^{-18} m s^{-1}	Estimated from experiments
α_{WR}	0.5	-
T	298.15 K	-
E^0	0 V *vs.* RHE	-
k_1	3.46×10^8 s^{-1}	-
k_{-1}	5×10^{10} M^{-1} s^{-1}	-
$K_{a,1}$	2.16	Taken from ref[8]
k_2	3083 s^{-1}	-
k_{-2}	5×10^{10} M^{-1} s^{-1}	-
$pK_{a,2}$	7.21	Taken from ref[8]
k_3	0.0239 s^{-1}	-
k_{-3}	5×10^{10} M^{-1} s^{-1}	-
$pK_{a,3}$	12.32	Taken from ref[8]
k_4	2.5×10^{-5} s^{-1}	Taken from refs[4,9,10]
k_{-4}	1.4×10^{11} M^{-1} s^{-1}	
pK_4	14.00	Also known as pK_w, and is calculated from k_4 and k_{-4}
$c_{\mathrm{Amine,tot}}$	3.8 M	Determined by dividing the amount of amines in the drop coated polymer by the film volume.
k_5	0.1 s^{-1}	-
k_{-5}	10^{11} M^{-1} s^{-1}	Estimated
$pK_{a,5}$	11	Estimated pK_a for the primary amines based on refs[5,6]
k_6	1000 s^{-1}	-
k_{-6}	10^{11} M^{-1} s^{-1}	Estimated
$pK_{a,6}$	7	Estimated pK_a for the secondary amines based on refs[5,6]
k_7	10^5 s^{-1}	-
k_{-7}	10^{11} M^{-1} s^{-1}	Estimated
$pK_{a,7}$	5	Estimated pK_a for the tertiary amines based on refs[5,6]
$D_{\mathrm{H^+}}$	9.3×10^{-9} m^2 s^{-1}	Taken from ref[8]
$D_{\mathrm{OH^-}}$	5.3×10^{-9} m^2 s^{-1}	
$D_{\mathrm{H_3PO_4}}$	9.6×10^{-10} m^2 s^{-1}	Estimated
$D_{\mathrm{H_2PO_4^-}}$	9.6×10^{-10} m^2 s^{-1}	Taken from ref[8]
$D_{\mathrm{HPO_4^{2-}}}$	7.6×10^{-10} m^2 s^{-1}	
$D_{\mathrm{PO_4^{3-}}}$	8.2×10^{-10} m^2 s^{-1}	
C_{dl}	20 μF cm^{-2}	Assumed based on ref[7]
ν	0.01 V/s	Taken from the experiments

Estimating the Electron Transfer Kinetics

The rate constants k^0_{PR} and k^0_{WR} were estimated to be 10^{-6} m s^{-1} and 10^{-18} m s^{-1} respectively during fitting to the LSVs giving a reasonable agreement between simulated and experimental LSV of the bare Au-electrodes (**Figure S19 A**). We see an earlier onset of the proton reduction wave in the presence of the film suggesting that there is change in the electron transfer kinetics, which we account for by increasing k^0_{PR} to 10^{-4} m s^{-1} in the polymer coated case.

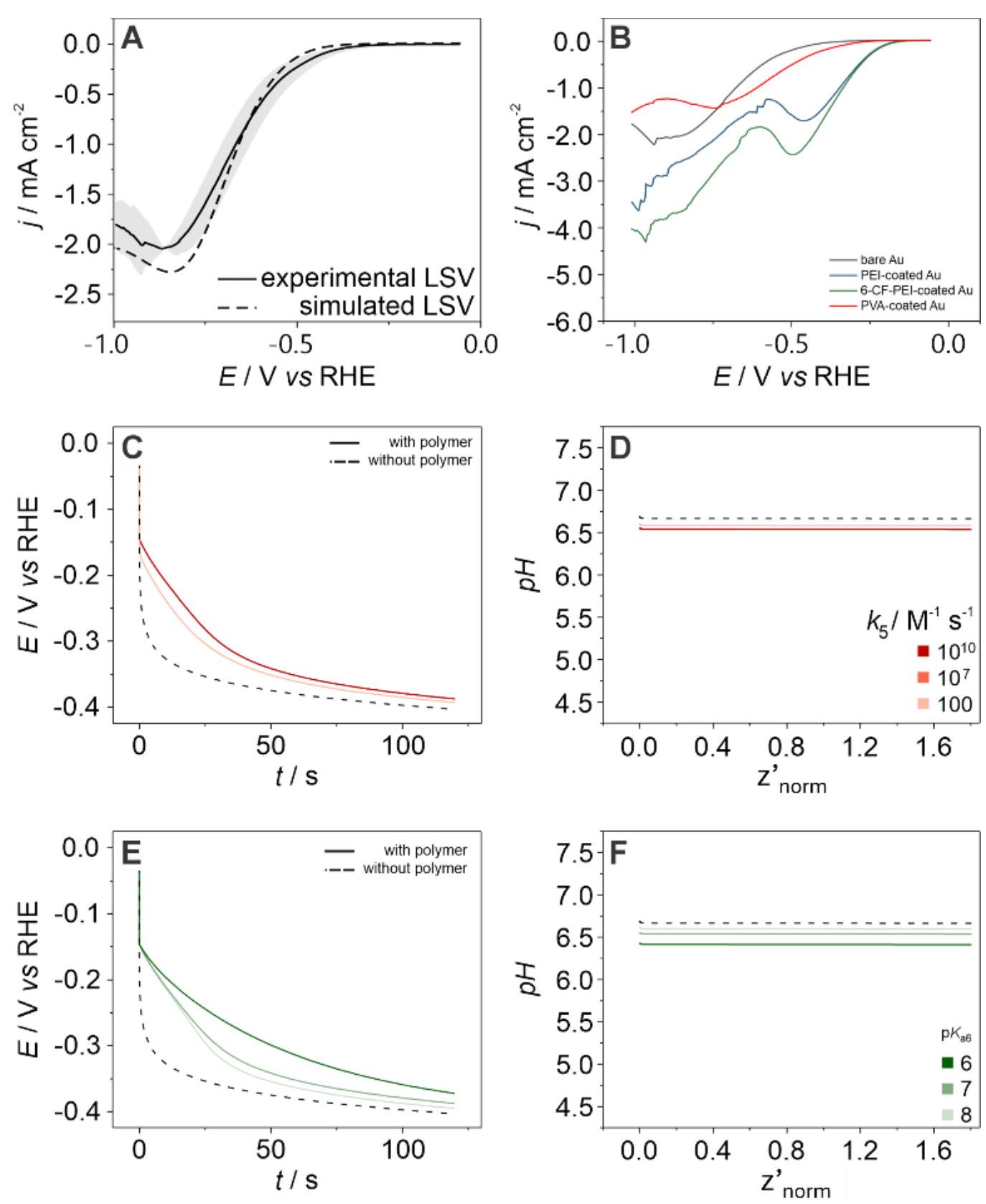

Figure S19: Insights by finite-element model. **A.** Comparison of experimental (black, solid line) and simulated LSVs (dotted line) of a static Au-disc electrode (A = 0.19 cm^2) at a scan rate of 10 mV s^{-1} in potassium phosphate buffer (c = 0.1 M, pH 6.2). Experimental data is presented as average value ± std. deviation (shaded area) from three individual electrodes. Adjusting $k^0_{PR} = 10^{-6}\ m\,s^{-1}$ for the bare gold electrodes and $k^0_{PR} = 10^{-4}\ m\,s^{-1}$ for the polymer-covered gold electrodes and $k^0_{WR} = 10^{-18}\ m\ s^{-1}$ for both cases lead to a reasonable fits. These values were applied for pH-simulations**. B.** LSVs (ν = 10 mV s^{-1}, A = 0.19 cm^2) of Au-RDEs without coating (black), with a PEI-coating (blue), with a 6-CF-PEI-coating (green) or with a PVA-coating (red) in the absence of rotation (ω = 0 rpm). **C**. Comparison of simulated CPs (j_{app} = -0.5 mA cm^{-2}) of PEI-coated electrodes (solid lines) and bare gold (dashed line) at different deprotonation rates k_6 of the secondary amines (see *Eq. S20*) as indicated in the legend with the corresponding pH distributions shown in panel **D**. The data sets of k_6 = 10^7 $M^{-1}s^{-1}$ and k_6 = 10^{10} $M^{-1}s^{-1}$ are fully overlapped. **E.** Simulated CPs (j_{app} = -0.5 mA cm^{-2}) at varied pK_a values of the secondary amines at a bulk pH of 4.5 with the corresponding pH distributions shown in panel **F**. Note that the dashed curves (bare gold electrodes) in panels **C** and **E** were simulated using a $k^0_{PR} = 10^{-4}\ m\ s^{-1}$ to solely capture effects induced by proton-transport.

Discussion on the Inhomogeneous Film Thickness

Figure S20 Figure S16 highlight the film's inherent inhomogeneity in terms of thickness. The lower thickness determined by liquid AFM as compared to that determined by CLSM may be caused by the lower spatial resolution of optical microscopy (± 0.3 µm). The estimation of the amine concentration in the film is thickness dependent. The highest determined film thickness of 1.6 µm corresponds to an amine concentration of 3.8 M, whereas the lowest possible thickness corresponds to a value of 9 M. The temporal pH changes, as well as the pH profiles were simulated for both cases showing identical trends with only marginal changes in the absolute pH-values (**Figure S20**).

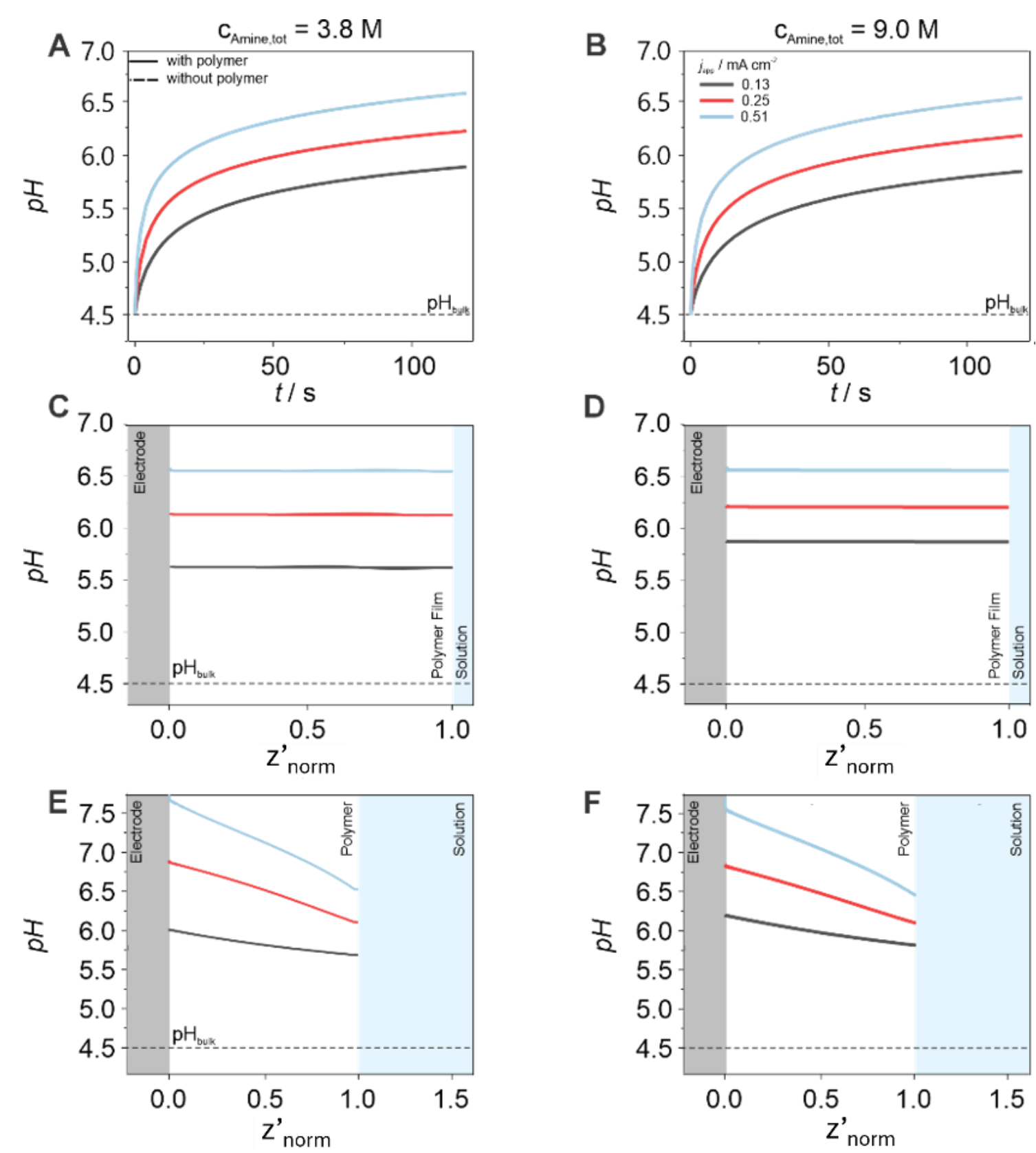

Figure S20: Influence of the amine concentration in the film. Temporal development of the operando pH values in the film (**A**, **B**) and spatial pH distributions throughout the film (**C** - **F**). Data in panels **A**, **C** and **E** is simulated based on an amine concentration $c_{amine,tot}$ = 3.8 M. This data is shown in main text **Figures 2 F**, **3 B** and **3 D**, respectively and is replotted here for direct comparison. Panels **B**, **D** and **F** show simulations based on an increased amine concentration of $c_{amine,tot}$ = 9.0 M leaving all other parameters unchanged. The higher concentration stems from the lower film thicknesses determined by AFM (**Figure S16**).

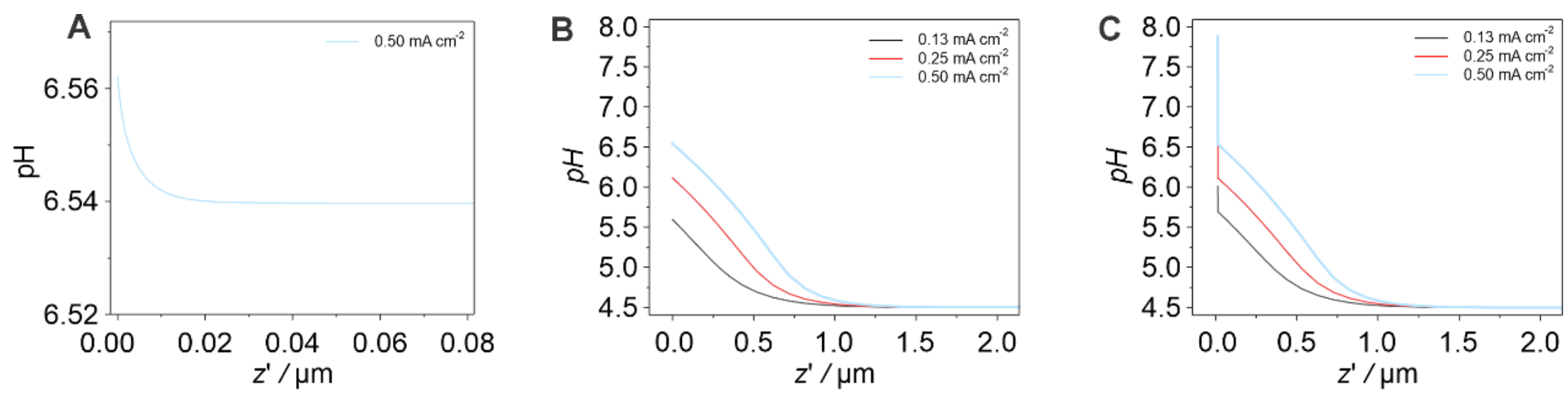

Figure S 21: Details on concentration profiles. A. Simulated pH close to the electrode surface (0 - 80 nm). The simulation was run at a current density of 0.5 mA cm^{-2}, a film thickness d = 1.6 and c_{amine} = 3.8 M assuming unrestricted diffusion of phosphate species. Note that this local pH increase cannot be resolved by optical microscopy. Extended views of the concentration profiles shown in **Figure 3 B** (**B**) and **3D** (**C**). At ~1.2 mm remote from the electrode surface pH_{bulk} = 4.5 was reached.

Supplementary Section 6 – Evaluation of Confocal Laser Scanning Microscopy

Calibration of the CLSM Emission Intensities

Calibration to the corresponding pH values was realized by collecting numerous z-scans through the polymer-films using an inverted fluorescence microscope (*Nikon Eclipse Ti*) operated with *NIS-Elements AR 5.11.02* software. Each z-scan is represented by a stack of images that were exported as nd2-files. These are provided as Supplementary Data. Several positions were initially scanned at pH 4.5 and the x-y positions were stored in the software. These scans are called "reference data" in the following. After mapping a sufficient amount of positions at pH 4.5, the buffer was changed to a different pH value as described in the experimental section. The sample was moved to a previously scanned position and a second z-scan was recorded. These subsequent scans recorded at pH-values other than pH 4.5 are called "target data" in the following. The mean intensities of the individual images of the z-stacks were extracted using a self-made script in *Matlab 2022b,* which is provided as Supplementary Data. nd2 files were imported using the *bfGetReader* function from the Bio-Formats *Matlab* package (*bfmatlab*).[12] Within the *Matlab* script the mean intensities were extracted and background corrected. The background intensity was extracted from images at polymer-free positions and was found to be independent of position and pH (**Figure S22 A** and **B)**. The background corrected reference data was fitted to a Gauss

function and the full width half maximum (FWHM) was determined. Afterwards, the target data was fitted by a Gauss function fixing the FWHM to the same value as that observed for the reference scan, because the FWHM criterion defines the dimensions of the film in CLSM. This approach is justified, because we did not observe any pH-induced polymer swelling and shrinking, neither by CLSM (**Figure S22 C**) nor by liquid AFM (**Figure S24**). The Gauss function of the target pH was aligned with that of the reference at pH 4.5 (**Figure S22 D**). Normalization resulted in a calibration map that is flat in z-direction (**Figure S22 F**), therefore being represented by a 2 D logistic function (**Figure S22 G**). This function was used as calibration curve for local pH readout in CLSM. The robustness of this calibration approach was confirmed by normalizing the non-aligned intensity of the targets measurements revealing that calibration is accurate at low pH values, with some error at high pH (≥ 7.6) (**Figure S22 E**). Therefore, low current densities were chosen to avoid high pH-values.

***Operando* Experiments**

A position of the film that was not previously used for calibration was identified and a z-scan was recorded at the initial pH 4.5 at OCP (reference data). A chronopotentiometry was performed for 2.5 minutes to induce a local pH-change. After 2 minutes a z-scan was recorded to visualize the intensity distribution *in-operando* (target data). This duration was chosen, because the widefield microscopy images suggested the build-up of a steady state after 2 minutes. Extracting the mean intensities of the target data, by the aforementioned *Matlab 2022b* script and plotting the intensity of each slice against the z-position may lead to aligned Gauss distributions (**Figure S23 D, G, J**) or to Gauss distributions whose maxima are shifted towards the electrode/film interface (**Figure S23 E, H, K**). The z-dependent intensities were normalized by the Gauss-function of the reference

measurement. The logistic calibration function allowed to recalculate these intensity ratios into pH. Recalculation was performed in Microsoft Excel and the corresponding file is provided as Supporting Data. Positions, where the Gauss functions of the target and the reference data are aligned, correspond to homogeneous pH distributions shown in main text **Figure 3 A**. Positions, where the Gauss functions of the target is shifted with respect to the reference data, correspond to the pH profiles shown in main text **Figure 3 C**. Generally, the former case is observed at positions with a thick, porous film and the latter at positions with a thin compact film.

Further Discussion on the Method.

As the intensity shift towards the electrode must be strictly pH-induced, this method is strongly dependent on the absence of a focus drift. Therefore, a *Nikon Perfect Focus System* (PFS) was applied to correct for eventual focus drifts. Additionally, a set of control experiments was performed without the PFS to show that marginal focus drifts do not significantly alter the observed pH profiles (**Figure S25**).

CLSM forces a high light intensity towards a small focus point. Therefore, strong photobleaching occurred even at the lowest possible laser intensities (compare black and gray traces in **Figure S23**). Bleaching may be accelerated by the complex chemical environment inside the polymer offering potential reaction partners for the excited fluorophore. Nevertheless, the measuring procedure ensures that both, positions for calibration and positions for *operando* measurements had been exposed to the same bleaching history (*i.e.* one "reference scan" followed by one "target scan"). Hence, bleaching effects are covered by the calibration procedure.

Effects of metal-surface induced quenching are expected to be negligible,[13] because it is limited to distances at which electron transfer can take place (~14 Å)[14] which is two orders of magnitude

lower than the microscope resolution.[15] Additionally, these quenching effects were reported to be most pronounced at rough surfaces. Our Au-layers, instead, are very flat, with roughness features not exceeding the low nanometer range (see polymer free region of the AFM scans in **Figure S24**).

Recording z-scans at a potential of 0 V *vs.* RHE showed no increase in emission intensity as compared to OCP (**Figure S26**). At these potentials, the Au-surface is negatively charged, but no hydrogen is evolved. This confirmed that not the charging of the Au-surface, but the change in local pH is responsible for the intensity changes.

A contribution of undesired convectional effects to the pH-profiles is unlikely, because the working electrode is arranged horizontally, mitigating natural convection.[16] Although the solution is overflown by N_2 to avoid contribution of ORR, the high distance and the moderate flow rate should not induce significant convection. The measuring cell is positioned on top of an inverted microscope, which is optimized to minimum vibration for high resolution image acquisition. Thus, convection induced by vibration of the instrument is very unlikely.

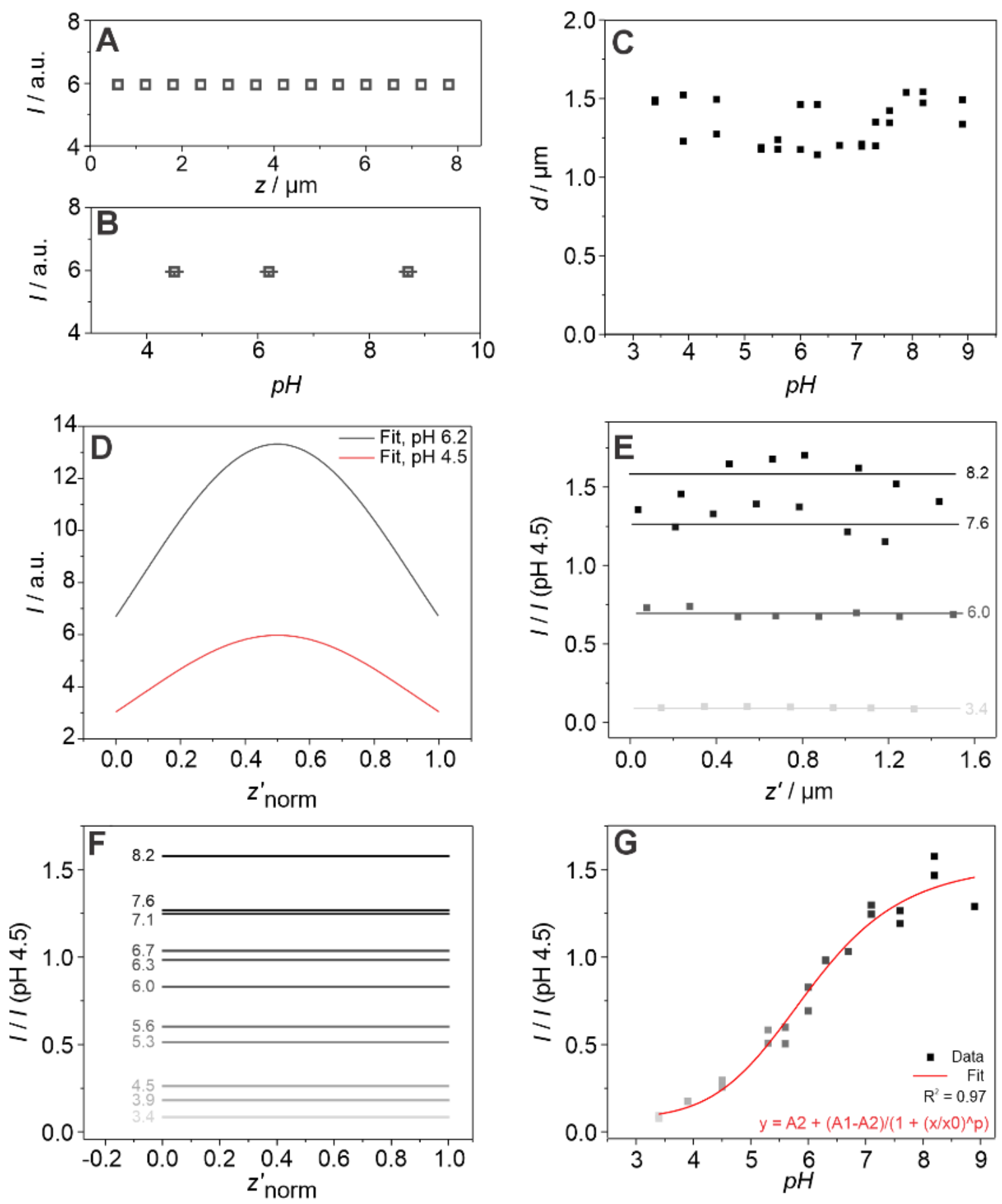

Figure S22. Calibration details of confocal measurements. Background intensities (I_{ex} = 1 %) of a polymer-free electrode spot *vs*. *z*-position (**A**) and *vs*. pH (**B**). **C**. Thicknesses of the films at various pH values. **D**. Intensity region >1/2 I_{max} *vs*. normalized film thickness z'_{norm} extracted from the same position. The graph is exemplary showing pH 6.2 (black) with pH 4.5 (red) as reference for normalization. The fit of intensity distribution at pH 6.2 is fixed at the same FWHM and shifted to the same center position. **E**. Intensity ratios calculated by normalizing the Gaussian fit at a given pH value (e.g. the red curve in panel **D**) by the reference curve (e.g. the black curve in panel **D**) (solid lines). As a quality check, the non-fitted data points were divided by each other (filled squares). **F**. Full set of normalized intensity ratios at different pH values. **G**. Calibration curve by extracting the data points from panel **F** (black squares) fitted to a logistic function (red curve) with the applied equation and accuracy depicted in the panel.

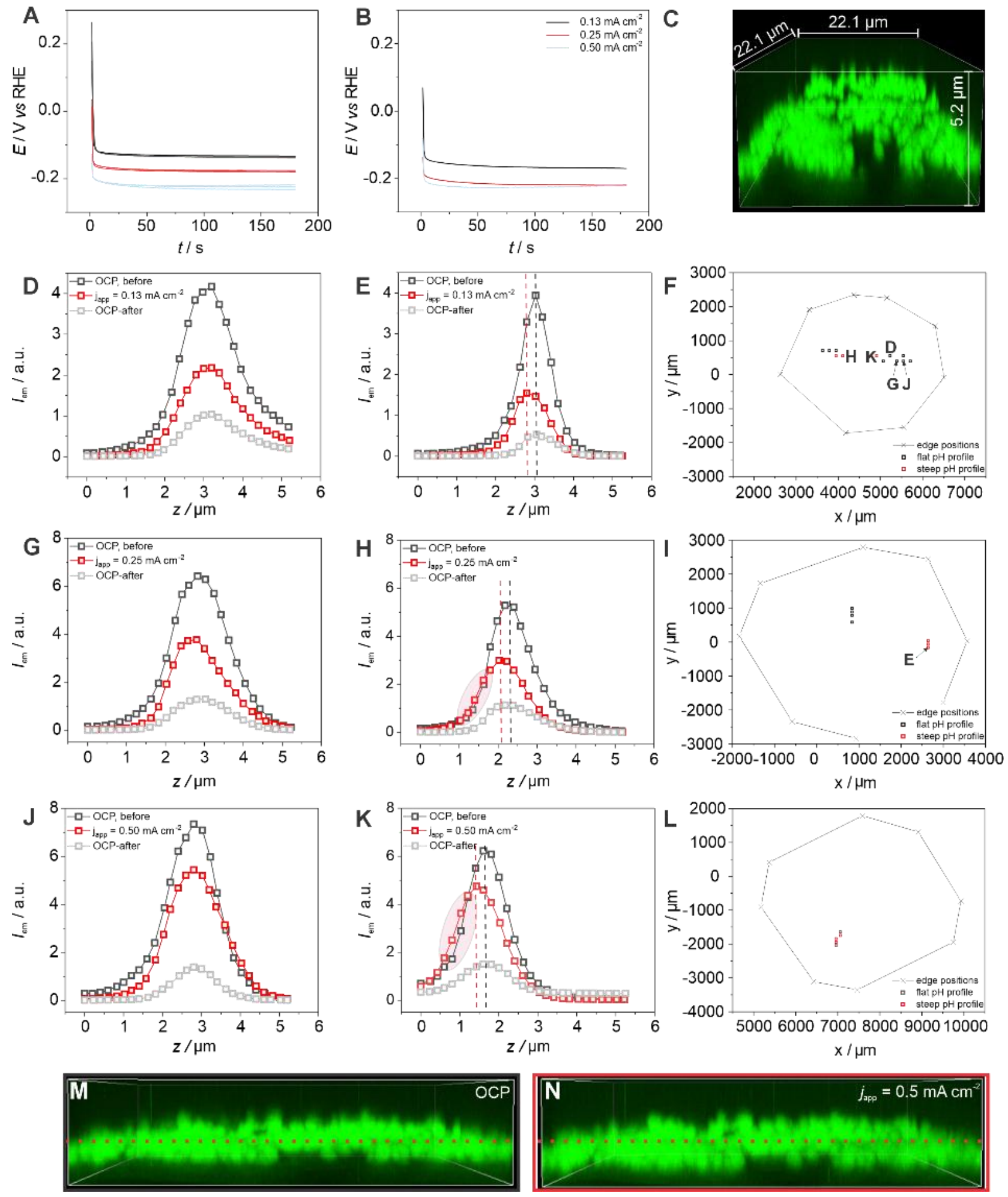

Figure S23: Details on *operando* EC-CLSM measurements. Chronopotentiometric transients (j_{app} as indicated) at Au-electrodes functionalized with a film of 6-CF-PEI ($A = 0.19$ cm^2) corresponding (**A**) to the pH-distributions shown in **Figure 3 A** and (**B**) to that in **Figure 3 C**. The fact that different chronopotentiometric transients can be observed from the same film is most likely due to film aging and possible resistance differences, when the cell is reconnected. **C**. Exemplary 3D visualization of the fluorescence inside the film at OCP including the dimensions of the scanned region of interest. Intensity distributions **D**, **G** and **J** correspond to the flat pH-measurements in **Figure 3 A**, while **E**, **H**, **K** correspond to the pH profiles in **Figure 3 C**. Panels **M** and **N** show the frontal view of the same position at OCP (**M**) and at an applied current (**N**). The red dotted lines were added at the same *z*-position to visualize the shift of intensity towards the electrode surface. Plotting the intensities of **M** and **N** *vs. z*-position delivers the intensity distribution in panel **K**. The other intensity distributions **D**, **E**, **G**, **H**, **J** recorded in other films or at different positions were obtained accordingly. The initial z-scan at OCP (reference data) is shown in black, the z-scan under an applied potential (target data) in red and another OCP recorded at the same position 20 minutes after the measuring sequence is shown in gray. For homogeneous pH-distributions, the Gauss-curves in the presence and the absence of an applied potential are aligned. For-pH profiles the maxima of the intensity distributions shift towards the electrode surface as indicated by the black and red dashed lines. Regions of a strongly increased intensity ratio (synonymous to an increased local pH) are highlighted by the red ellipsoids. The precise alignment of the intensity distributions before (black) and after the measurement (gray) exclude the possibility of a focus drift confirming that these effects are solely pH-induced. The intensity decrease over the scans shows an intense contribution of photobleaching, which does not affect pH-readout, as it is considered in the calibration procedure. Flat and steep pH profiles were found at different x-y-positions of three individual films. **F**, **I** and **L** show the x-y coordinates of the individual polymer film. 8 edge positions (marked by the crosses) were identified and interpolated by lines to visualize the film dimensions. 22.1 x 22.1 x 5.2 µm regions-of-interest were scanned for pH determination (black and red squares). Black squares mark positions with a flat pH profile, whereas red squares mark positions with curved pH profiles. The representative positions used for the intensity profiles in panels **D**, **E**, **G**, **H**, **J** and **K** are labeled with the respective letter.

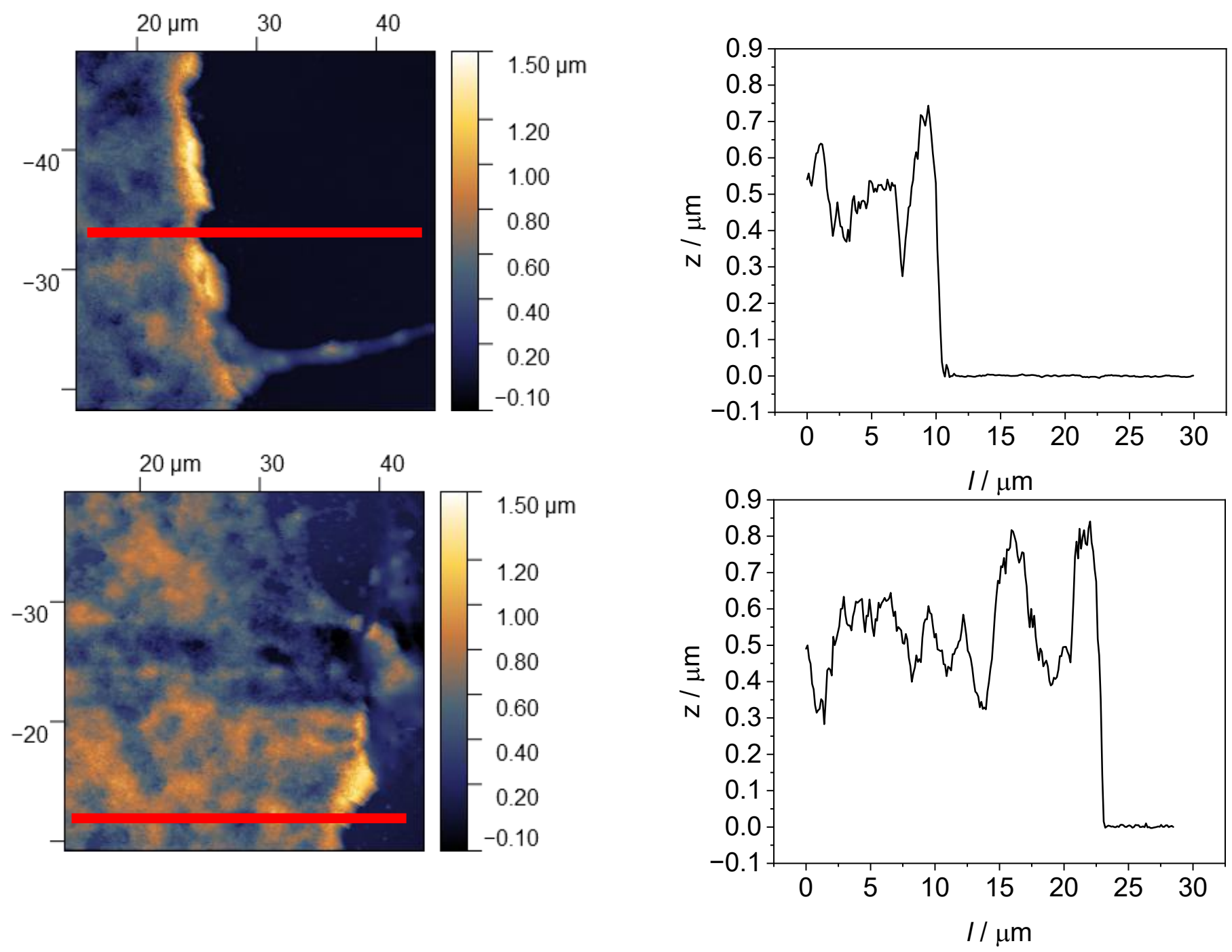

Figure S24. Absence of swelling by liquid AFM. AFM-images with the corresponding height profile determined along the red line recorded in KH_2PO_4-solution (0.1 M, pH 4.5) (upper panels) and in potassium phosphate buffer (0.1 M, pH 7.5) (lower panels).

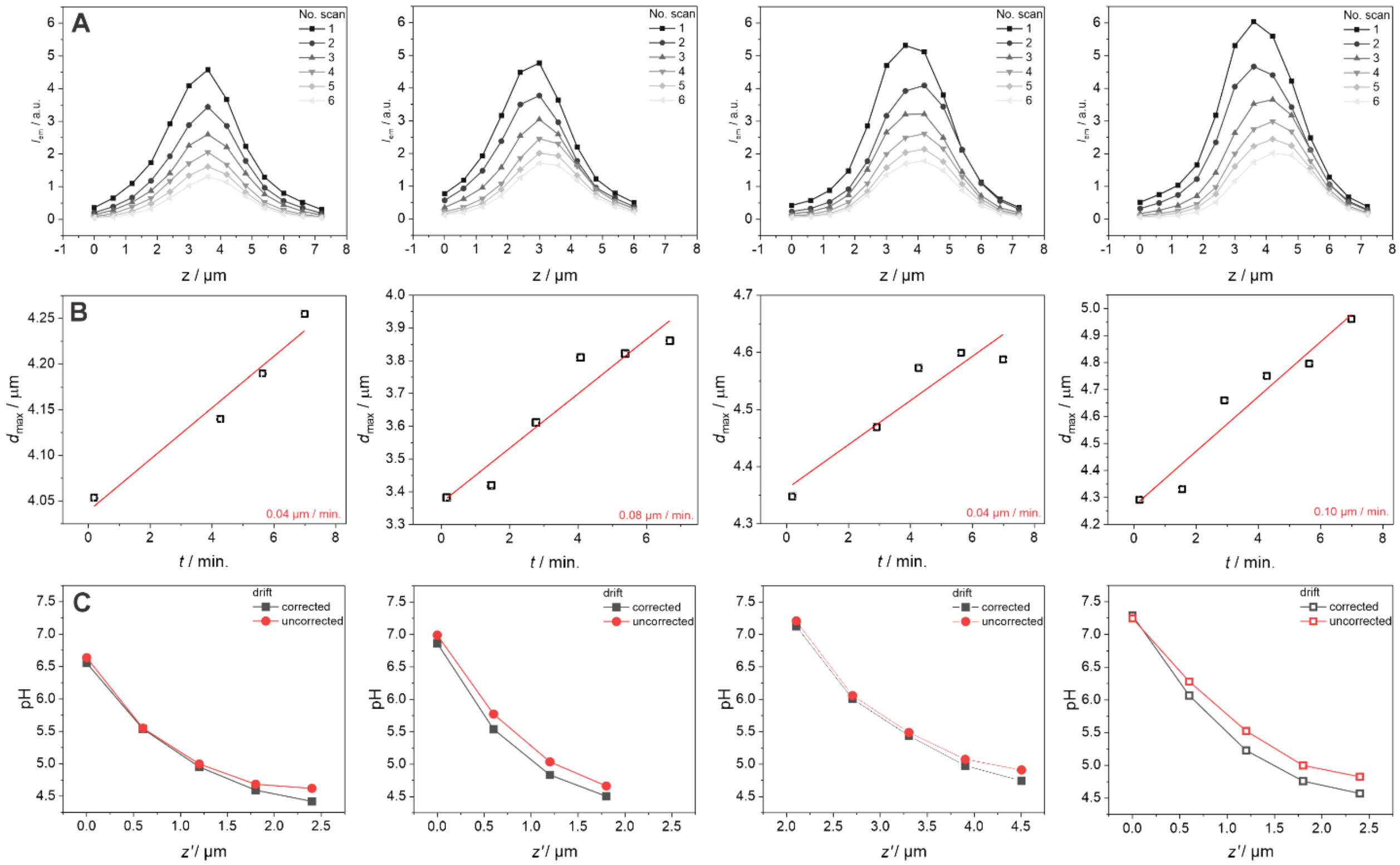

Figure S25: Marginal effect by possible focus drift. A. Consecutive z-scans showing a small shift in the position of the location of maximum intensity. **B**. Positions of the intensity maxima were extracted from panels **A** and plotted against time (black squares). The points were fitted by a linear regression and the slope (value given in red at the lower right corner of the panels) was interpreted as an estimate of the temporal focus drift. **C**. Concentration profiles were recorded plotted with (black points) and without (red points) correcting for the focus drift extracted in panels **B**. The effect was small with a maximum deviation of 0.26 pH units.

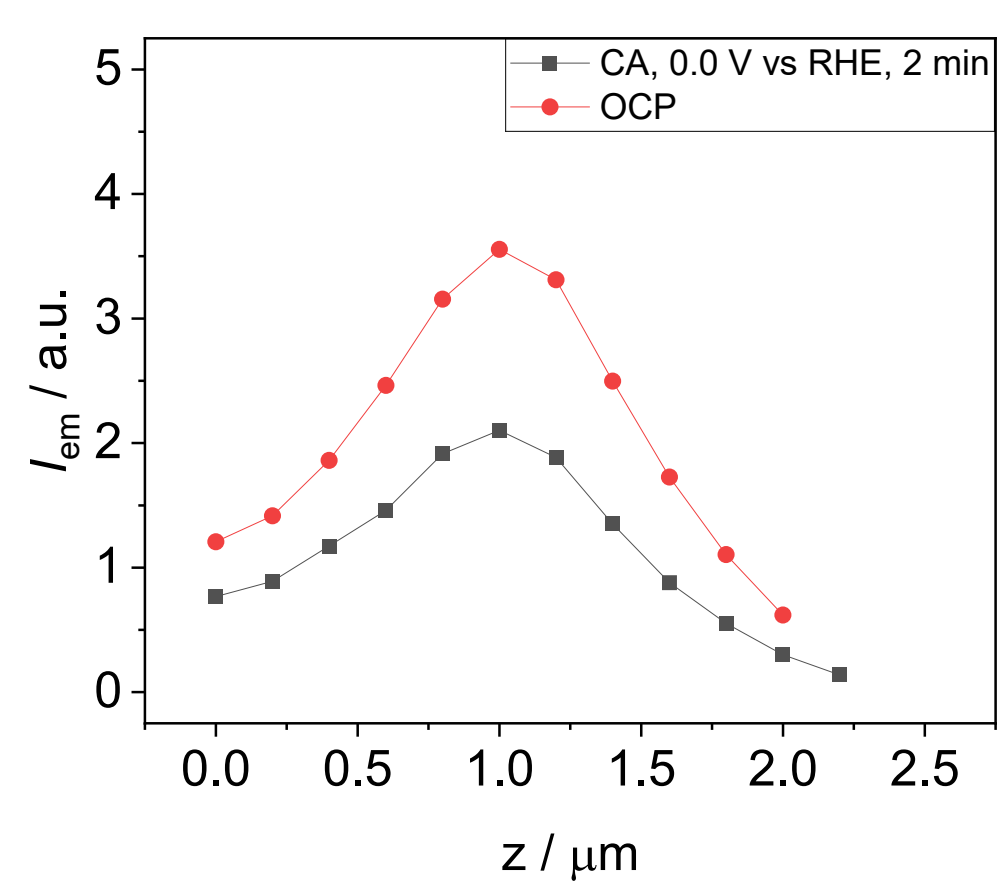

Figure S26: No intensity increase upon Au-charging. Mean intensities *vs*. focus position of z-scans recorded at OCP (red) and at E_{app} = 0 V (black, left panel) *vs*. RHE.

References

(1) Xu, W.; Park, J. Y.; Kattel, K.; Ahmad, Md. W.; Bony, B. A.; Heo, W. C.; Jin, S.; Park, J. W.; Chang, Y.; Kim, T. J.; Park, J. A.; Do, J. Y.; Chae, K. S.; Lee, G. H. Fluorescein-Polyethyleneimine Coated Gadolinium Oxide Nanoparticles as T1 Magnetic Resonance Imaging (MRI)–Cell Labeling (CL) Dual Agents. *RSC Adv.* **2012**, *2* (29), 10907. https://doi.org/10.1039/c2ra21052e.

(2) Case, D. A.; Dyson, H. J.; Wright, P. E. Use of Chemical Shifts and Coupling Constants in Nuclear Magnetic Resonance Structural Studies on Peptides and Proteins. In *Methods in Enzymology*; Elsevier, 1994; Vol. 239, pp 392–416. https://doi.org/10.1016/S0076-6879(94)39015-0.

(3) Yin, F.; Hu, Y.; Lu, S.; Yang, X.; Song, C.; Liu, H. A Novel Electrochemical Method Determining pKa and Transport Parameters of Buffers Using Acid-Base Transport Limitation Response. *Electrochimica Acta* **2023**, *471*, 143373. https://doi.org/10.1016/j.electacta.2023.143373.

(4) Eigen, M. Proton Transfer, Acid-Base Catalysis, and Enzymatic Hydrolysis. Part I: ELEMENTARY PROCESSES. *Angew. Chem. Int. Ed.* **1964**, *3* (1), 1–19. https://doi.org/10.1002/anie.196400011.

(5) Kokufuta, E. Colloid Titration Behavior of Poly(Ethyleneimine). *Macromolecules* **1979**, *12* (2), 350–351. https://doi.org/10.1021/ma60068a043.

(6) Willner, I.; Eichen, Y.; Frank, A. J.; Fox, M. A. Photoinduced Electron-Transfer Processes Using Organized Redox-Functionalized Bipyridinium-Polyethylenimine-Titania Colloids and Particulate Assemblies. *J. Phys. Chem.* **1993**, *97* (28), 7264–7271. https://doi.org/10.1021/j100130a024.

(7) Janek, R. P.; Fawcett, W. R.; Ulman, A. Impedance Spectroscopy of Self-Assembled Monolayers on Au(111): Evidence for Complex Double-Layer Structure in Aqueous $NaClO_4$ at the Potential of Zero Charge. *J. Phys. Chem. B* **1997**, *101* (42), 8550–8558. https://doi.org/10.1021/jp971698e.

(8) *CRC Handbook of Chemistry and Physics: A Ready-Reference Book of Chemical and Physical Data*, 97th edition.; Haynes, W. M., Ed.; CRC Press: Boca Raton London New York, 2017.

(9) Eigen, M.; De Maeyer, L. Self-Dissociation and Protonic Charge Transport in Water And. *Proc. R. Soc. Lond. Ser. Math. Phys. Sci.* **1958**, *247* (1251), 505–533. https://doi.org/10.1098/rspa.1958.0208.

(10) Eigen, M.; De Maeyer, L. Untersuchungen Über Die Kinetik Der Neutralisation. I. *Z. Für Elektrochem. Berichte Bunsenges. Für Phys. Chem.* **1955**, *59* (10), 986–993. https://doi.org/10.1002/bbpc.19550591020.

(11) Gallops, C. E.; Yu, C.; Ziebarth, J. D.; Wang, Y. Effect of the Protonation Level and Ionic Strength on the Structure of Linear Polyethyleneimine. *ACS Omega* **2019**, *4* (4), 7255–7264. https://doi.org/10.1021/acsomega.9b00066.

(12) Linkert, M.; Rueden, C. T.; Allan, C.; Burel, J.-M.; Moore, W.; Patterson, A.; Loranger, B.; Moore, J.; Neves, C.; MacDonald, D.; Tarkowska, A.; Sticco, C.; Hill, E.; Rossner, M.; Eliceiri, K. W.; Swedlow, J. R. Metadata Matters: Access to Image Data in the Real World. *J. Cell Biol.* **2010**, *189* (5), 777–782. https://doi.org/10.1083/jcb.201004104.

(13) Pineda, A. C.; Ronis, D. Fluorescence Quenching in Molecules near Rough Metal Surfaces. *J. Chem. Phys.* **1985**, *83* (10), 5330–5337. https://doi.org/10.1063/1.449695.

(14) Marcus, R. A.; Sutin, N. Electron Transfers in Chemistry and Biology. *Biochim. Biophys. Acta BBA - Rev. Bioenerg.* **1985**, *811* (3), 265–322. https://doi.org/10.1016/0304-4173(85)90014-X.

(15) Bouffier, L.; Doneux, T. Coupling Electrochemistry with in Situ Fluorescence (Confocal) Microscopy. *Curr. Opin. Electrochem.* **2017**, *6* (1), 31–37. https://doi.org/10.1016/j.coelec.2017.06.015.

(16) Janotta, B.; Schalenbach, M.; Turiaux, M.; Tempel, H.; Eichel, R. A. Buffering Effects of Supporting Electrolytes on pH Profiles in Electrochemical Cells. *Sci. Rep.* **2025**, *15* (1), 32458. https://doi.org/10.1038/s41598-025-18219-z.
