# Supplementary material for: Operando Electrochemical Fluorescence Microscopy Highlights Local Buffering and Mass Transport Effects in Polymer-Promoted Electrocatalysis
Source: J Am Chem Soc. 2026 Oct 6;148(41):44300–9. doi: 10.1021/jacs.6c11297 (PMC13693391; doi:10.1021/jacs.6c11297)

COMSOL MULTIPHYSICS®

260525 HER Polymer Film Model FINAL

Report date	May 25, 2026, 4:18:28 PM

Contents

1. Global Definitions 3
1.1. Parameters 3
1.2. Shared Properties 7
2. Component 1 8
2.1. Definitions 8
2.2. Geometry 1 19
2.3. HER 20
2.4. Phosphate 30
2.5. Polymer Film 37
2.6. Events 43
2.7. Global ODEs and DAEs 45
2.8. Mesh 1 46
3. Study 1 - LSV - Potential Sweep 49
3.1. Parametric Sweep - LSV 49
3.2. Time Dependent 49
3.3. Solver Configurations 50
4. Study 2 - CA - Constant Potential 56
4.1. Parametric Sweep 56
4.2. Time Dependent 56
4.3. Solver Configurations 57
5. Study 3 - CP - Constant Current 64
5.1. Parametric Sweep 64
5.2. Time Dependent 65
5.3. Solver Configurations 65
6. Results 73
6.1. Datasets ..... **Error! Bookmark not defined.**
6.2. Derived Values ..... **Error! Bookmark not defined.**
6.3. Tables ..... **Error! Bookmark not defined.**
6.4. Plot Groups 73
6.5. Evaluation Groups ..... **Error! Bookmark not defined.**

1 Global Definitions

Date	May 25, 2026, 3:12:12 PM

GLOBAL SETTINGS

Name	260525 HER Polymer Film Model FINAL.mph
Version	COMSOL Multiphysics 6.4 (Build: 343)

USED PRODUCTS

COMSOL Multiphysics
Chemical Reaction Engineering Module

COMPUTER INFORMATION

CPU	Intel64 Family 6 Model 85 Stepping 4, 2 sockets, 8 cores, 254.63 GB RAM
Operating system	Windows 10

1.1 PARAMETERS

1.1.1 1 - General

1 - GENERAL

Name	Expression	Value	Description
E_ref	0 [V]	0 V	Reference Electrode - SHE
pH_b	4.5	4.5	Bulk pH of the Buffer
f	(F_const/(R_const*T))	38.922 1/V	Collection of constants
T	298.15 [K]	298.15 K	Temperature
d_film	1.6 [um]	1.6E−6 m	Thickness of polymer layer
cH_b	10^(-pH_b) [M]	0.031623 mol/m^3	Calculated bulk concentration of H+
c0	1 [mol/L]	1000 mol/m^3	Constant for unit conversion (1 M)
cP_tot	0.1[M]	100 mol/m^3	Total concentration of phosphate species in the buffer electrolyte

1.1.2 2 - Diffusion Coefficients

2 - DIFFUSION COEFFIENTS

Name	Expression	Value	Description
D_H_proton	9.3E-9 [m^2/s]	9.3E−9 m^2/s	Diffusion coefficient of H+ (CRC 97th Ed.)

Name	Expression	Value	Description
D_H2	5.5E-9 [m^2/s]	5.5E−9 m^2/s	Diffusion coefficient of H2 (CRC) or 4.4E-9 (ref J Phys. Chem. C)
D_H2PO4	9.6E-10 [m^2/s]	9.6E−10 m^2/s	Diffusion coefficient of H2PO4- (CRC 97th Ed.)
D_HPO4	7.6E-10 [m^2/s]	7.6E−10 m^2/s	Diffusion coefficient of HPO42- (CRC 97th Ed.)
D_OH	5.3E-9 [m^2/s]	5.3E−9 m^2/s	Diffusion coefficient of OH- (CRC 97th Ed.)
D_PO4	8.2E-10 [m^2/s]	8.2E−10 m^2/s	Diffusion coefficient of PO43- (CRC 97th Ed.)
D_H2O	2.2E-9 [m^2/s]	2.2E−9 m^2/s	Diffusion coefficient of water (CRC 97th Ed.)
D_H3PO4	9.6E-10 [m^2/s]	9.6E−10 m^2/s	Estimated
Film_Phosphate_Scale	1	1	Used to reduce the diffusion coefficient of all 4 phosphate species by the same scaling
Film_H_OH_Scale	1	1	Used to reduce the diffusion coefficient of all H+ and OH- species by the same scaling

1.1.3 3 - Electron Transfer

3 - ELECTRON TRANSFER

Name	Expression	Value	Description
alpha_PR	0.5	0.5	
k_app1	1E-4 [cm/s]	1E−6 m/s	Electron transfer coefficient for proton reduction
k_app2	1E-16 [cm/s]	1E−18 m/s	Electron transfer coefficient for water reduction
alpha_WR	0.5	0.5	

1.1.4 4A - Water Autoionization

4A - WATER AUTOIONISATION

Name	Expression	Value	Description
cH2O_b	55.5 [M]	55500 mol/m^3	Bulk concentration of water
cOH_b	max((Kw)/(cH_b/ 1[M]), eps) [M]	3.134E−7 mol/m^3	Calculated bulk concentration of OH-
k_D	2.5E-5 [s^ - 1]	2.5E−5 1/s	Dissociation rate constant water
k_R	1.4E11 [M^ - 1 *s^ - 1]	1.4E8 $m^3/(s \cdot mol)$	Rate constant for water association

Name	Expression	Value	Description
Kw	((k_D/k_R)*cH2O_b / 1[M^2])	9.9107E−15	
pKw	-log10(Kw)	14.004	

1.1.5 4B - Phosphate Buffer

4B - PHOSPHATE BUFFER

Name	Expression	Value	Description
k_b1	5E10 [M^ - 1*s^ - 1]	5E7 m^3/(s·mol)	Assumed to be in the range 10^10 - 10^11 [M^-1 s^-1]
k_b2	k_b1	5E7 m^3/(s·mol)	Assumed to be in the range 10^10 - 10^11 [M^-1 s^-1]
k_b3	k_b1	5E7 m^3/(s·mol)	Assumed to be in the range 10^10 - 10^11 [M^-1 s^-1]
k_f1	k_b1*(Ka1*1[M])	3.4592E8 1/s	
k_f2	k_b2*(Ka2*1[M])	3083 1/s	
k_f3	k_b3*(Ka3*1[M])	0.023932 1/s	
Ka1	10^(-pKa1)	0.0069183	
Ka2	10^(-pKa2)	6.166E−8	
Ka3	10^(-pKa3)	4.7863E−13	
pKa1	2.16	2.16	
pKa2	7.21	7.21	
pKa3	12.32	12.32	

1.1.6 4C -PEI Polymer Film Parameters

4C -PEI POLYMER FILM PARAMATERS

Name	Expression	Value	Description
pKa7	5	5	pKa of the tertiary amine
pKa6	7	7	pKa of the secondary amine
pKa5	11	11	pKa of the primary amine
Ka7	10^(-pKa7)	1E−5	
Ka6	10^(-pKa6)	1E−7	
Ka5	10^(-pKa5)	1E−11	
k_7f	k_5b*Ka7*c0	1E6 1/s	
k_7b	k_5b	1E8 m^3/(s·mol)	
k_6f	k_5b*Ka6*c0	10000 1/s	
k_6b	k_5b	1E8 m^3/(s·mol)	
k_5f	k_5b*Ka5*c0	1 1/s	

Name	Expression	Value	Description
k_5b	1E11 [M^ - 1 *s^ - 1]	1E8 $m^3/(s \cdot mol)$	
cAmine_tot	3.8 [M]	3800 mol/m^3	Total concentration of amine groups
alpha_k3	1/(1 + 10^(pH_b - pKa7))	0.75975	Used for calculating the initial concentrations of the polymer groups
alpha_k2	1/(1 + 10^(pH_b - pKa6))	0.99685	Used for calculating the initial concentrations of the polymer groups
alpha_k1	1/(1 + 10^(pH_b - pKa5))	1	Used for calculating the initial concentrations of the polymer groups

1.1.7 5 - Waveform Potential + Current

5 - WAVEFORM POTENTIAL + CURRENT

Name	Expression	Value	Description
Area	0.19 [cm^2]	1.9E−5 m^2	
Cdl	20 [μF/cm^2]	0.2 F/m^2	This is arbitrary but needed to stabilise the feedback loop
E_App	-0.4 [V]	−0.4 V	CA - Applied Potential
E_End	-1.0 [V]	−1 V	
E_Start	0 [V]	0 V	
E0	0 [V]	0 V	
j_pulse	-0.25[mA/cm^2]	−2.5 A/m^2	
j_start	0[A]	0 A	
Scan_Duration	abs(E_Start - E_End)/v	100 s	
Step	1 [mV]	0.001 V	
Step_time	Step/v	0.1 s	
v	0.010 [V/s]	0.01 V/s	

1.1.8 6 - Geometry + Mesh

6 - GEOMETRY + MESH

Name	Expression	Value	Description
L	1 [cm]	0.01 m	
sign	-1	−1	
Min_Element	0.05 [nm]	5E−11 m	

1.2 SHARED PROPERTIES

1.2.1 Default Model Inputs

Tag	cminpt

2 Component 1

SETTINGS

Description	Value
Unit system	Same as global system (SI)

2.1 DEFINITIONS

2.1.1 Variables

4A+B - Electrolyte Buffer Reactions + concentrations

SELECTION

Geometric entity level	Entire model

Name	Expression	Unit	Description
r_H3PO4	k_f1*(cH3PO4) - k_b1*(cH*cH2PO4)	mol/(m³·s)	
r_H2PO4	k_f2*(cH2PO4) - k_b2*(cH*cHPO4)	mol/(m³·s)	
r_HPO4	k_f3*(cHPO4) - k_b3*(cH*cPO4)	mol/(m³·s)	
r_water	k_D*cH2O_b - k_R*cH*cOH	mol/(m³·s)	
cH3PO4_b	(cP_tot*(cH_b/1[M])^3)/((cH_b/1[M])^3 + Ka1*(cH_b/1[M])^2 + Ka1*Ka2*(cH_b/1[M]) + Ka1*Ka2*Ka3)	mol/m³	
cH2PO4_b	(cP_tot*Ka1*(cH_b/1[M])^2)/((cH_b/1[M])^3 + Ka1*(cH_b/1[M])^2 + Ka1*Ka2*(cH_b/1[M]) + Ka1*Ka2*Ka3)	mol/m³	
cHPO4_b	(cP_tot*Ka1*Ka2*(cH_b/1[M]))/((cH_b/1[M])^3 + Ka1*(cH_b/1[M])^2 + Ka1*Ka2*(cH_b/1[M]) + Ka1*Ka2*Ka3)	mol/m³	
cPO4_b	(cP_tot*Ka1*Ka2*Ka3)/((cH_b/1[M])^3 + Ka1*(cH_b/1[M])^2 + Ka1*Ka2*(cH_b/1[M]) + Ka1*Ka2*Ka3)	mol/m³	

4C - Polymer Film Buffer Reactions + Initial Concentrations

SELECTION

Geometric entity level	Domain
Selection	Geometry geom1: Dimension 1: Domain 1

Name	Expression	Unit	Description
r_side_chain_primary	k_5f*cRNH3 - k_5b*cH*cRNH2	mol/(m³·s)	
r_side_chain_secondary	k_6f*cR2NH2 - k_6b*cH*cR2NH	mol/(m³·s)	
r_side_chain_tertiary	k_7f*cR3_NH - k_7b*cH*cR3_N	mol/(m³·s)	

Name	Expression	Unit	Description
cR3NH_p_in	(cAmine_tot/4)*alpha_k3	mol/m³	
cR3N_dp_in	(cAmine_tot/4)*(1 - alpha_k3)	mol/m³	
cR2NH2_p_in	(cAmine_tot/2)*alpha_k2	mol/m³	
cR2NH_dp_in	(cAmine_tot/2)*(1 - alpha_k2)	mol/m³	
cRNH3_p_in	(cAmine_tot/4)*alpha_k1	mol/m³	
cRNH2_dp_in	(cAmine_tot/4)*(1 - alpha_k1)	mol/m³	

Study 3 - CP - Gold HER Kinetics (DISABLE when not running Study 3!)

SELECTION

Geometric entity level	Boundary
Selection	Geometry geom1: Dimension 0: Boundary 1

Name	Expression	Unit	Description
r_WR	k2*cH2O_b	mol/(m²·s)	
r_PR	k1*max(cH, eps)	mol/(m²·s)	
k2	k_app2*exp((-alpha_WR)*f*(V_electrode))	m/s	
k1	k_app1*exp((-alpha_PR)*f*(V_electrode))	m/s	
j_HER	-r_PR - r_WR	mol/(m²·s)	
jC	Cdl*d(V_electrode, t)	A/m²	
j_H_proton	-r_PR	mol/(m²·s)	
j_OH	r_WR	mol/(m²·s)	

2.1.2 Functions

Study 1 - CV Waveform

Function name	Potential
Function type	Piecewise

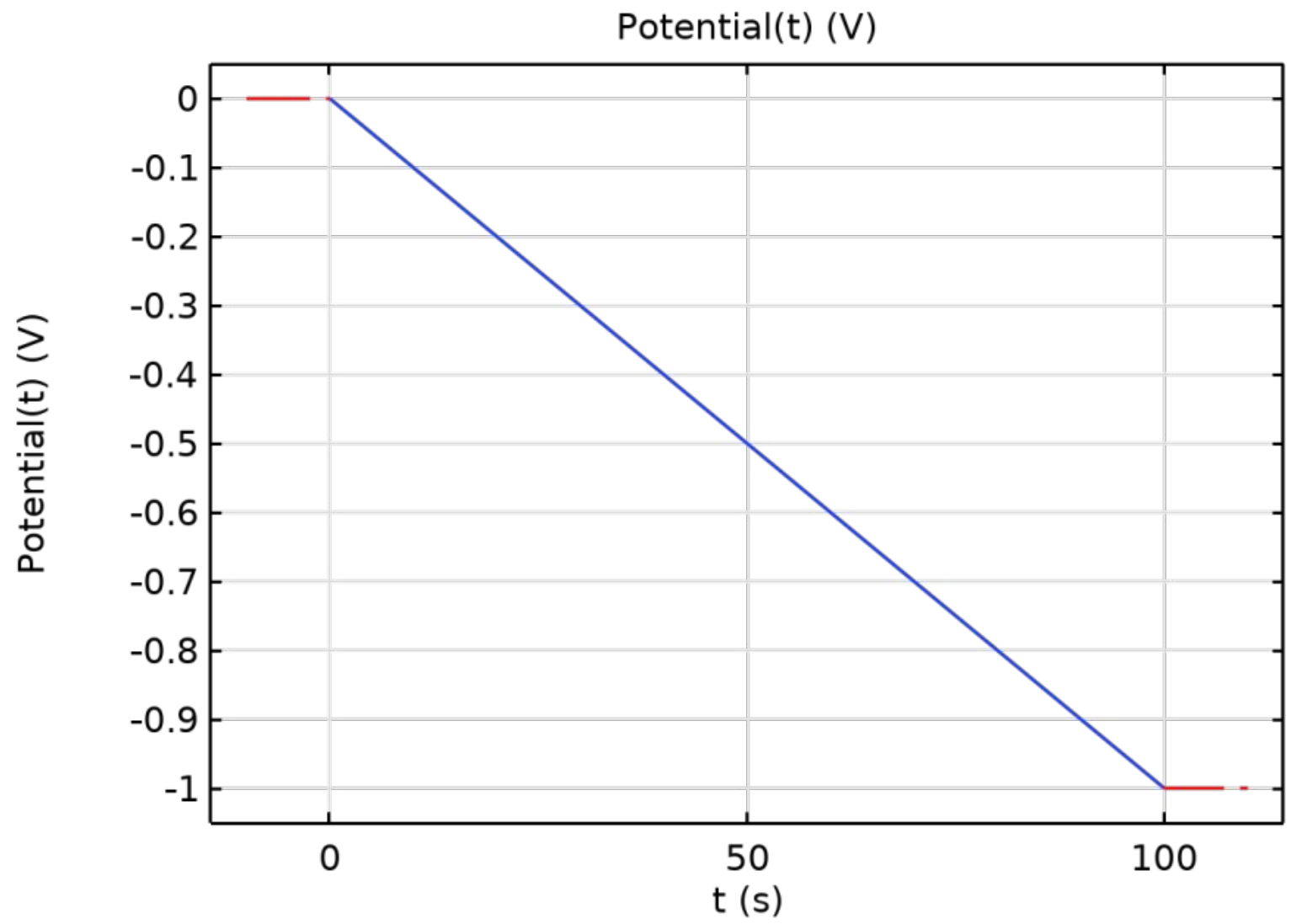

Study 1 - CV Waveform

DEFINITION

Description	Value
Argument	t
Extrapolation	Constant
Smoothing	No smoothing

DEFINITION

Start	End	Function
0	Scan_Duration	(E_Start-v*t)

UNITS

Description	Value
Arguments	s
Function	V

Study 2 - CA - Potential Step

Function name	Potential_Step
Function type	Piecewise

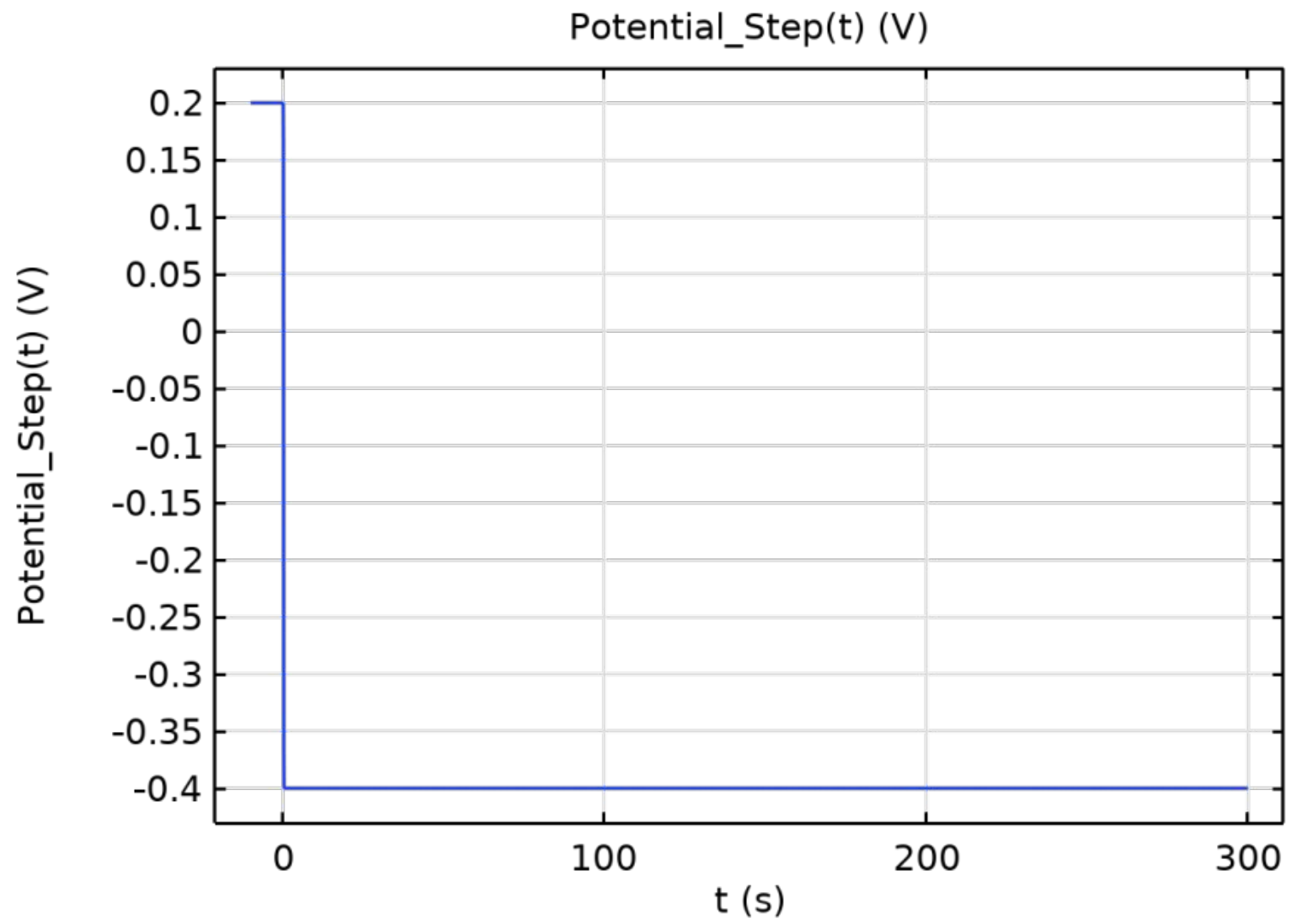

Study 2 - CA - Potential Step

DEFINITION

Description	Value
Argument	t
Extrapolation	None
Smoothing	No smoothing

DEFINITION

Start	End	Function
-10	300[s]	E_Step(t)

UNITS

Description	Value
Arguments	s
Function	V

Study 2

Function name	E_Step
Function type	Step

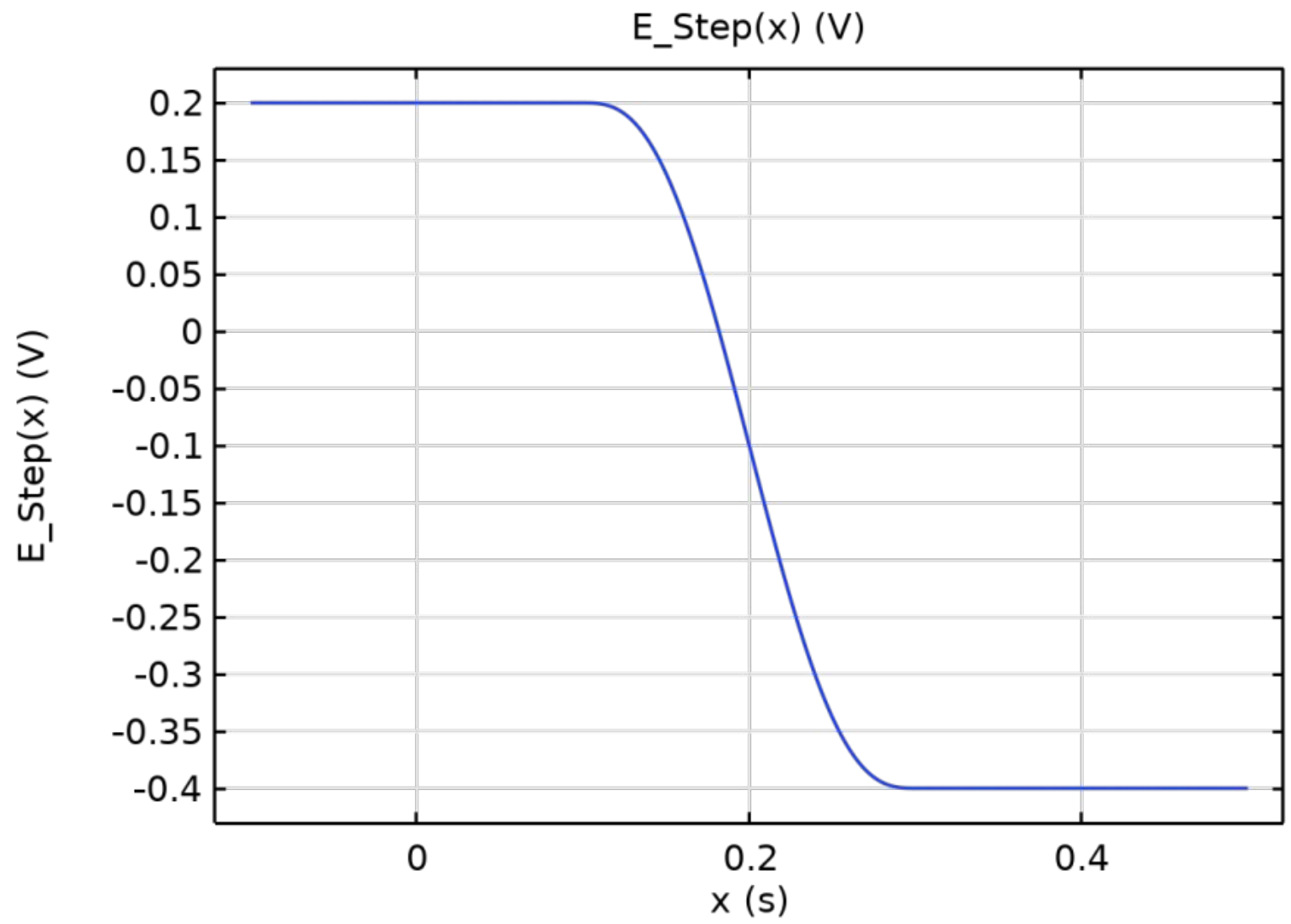

Study 2

PARAMETERS

Description	Value
Location	0.2[s]
From	0.2
To	E_App

SMOOTHING

Description	Value
Size of transition zone	0.2

Study 3 - CP - Current Density

Function name	j_App
Function type	Piecewise

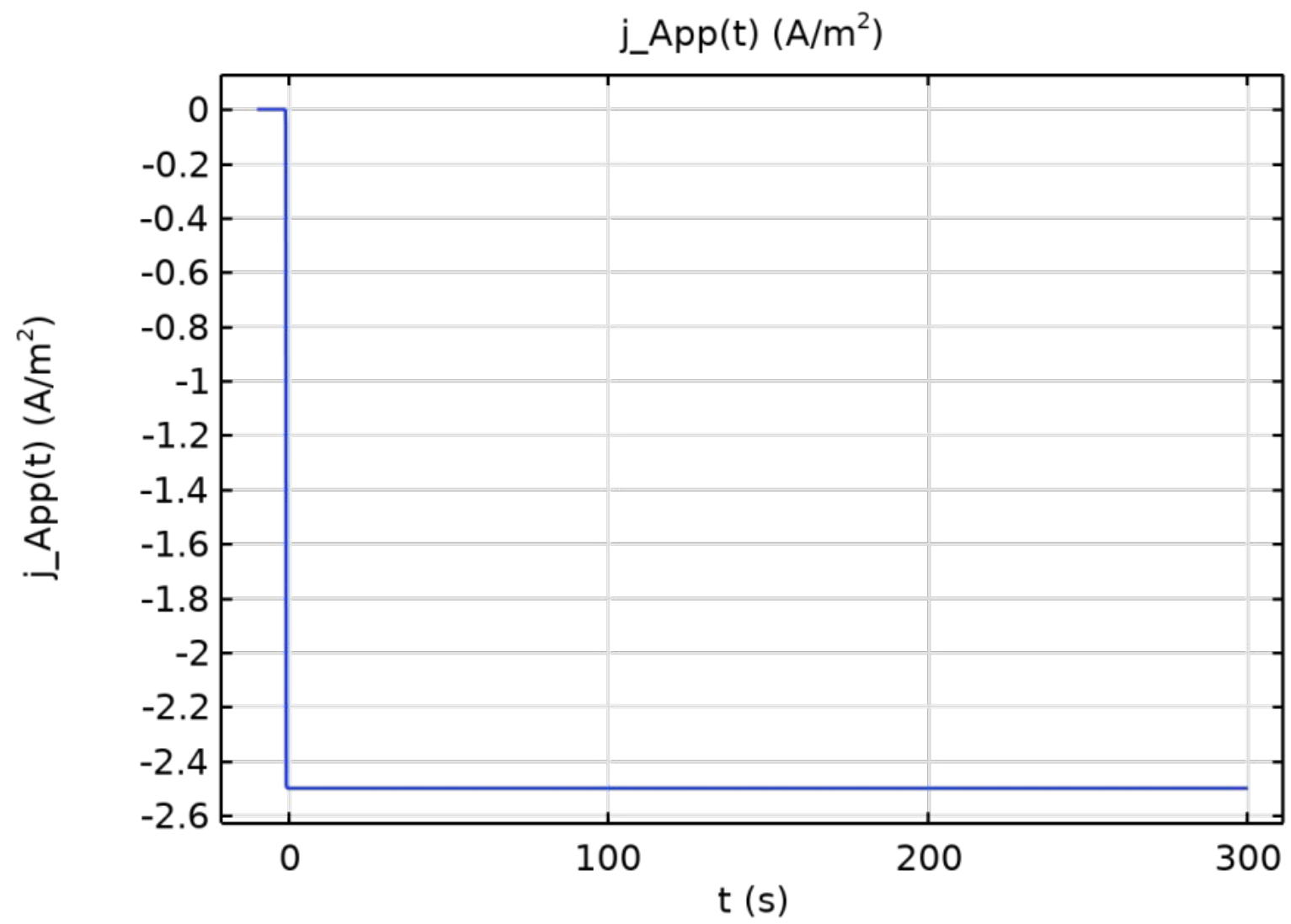

Study 3 - CP - Current Density

DEFINITION

Description	Value
Argument	t
Extrapolation	None
Smoothing	No smoothing

DEFINITION

Start	End	Function
-10	300[s]	Current_Density(t)

UNITS

Description	Value
Arguments	s
Function	A/m^2

Study 3 - Step 2

Function name	Current_Density
Function type	Step

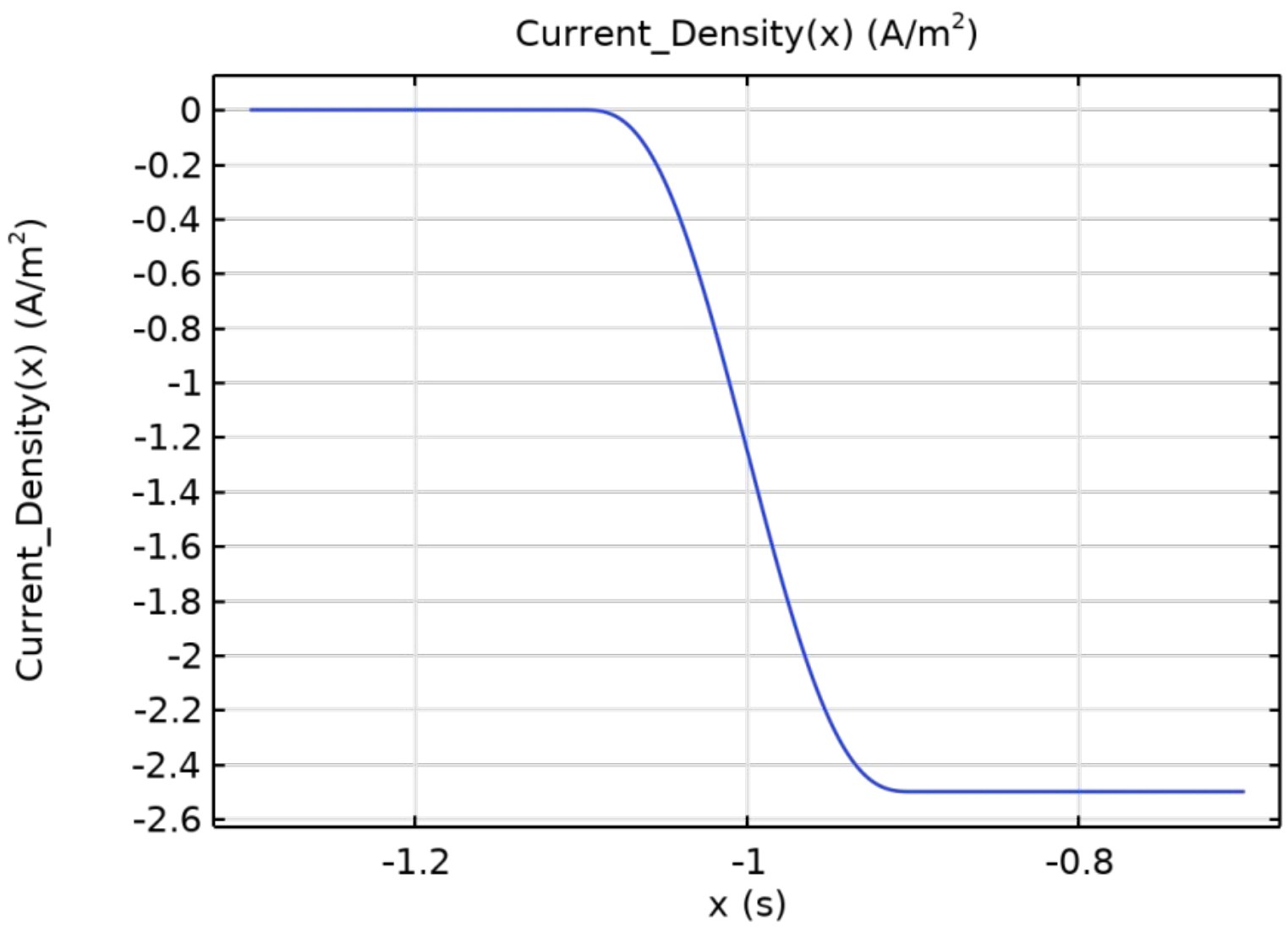

Study 3 - Step 2

PARAMETERS

Description	Value
Location	-1[s]
From	0
To	j_pulse

SMOOTHING

Description	Value
Size of transition zone	0.2

2.1.3 Probes

Total Current Density

Probe type	Point probe

SELECTION

Geometric entity level	Boundary
Selection	Geometry geom1: Dimension 0: Boundary 1

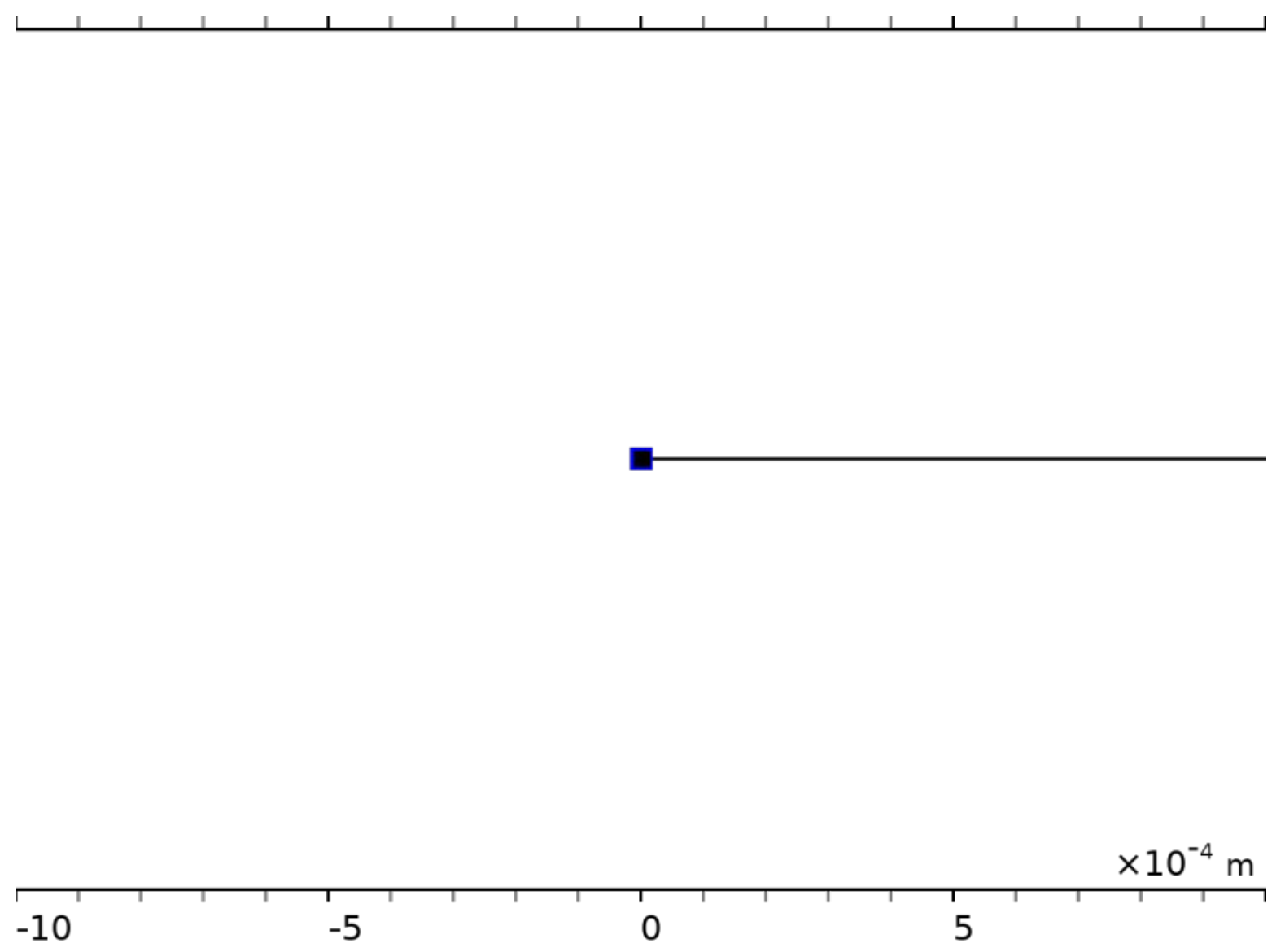

Selection

EXPRESSION

Description	Value
Expression	j_HER*F_const
Table and plot unit	A/m^2
Description	j_HER*F_const

TABLE AND WINDOW SETTINGS

Description	Value
Output table	Probe Table 1
Plot window	Probe Plot 3

Proton Reduction Current Density

Probe type	Point probe

SELECTION

Geometric entity level	Boundary
Selection	Geometry geom1: Dimension 0: Boundary 1

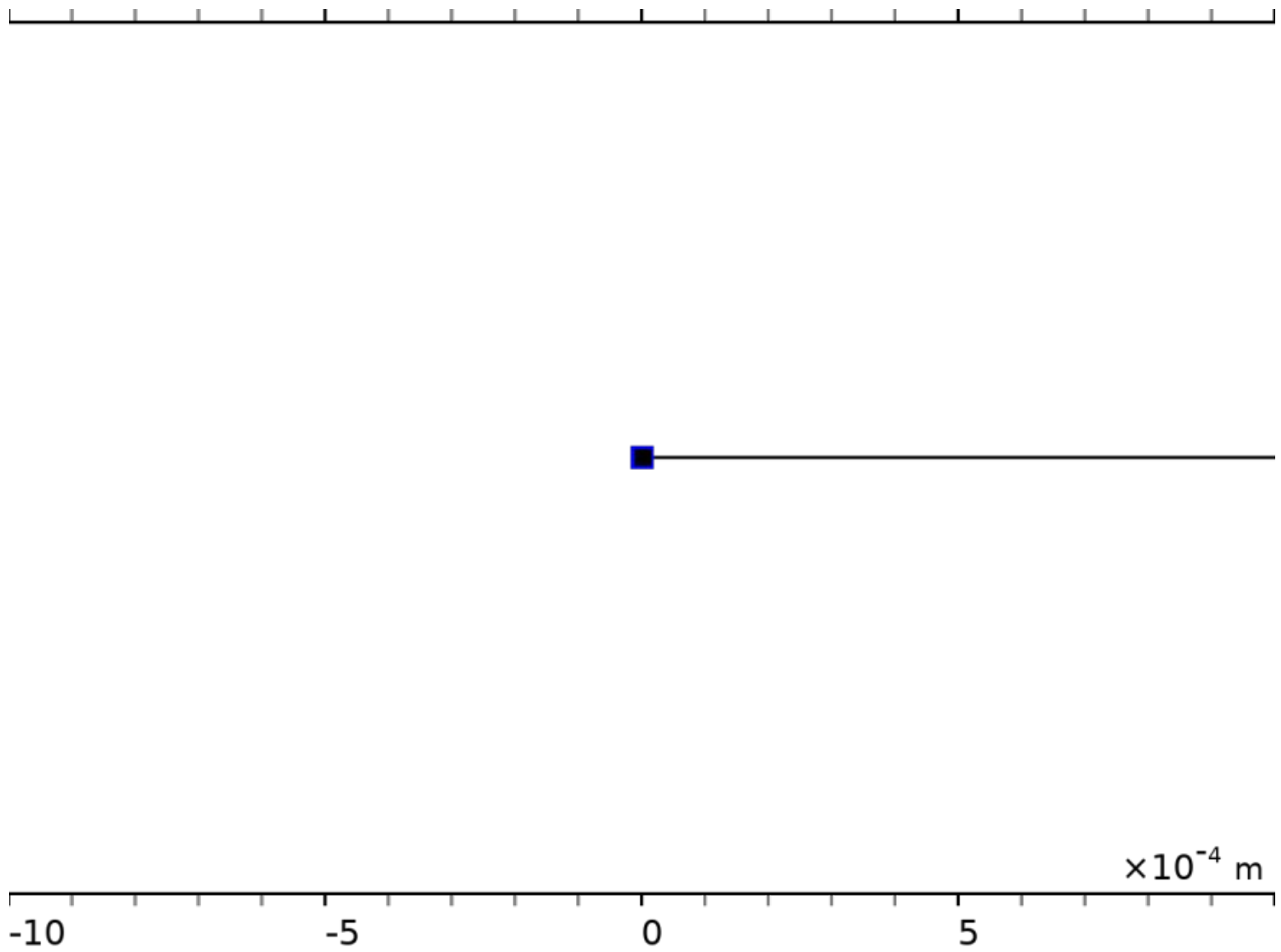

Selection

EXPRESSION

Description	Value
Expression	(j_H_proton)*F_const
Table and plot unit	A/m^2
Description	(j_H_proton)*F_const

TABLE AND WINDOW SETTINGS

Description	Value
Output table	Probe Table 1
Plot window	Probe Plot 3

Electrode Potential (Current Pulse Study 3)

Probe type	Point probe

SELECTION

Geometric entity level	Boundary
Selection	Geometry geom1: Dimension 0: Boundary 1

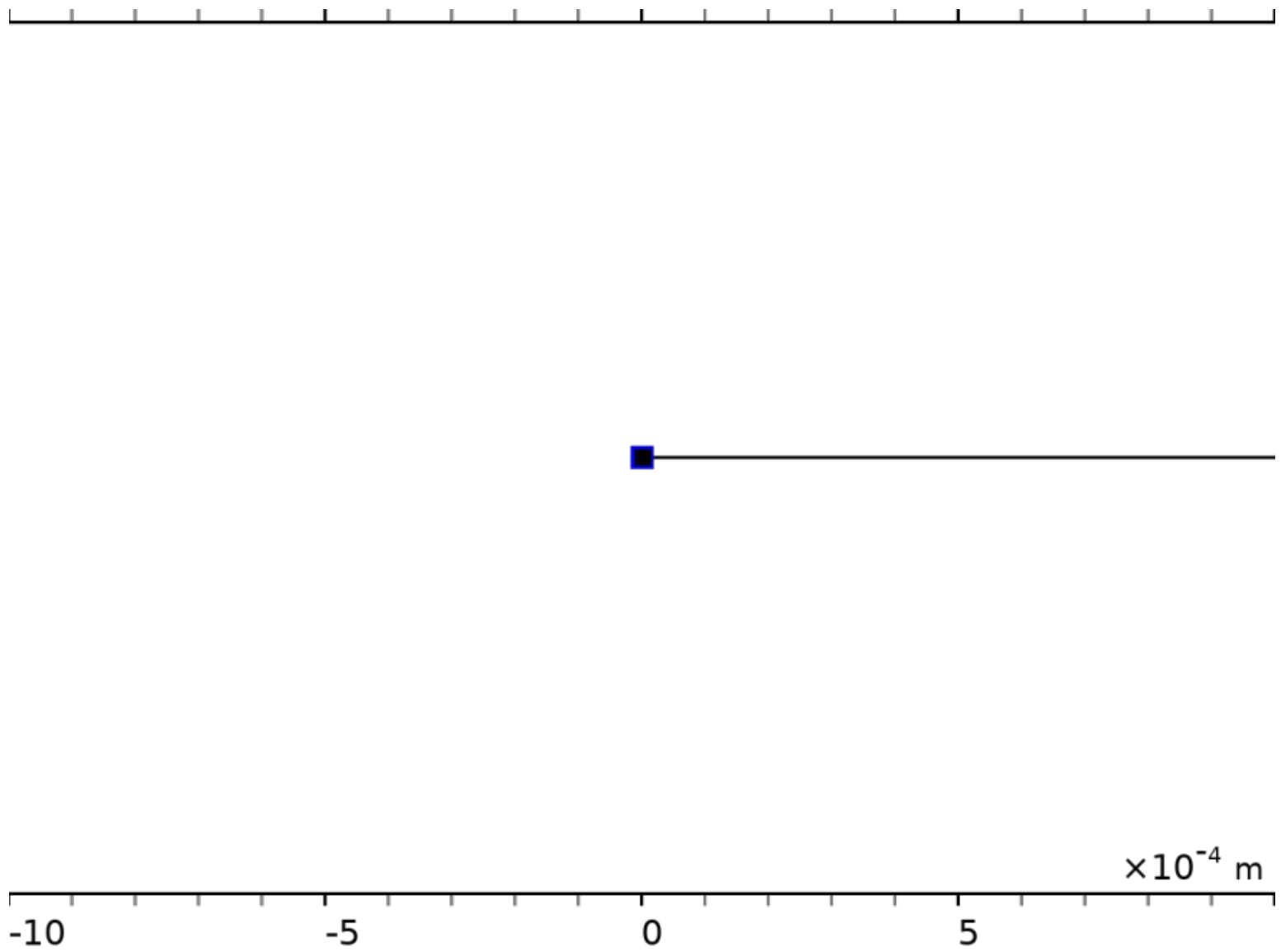

Selection

EXPRESSION

Description	Value
Expression	V_electrode
Table and plot unit	V
Description	State variable V_electrode

TABLE AND WINDOW SETTINGS

Description	Value
Output table	Probe Table 1
Plot window	Probe Plot 4

2.1.4 Nonlocal Couplings

Average Film pH

Coupling type	Average
Operator name	aveop1

SELECTION

Geometric entity level	Domain
Selection	Geometry geom1: Dimension 1: Domain 1

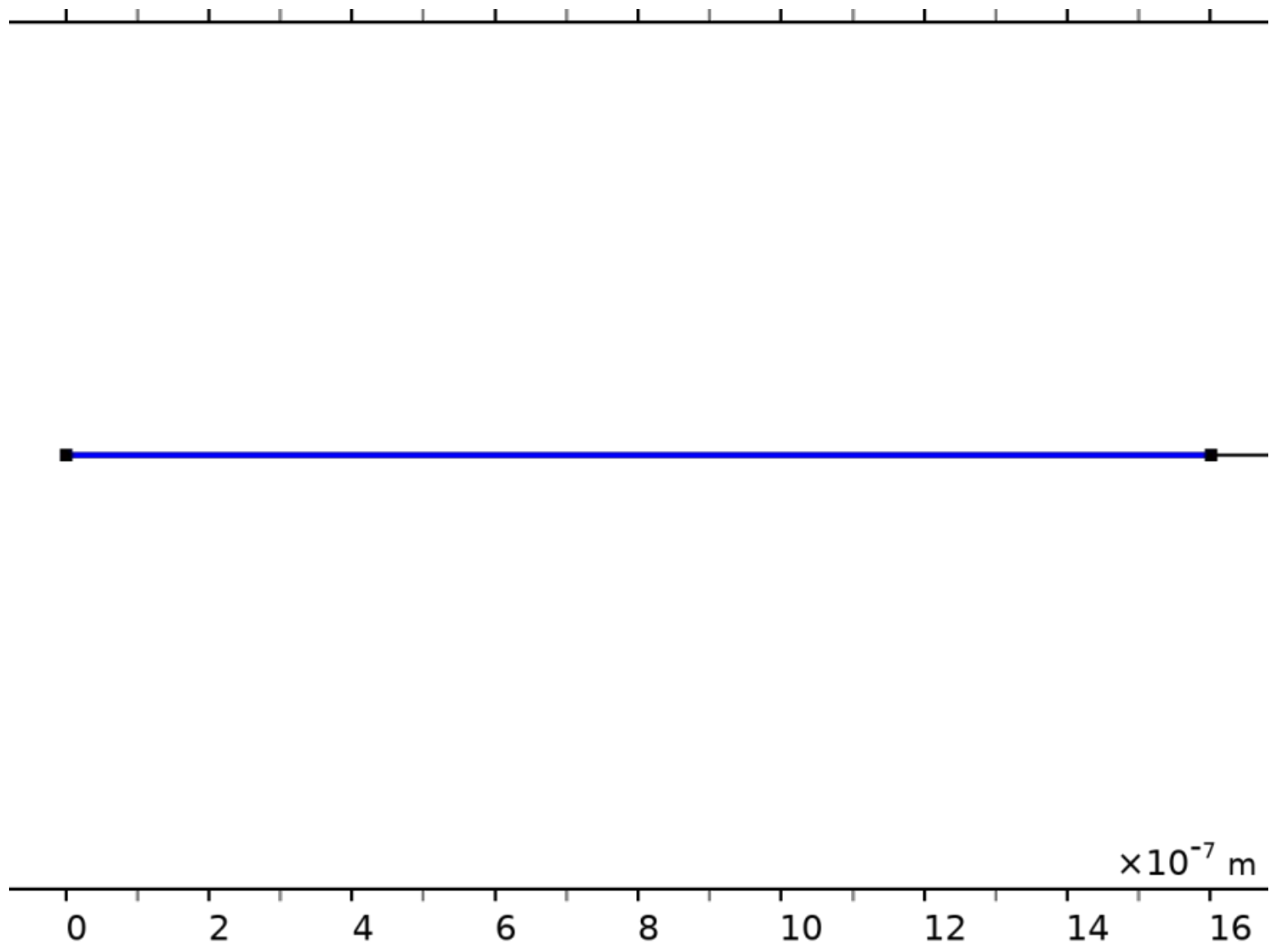

Selection

Integration 1

Coupling type	Integration
Operator name	intop1

SELECTION

Geometric entity level	Boundary
Selection	Geometry geom1: Dimension 0: Boundary 1

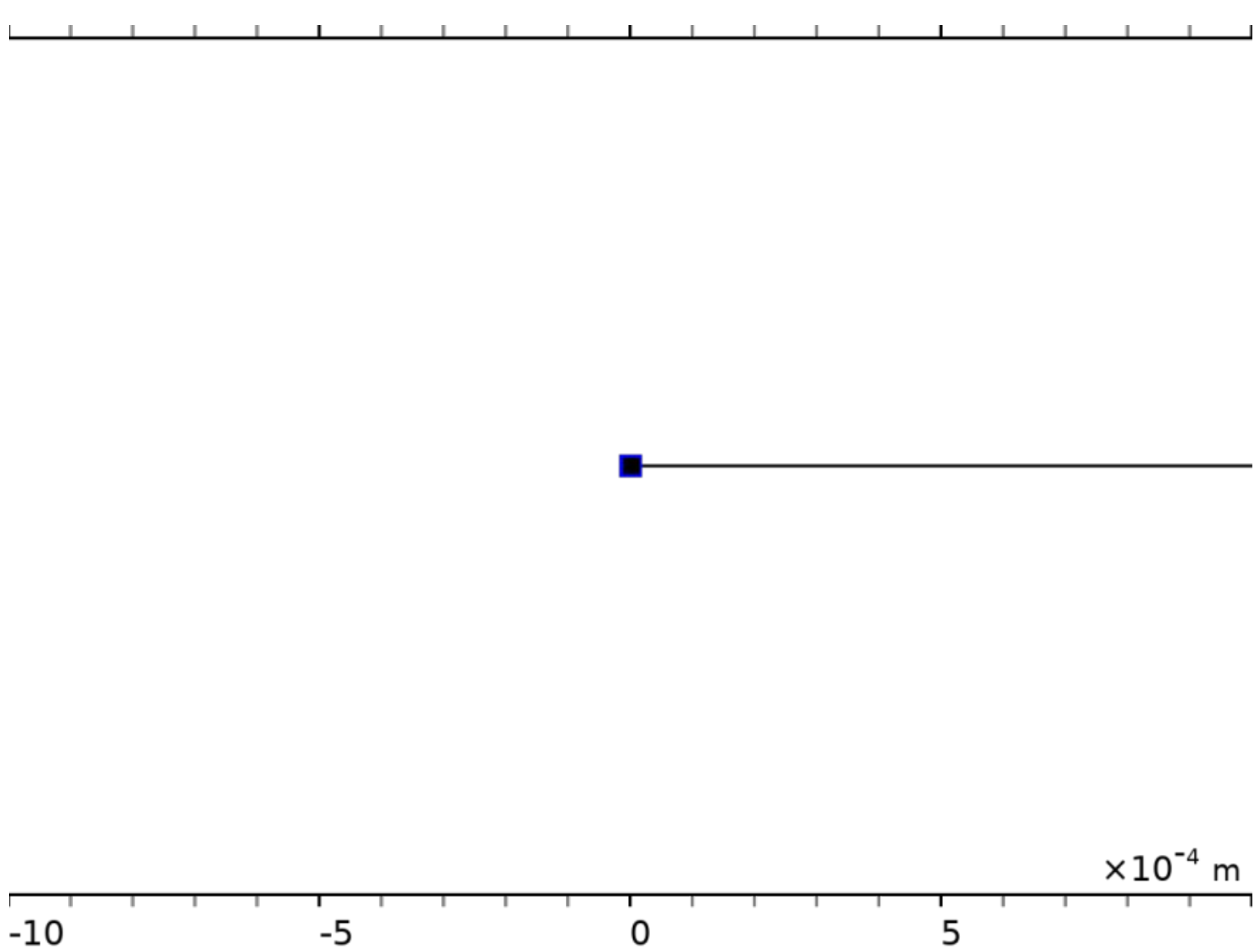

Selection

2.2 GEOMETRY 1

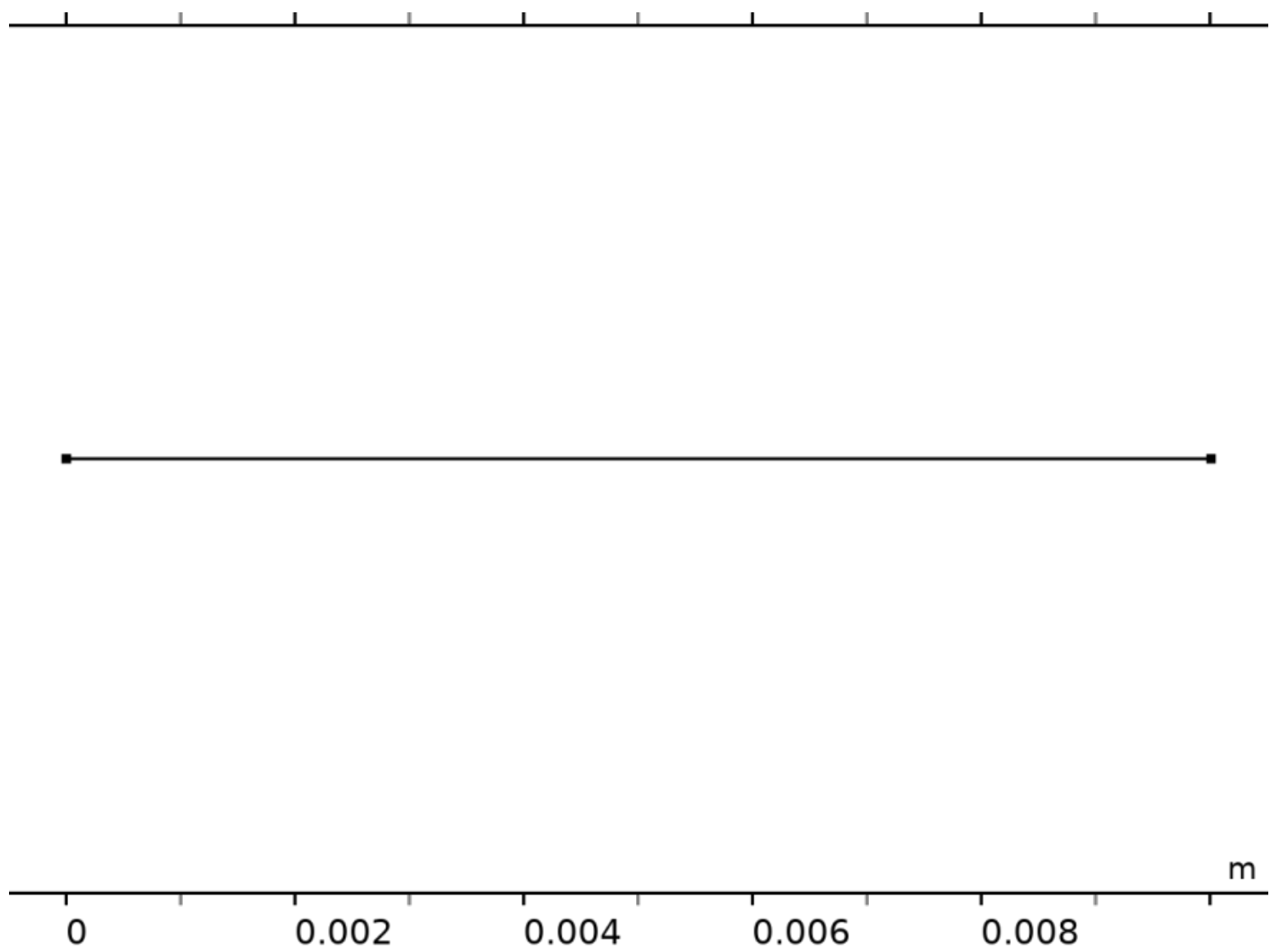

Geometry 1

UNITS

Length unit	m
Angular unit	deg

GEOMETRY STATISTICS

Description	Value
Space dimension	1
Number of domains	2
Number of boundaries	3

2.2.1 Interval 1 (i1)

INTERVAL

Coordinates (m)
0
L

2.2.2 Point 4 (pt4)

POSITION

Description	Value
Point coordinate	1.6E-6

2.2.3 Form Union (fin)

INFORMATION

Description	Value
Build message	Formed union of 1 solid object and 1 point object. Union has 2 domains and 3 boundaries.

2.3 HER

USED PRODUCTS

COMSOL Multiphysics
Chemical Reaction Engineering Module

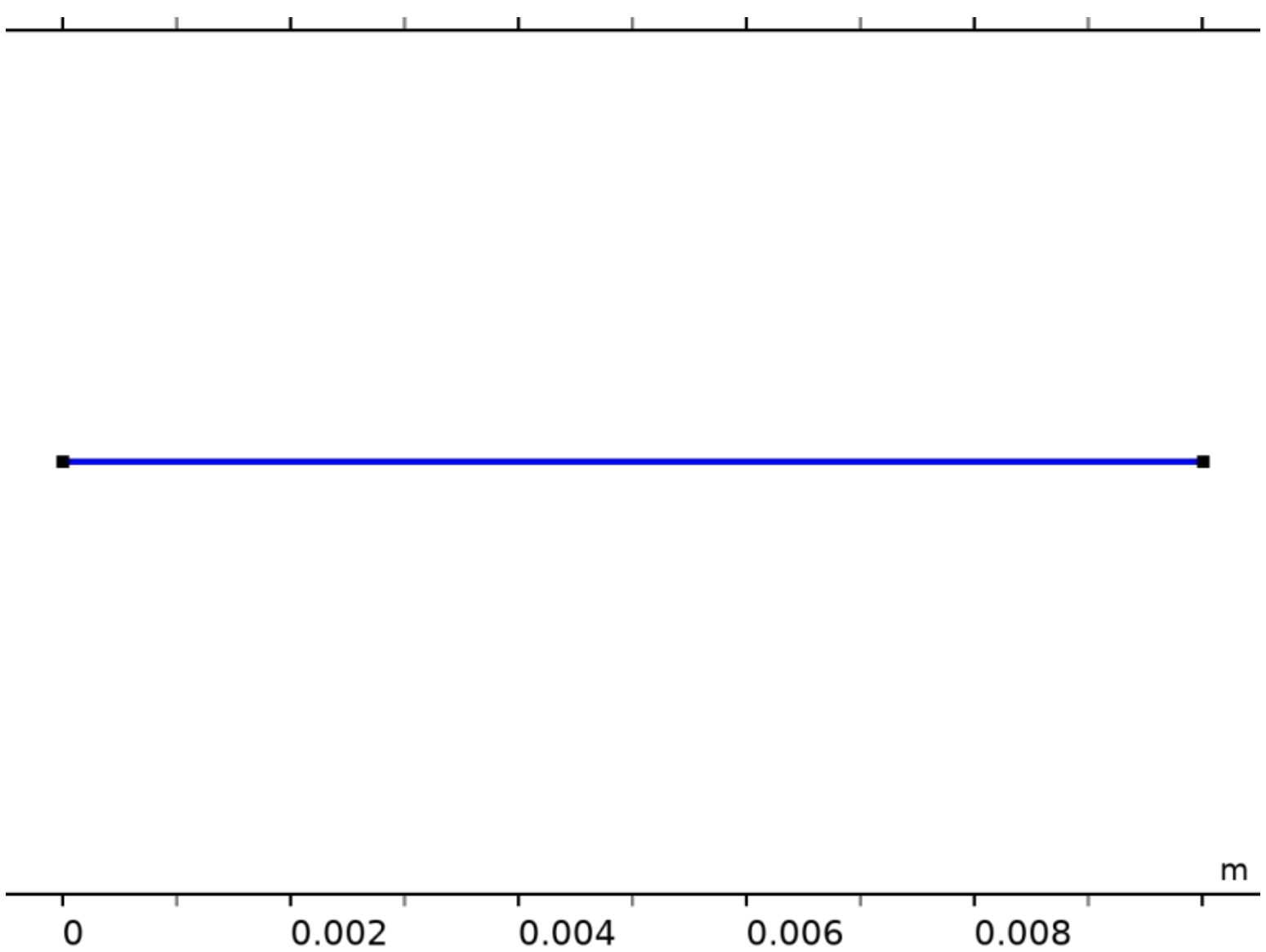

HER

SELECTION

Geometric entity level	Domain
Selection	Geometry geom1: Dimension 1: Domains 1–2

EQUATIONS

$$\frac{\partial c_i}{\partial t} + \nabla \cdot \mathbf{J}_i = R_i$$

$$\mathbf{J}_i = -D_i \nabla c_i$$

2.3.1 Interface Settings

Discretization

SETTINGS

Description	Value
Concentration	Linear

SETTINGS

Description	Value
Equation form	Study controlled

Cross-Sectional Area

SETTINGS

Description	Value	Unit
Cross-sectional area	1	m^2

Species Activity

SETTINGS

Description	Value
Species activity	Ideal

Transport Mechanisms

SETTINGS

Description	Value
Convection	Off
Migration in electric field	Off
Mass transfer in porous media	Off

2.3.2 Transport Properties - Solution

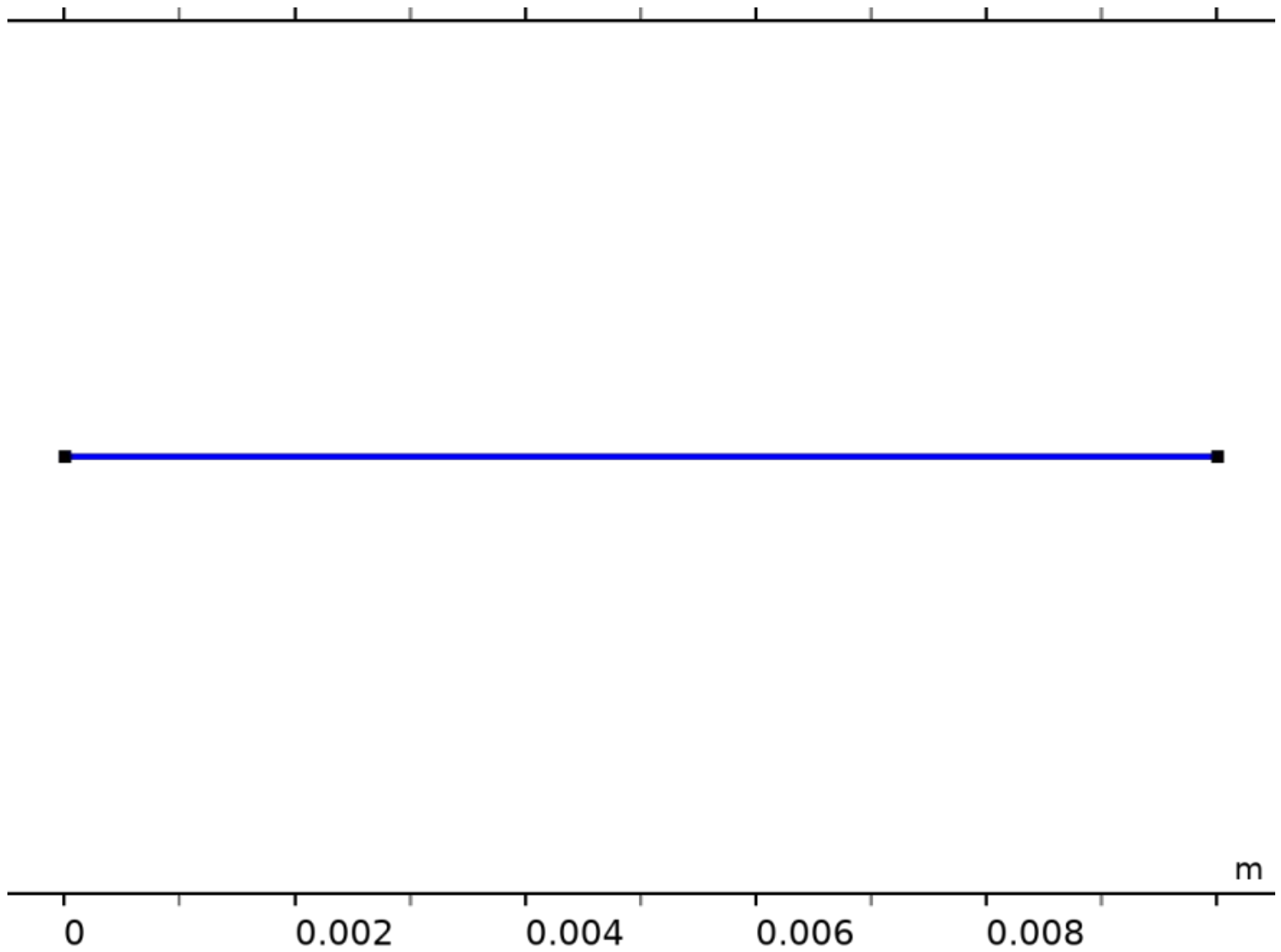

Transport Properties - Solution

SELECTION

Geometric entity level	Domain
Selection	Geometry geom1: Dimension 1: All domains

EQUATIONS

$$\frac{\partial c_i}{\partial t} + \nabla \cdot \mathbf{J}_i = R_i$$

$$\mathbf{J}_i = -D_i \nabla c_i$$

Diffusion

SETTINGS

Description	Value	Unit
Source	Material	
Material	None	
Diffusion coefficient	User defined	
Diffusion coefficient	D_H_proton	m^2/s
Diffusion coefficient	User defined	
Diffusion coefficient	D_OH	m^2/s

Coordinate System Selection

SETTINGS

Description	Value
Coordinate system	Global coordinate system

Model Input

SETTINGS

Description	Value	Unit
Temperature	User defined	
Temperature	T	K

2.3.3 No Flux 1

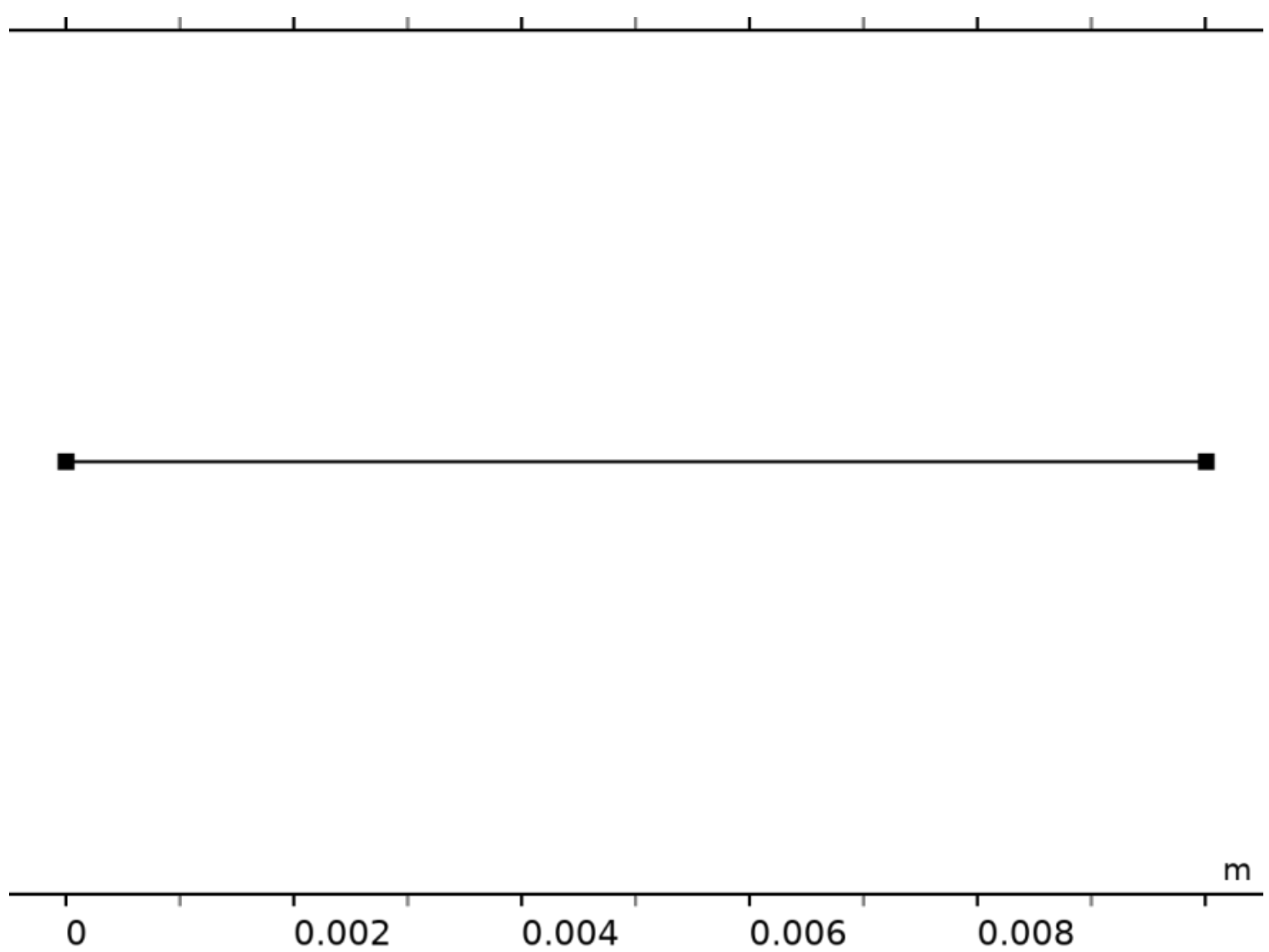

No Flux 1

SELECTION

Geometric entity level	Boundary
Selection	Geometry geom1: Dimension 0: All boundaries

EQUATIONS

$$-\mathbf{n}\cdot\mathbf{J}_i=0$$

2.3.4 Initial Values 1

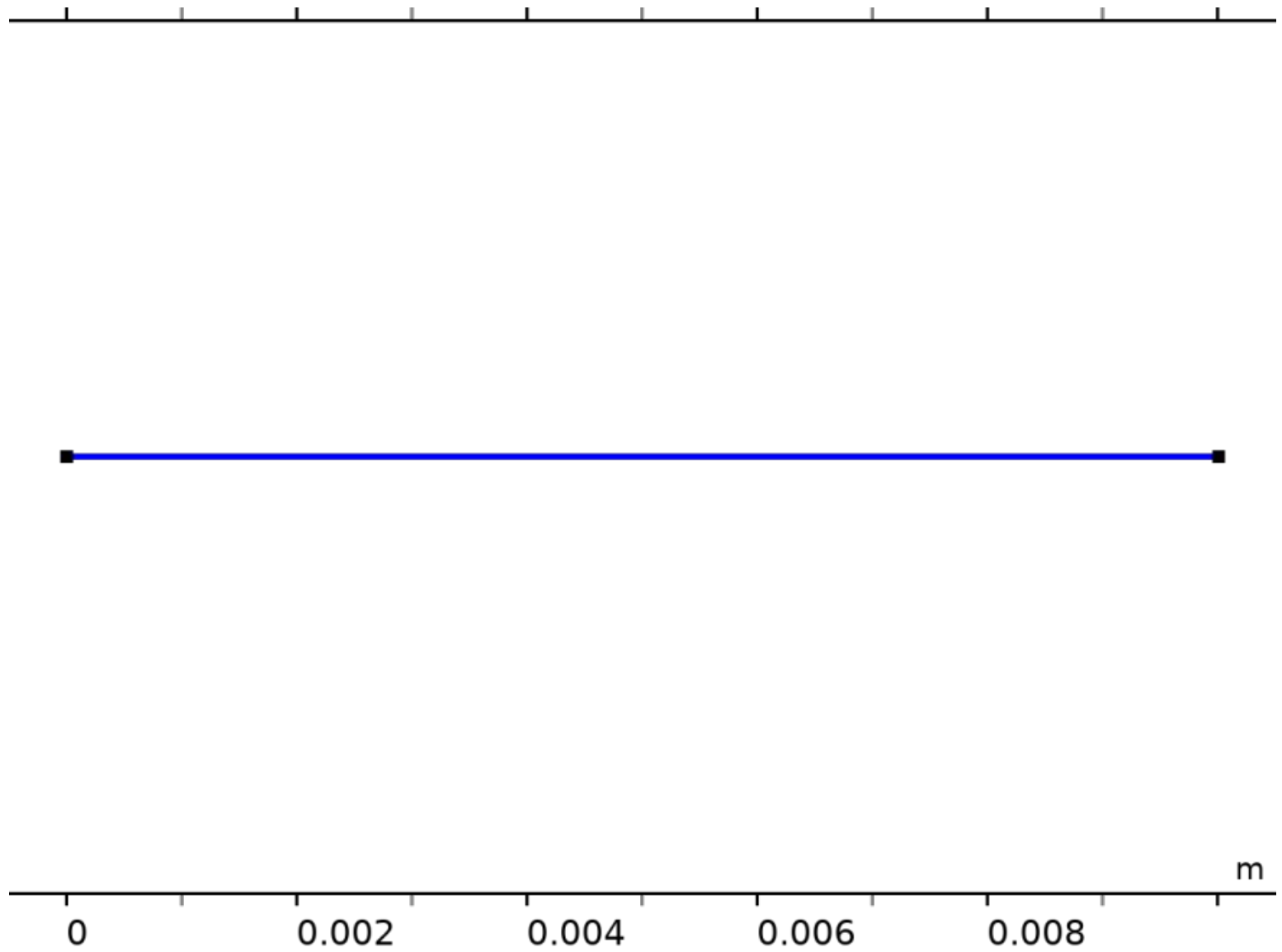

Initial Values 1

SELECTION

Geometric entity level	Domain
Selection	Geometry geom1: Dimension 1: All domains

Initial Values

SETTINGS

Description	Value	Unit
Concentration	cH_b	mol/m^3
Concentration	cOH_b	mol/m^3

2.3.5 Transport Properties - Film

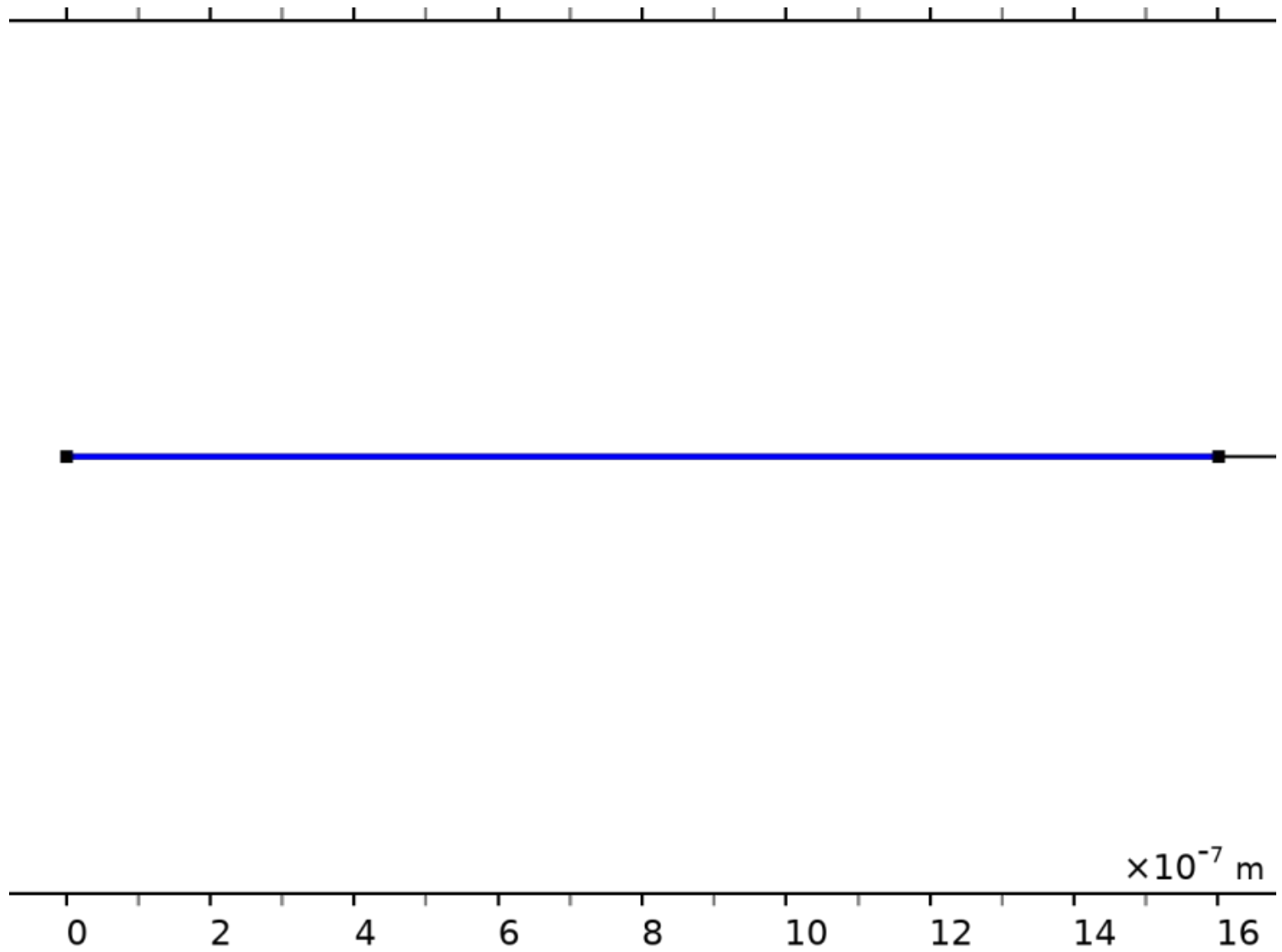

Transport Properties - Film

SELECTION

Geometric entity level	Domain
Selection	Geometry geom1: Dimension 1: Domain 1

EQUATIONS

$$\frac{\partial c_i}{\partial t} + \nabla \cdot \mathbf{J}_i = R_i$$

$$\mathbf{J}_i = -D_i \nabla c_i$$

Diffusion

SETTINGS

**Description**	**Value**	**Unit**
Source	Material	
Material	None	
Diffusion coefficient	User defined	
Diffusion coefficient	D_H_proton/Film_H_OH_Scale	m^2/s
Diffusion coefficient	User defined	
Diffusion coefficient	D_OH/Film_H_OH_Scale	m^2/s

Coordinate System Selection

SETTINGS

Description	Value
Coordinate system	Global coordinate system

Model Input

SETTINGS

Description	Value	Unit
Temperature	User defined	
Temperature	T	K

2.3.6 Concentration 1

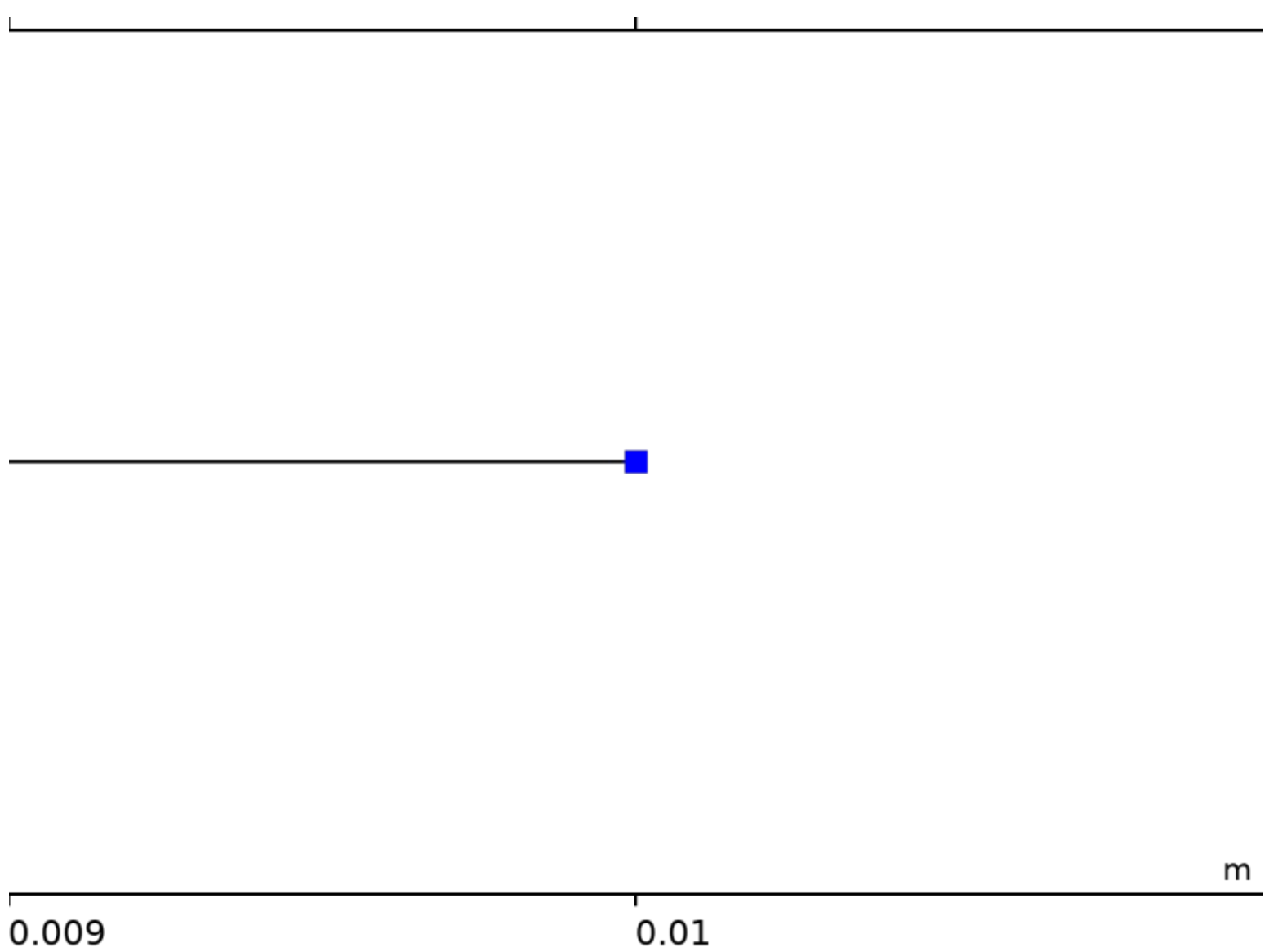

Concentration 1

SELECTION

Geometric entity level	Boundary
Selection	Geometry geom1: Dimension 0: Boundary 3

EQUATIONS

$$c_i = c_{0,j}$$

Concentration

SETTINGS

Description	Value	Unit
Species cH	On	
Species cOH	On	
Concentration	cH_b	mol/m^3

Description	Value	Unit
Concentration	cOH_b	mol/m^3

2.3.7 HER kinetics

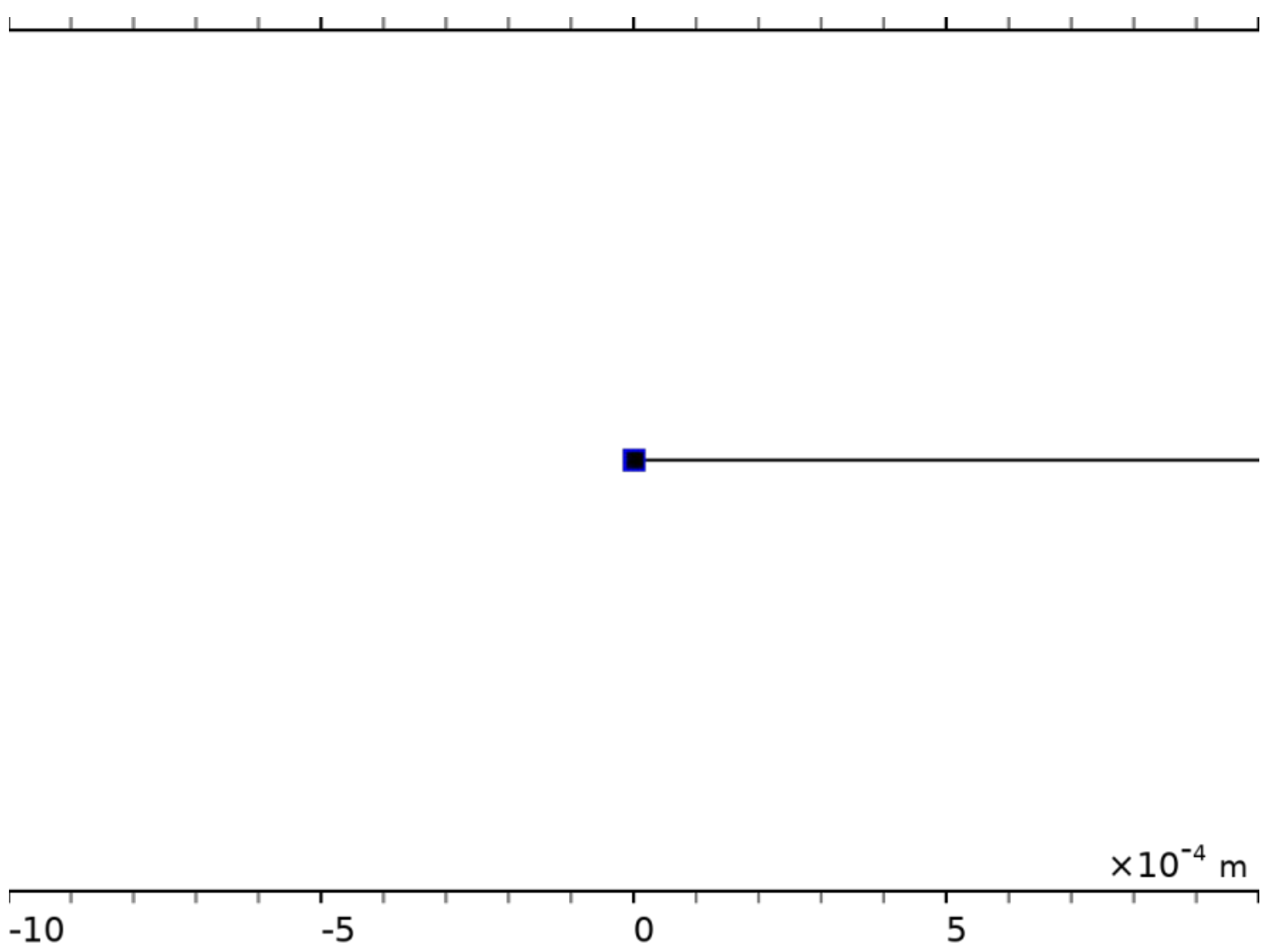

HER kinetics

SELECTION

Geometric entity level	Boundary
Selection	Geometry geom1: Dimension 0: Boundary 1

EQUATIONS

$$-\mathbf{n}\cdot\mathbf{J}_i = J_{0,i}$$

Inward Flux

SETTINGS

Description	Value	Unit
Flux type	General inward flux	
Species cH	On	
Species cOH	On	
	j_H_proton	mol/(m^2·s)
	j_OH	mol/(m^2·s)

2.3.8 Chemical Reactions - Solution

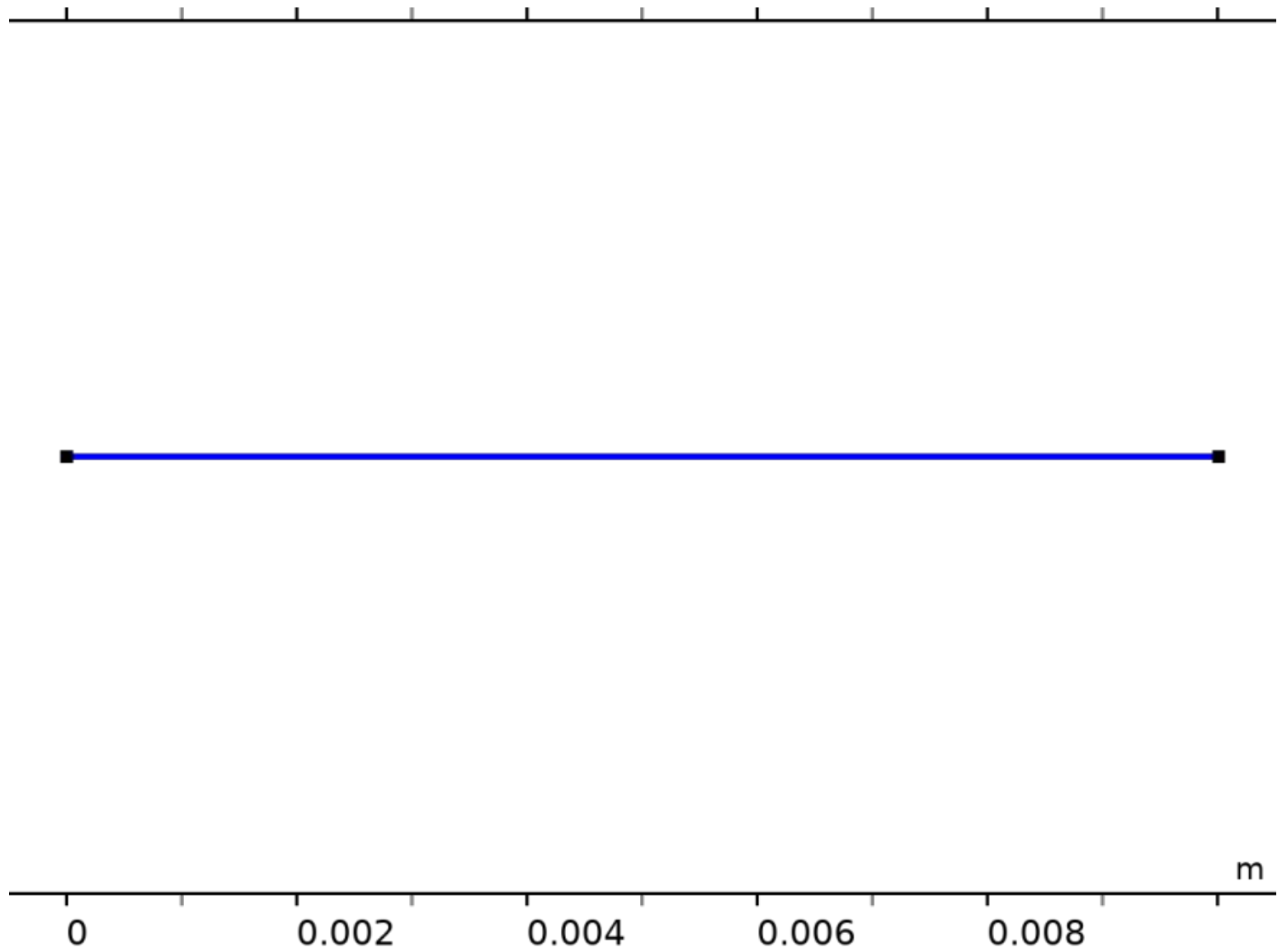

Chemical Reactions - Solution

SELECTION

Geometric entity level	Domain
Selection	Geometry geom1: Dimension 1: All domains

EQUATIONS

$$\frac{\partial c_i}{\partial t} + \nabla \cdot \mathbf{J}_i = R_i + S_i$$

Reaction Rates

SETTINGS

Description	Value	Unit
Chemistry	User defined	
Total rate expression	User defined	
Total rate expression	r_water + r_H3PO4 + r_H2PO4 + r_HPO4	mol/(m³·s)
Total rate expression	User defined	
Total rate expression	r_water	mol/(m³·s)

Heat Source

SETTINGS

Description	Value	Unit
Heat source	User defined	
Heat source	0	W/m³

2.3.9 Chemical Reactions - Film

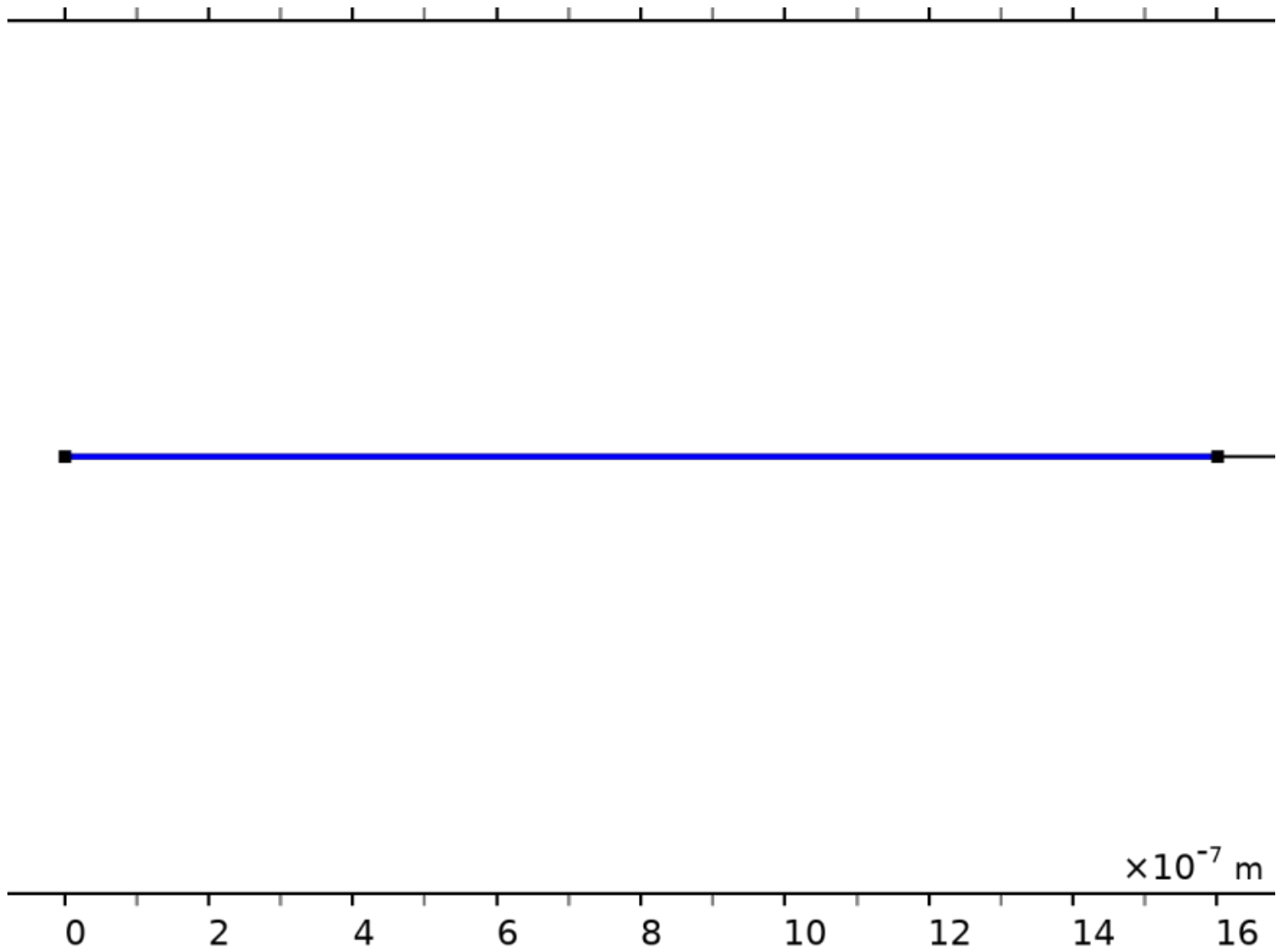

Chemical Reactions - Film

SELECTION

Geometric entity level	Domain
Selection	Geometry geom1: Dimension 1: Domain 1

EQUATIONS

$$\frac{\partial c_i}{\partial t} + \nabla \cdot \mathbf{J}_i = R_i + S_i$$

Reaction Rates

SETTINGS

Description	Value	Unit
Chemistry	User defined	
Total rate expression	User defined	
Total rate expression	r_side_chain_primary + r_side_chain_secondary + r_side_chain_tertiary	mol/(m³·s)
Total rate expression	User defined	
Total rate expression	0	mol/(m³·s)

Heat Source

SETTINGS

Description	Value	Unit
Heat source	User defined	
Heat source	0	W/m³

2.4 PHOSPHATE

USED PRODUCTS

COMSOL Multiphysics
Chemical Reaction Engineering Module

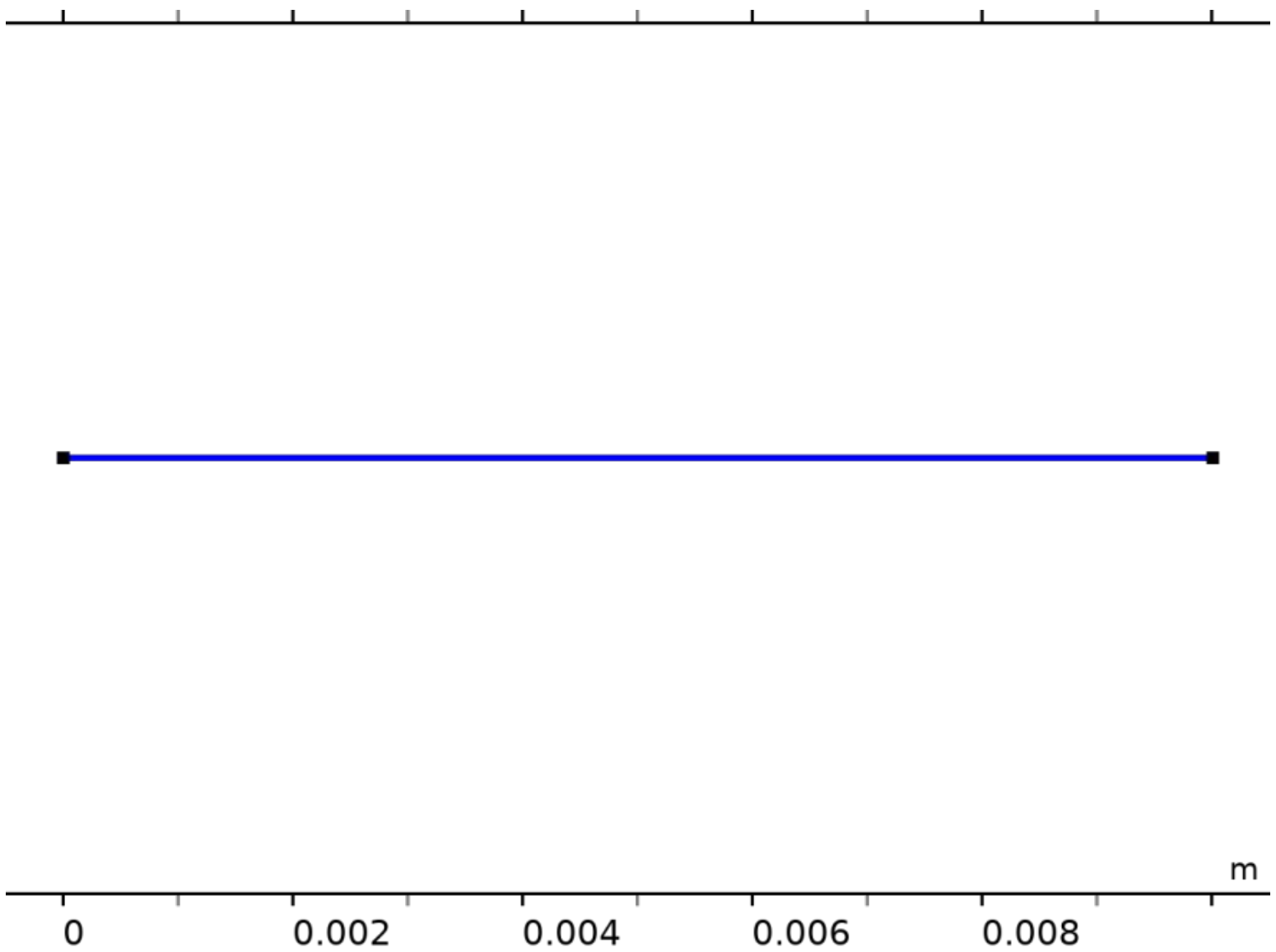

Phosphate

SELECTION

Geometric entity level	Domain
Selection	Geometry geom1: Dimension 1: All domains

EQUATIONS

$$\frac{\partial c_i}{\partial t} + \nabla \cdot \mathbf{J}_i = R_i$$
$$\mathbf{J}_i = -D_i \nabla c_i$$

2.4.1 Interface Settings

Discretization

SETTINGS

Description	Value
Concentration	Linear

SETTINGS

Description	Value
Equation form	Study controlled

Cross-Sectional Area

SETTINGS

Description	Value	Unit
Cross-sectional area	1	m^2

Species Activity

SETTINGS

Description	Value
Species activity	Ideal

Transport Mechanisms

SETTINGS

Description	Value
Convection	Off
Migration in electric field	Off
Mass transfer in porous media	Off

2.4.2 Transport Properties - Solution

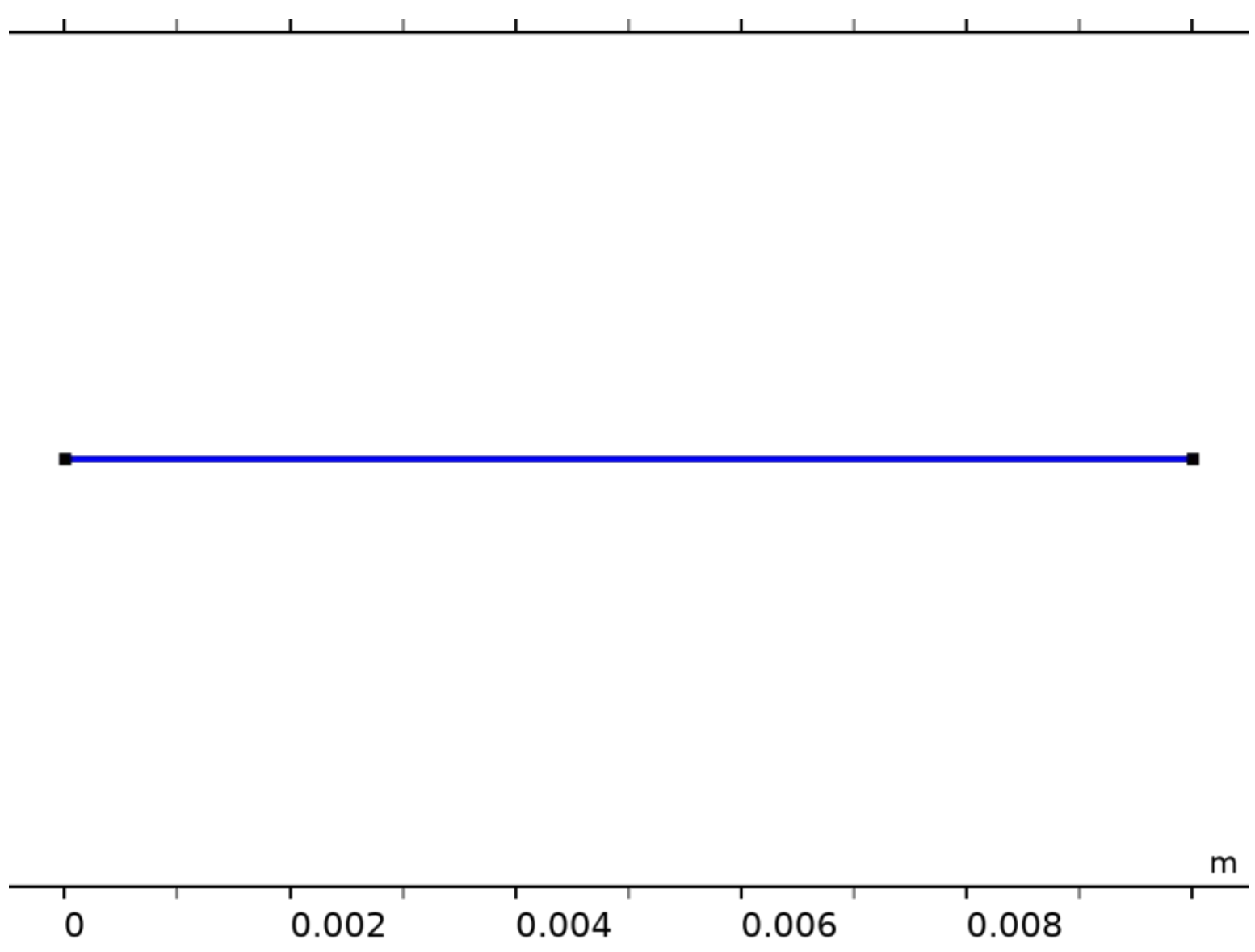

Transport Properties - Solution

SELECTION

Geometric entity level	Domain
Selection	Geometry geom1: Dimension 1: All domains

EQUATIONS

$$\nabla \cdot \mathbf{J}_i = R_i$$
$$\mathbf{J}_i = -D_i \nabla c_i$$

Diffusion

SETTINGS

Description	Value	Unit
Source	Material	
Material	None	
Diffusion coefficient	User defined	
Diffusion coefficient	D_H3PO4	m^2/s
Diffusion coefficient	User defined	
Diffusion coefficient	D_H2PO4	m^2/s
Diffusion coefficient	User defined	
Diffusion coefficient	D_HPO4	m^2/s
Diffusion coefficient	User defined	
Diffusion coefficient	D_PO4	m^2/s

Coordinate System Selection

SETTINGS

Description	Value
Coordinate system	Global coordinate system

Model Input

SETTINGS

Description	Value	Unit
Temperature	User defined	
Temperature	T	K

2.4.3 No Flux 1

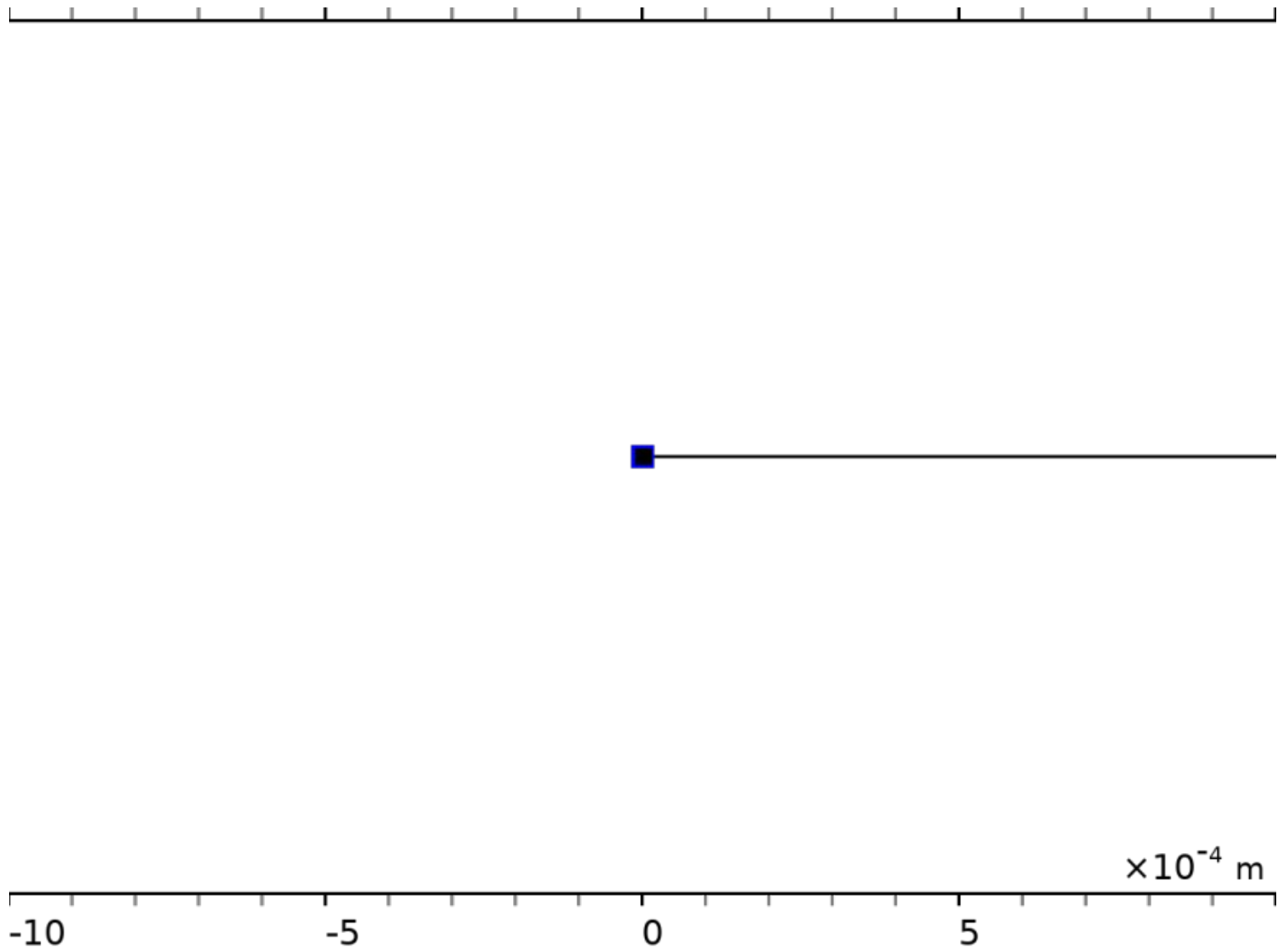

No Flux 1

SELECTION

Geometric entity level	Boundary
Selection	Geometry geom1: Dimension 0: All boundaries

EQUATIONS

$$-\mathbf{n}\cdot\mathbf{J}_i=0$$

2.4.4 Initial Values - Solution + Film

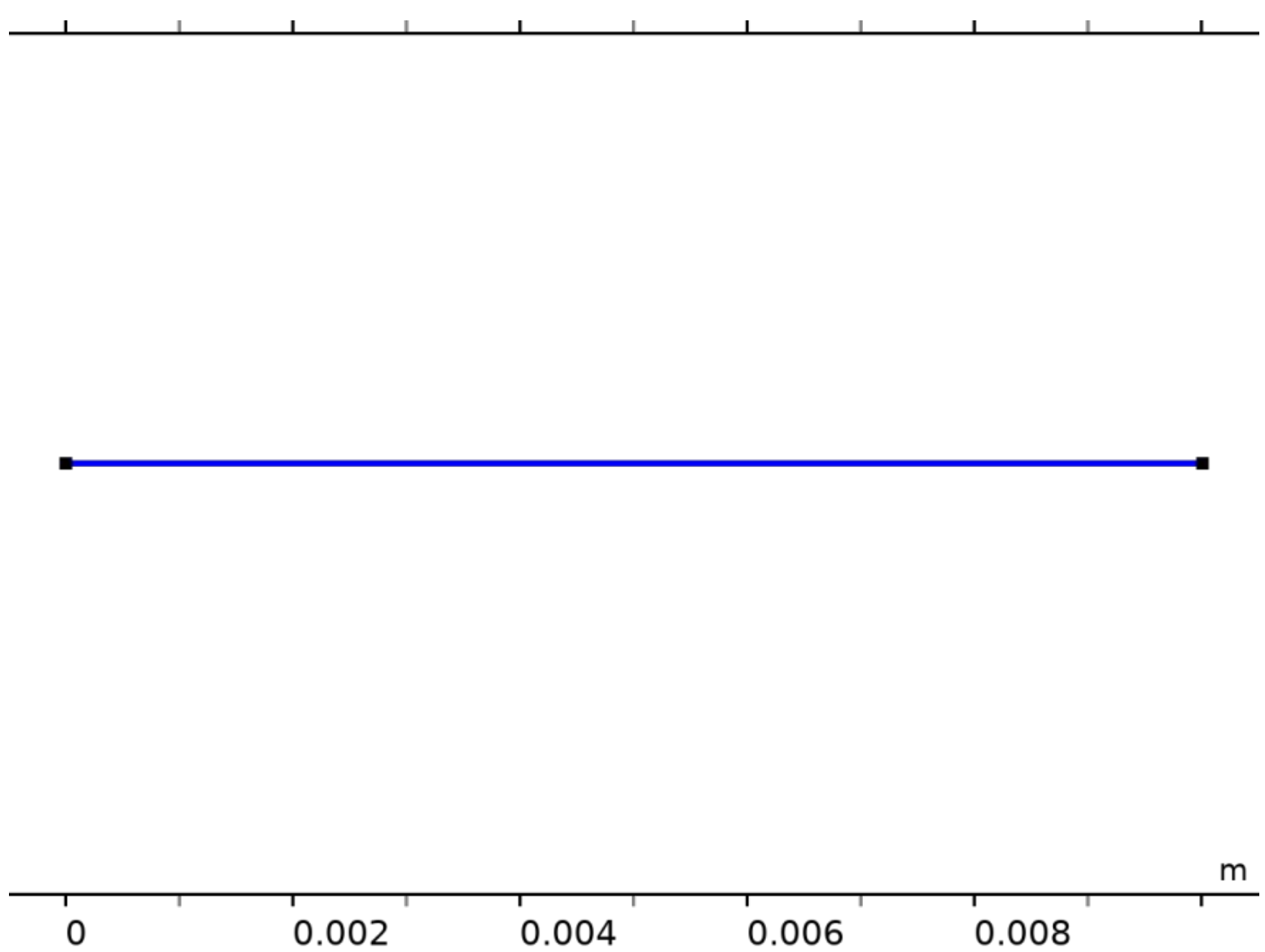

Initial Values - Solution + Film

SELECTION

Geometric entity level	Domain
Selection	Geometry geom1: Dimension 1: All domains

Initial Values

SETTINGS

Description	Value	Unit
Concentration	cH3PO4_b	mol/m³
Concentration	cH2PO4_b	mol/m³
Concentration	cHPO4_b	mol/m³
Concentration	cPO4_b	mol/m³

2.4.5 Transport Properties - Film

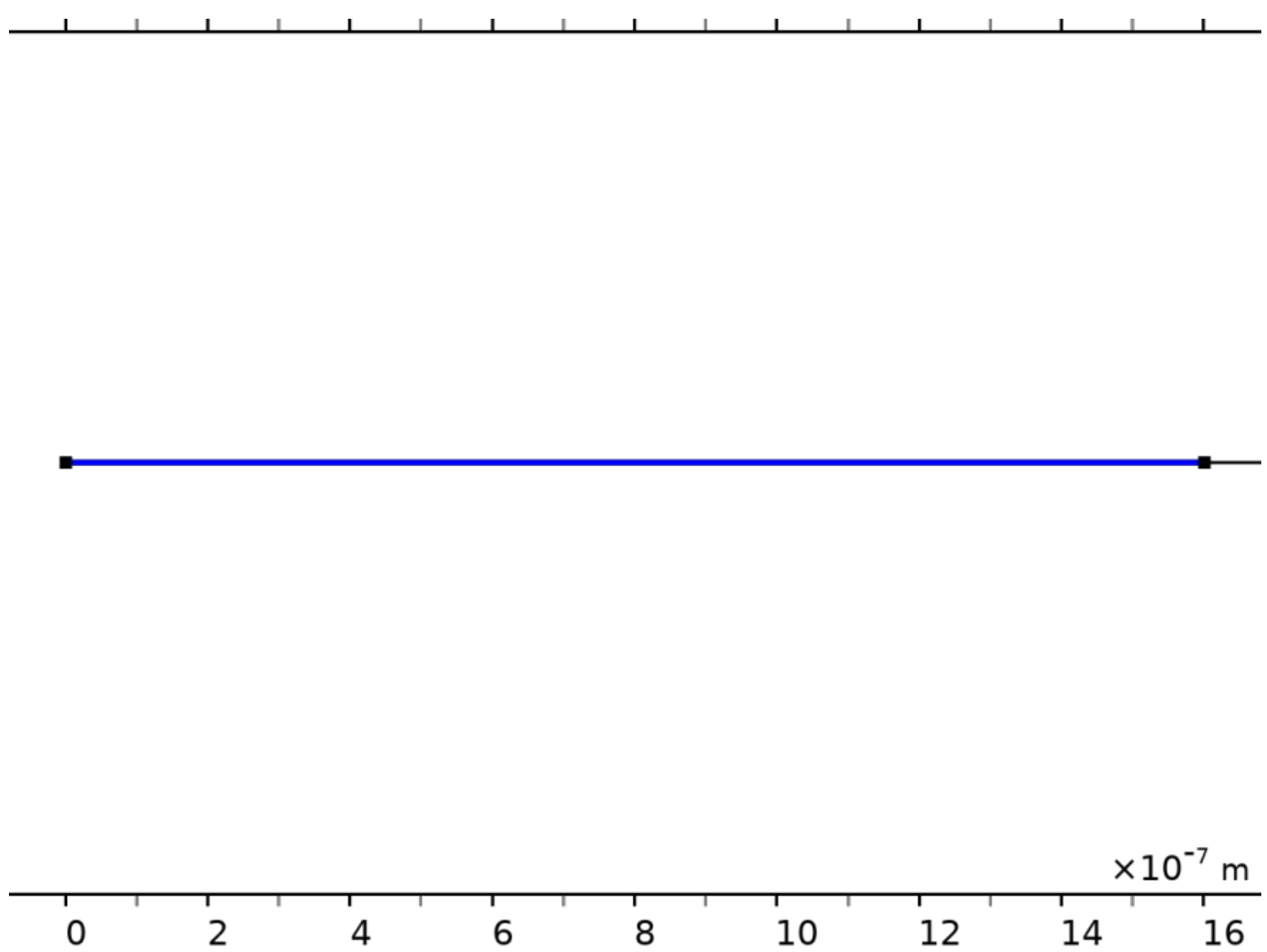

Transport Properties - Film

SELECTION

Geometric entity level	Domain
Selection	Geometry geom1: Dimension 1: Domain 1

EQUATIONS

$$\nabla \cdot \mathbf{J}_i = R_i$$
$$\mathbf{J}_i = -D_i \nabla c_i$$

Diffusion

SETTINGS

Description	Value	Unit
Source	Material	
Material	None	
Diffusion coefficient	User defined	
Diffusion coefficient	D_H3PO4/Film_Phosphate_Scale	m^2/s
Diffusion coefficient	User defined	
Diffusion coefficient	D_H2PO4/Film_Phosphate_Scale	m^2/s
Diffusion coefficient	User defined	
Diffusion coefficient	D_HPO4/Film_Phosphate_Scale	m^2/s
Diffusion coefficient	User defined	
Diffusion coefficient	D_PO4/Film_Phosphate_Scale	m^2/s

Coordinate System Selection

SETTINGS

Description	Value
Coordinate system	Global coordinate system

Model Input

SETTINGS

Description	Value	Unit
Temperature	User defined	
Temperature	T	K

2.4.6 Concentration - Bulk

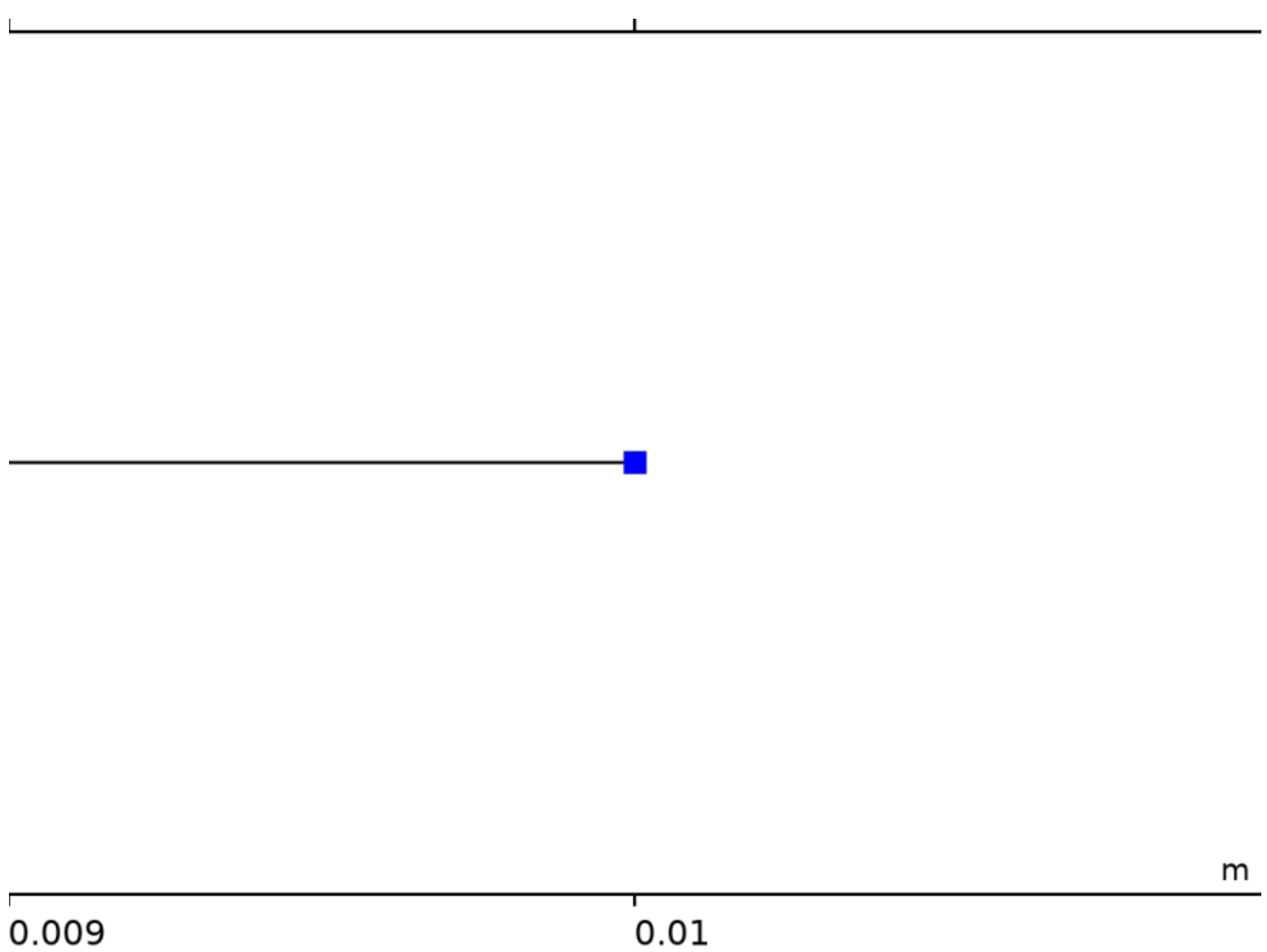

Concentration - Bulk

SELECTION

Geometric entity level	Boundary
Selection	Geometry geom1: Dimension 0: Boundary 3

EQUATIONS

$$c_i = c_{0,j}$$

Concentration

SETTINGS

Description	Value	Unit
Species cH3PO4	On	
Species cH2PO4	On	
Species cHPO4	On	
Species cPO4	On	
Concentration	cH3PO4_b	mol/m^3
Concentration	cH2PO4_b	mol/m^3
Concentration	cHPO4_b	mol/m^3
Concentration	cPO4_b	mol/m^3

2.4.7 Reactions - Film + Solution

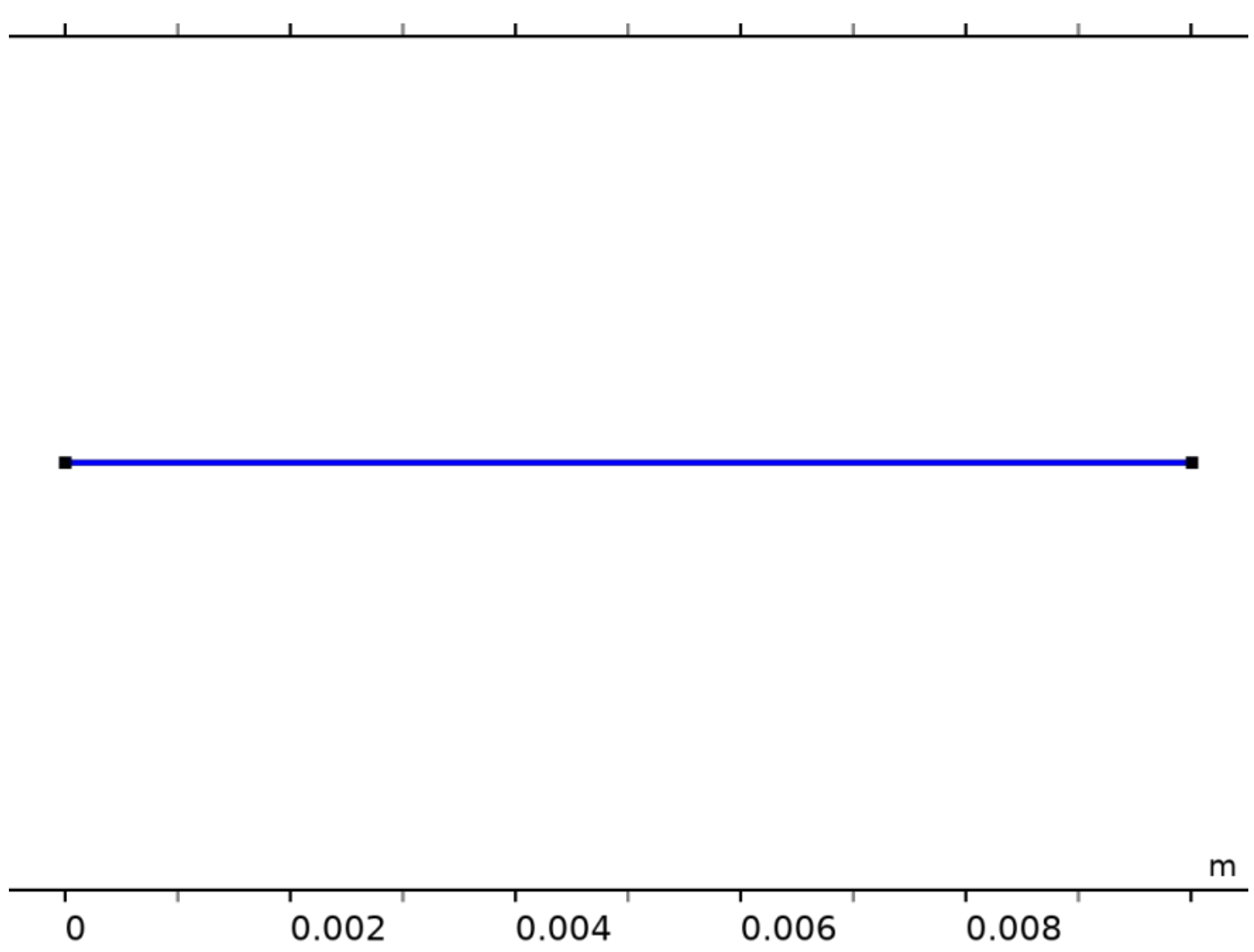

Reactions - Film + Solution

SELECTION

Geometric entity level	Domain
Selection	Geometry geom1: Dimension 1: All domains

EQUATIONS

$$\nabla \cdot \mathbf{J}_i = R_i + S_i$$

Reaction Rates

SETTINGS

Description	Value	Unit
Chemistry	User defined	
Total rate expression	User defined	
Total rate expression	-r_H3PO4	mol/(m³·s)
Total rate expression	User defined	
Total rate expression	-r_H2PO4 + r_H3PO4	mol/(m³·s)
Total rate expression	User defined	
Total rate expression	-r_HPO4 + r_H2PO4	mol/(m³·s)
Total rate expression	User defined	
Total rate expression	r_HPO4	mol/(m³·s)

Heat Source

SETTINGS

Description	Value	Unit
Heat source	User defined	
Heat source	0	W/m³

2.5 POLYMER FILM

USED PRODUCTS

COMSOL Multiphysics
Chemical Reaction Engineering Module

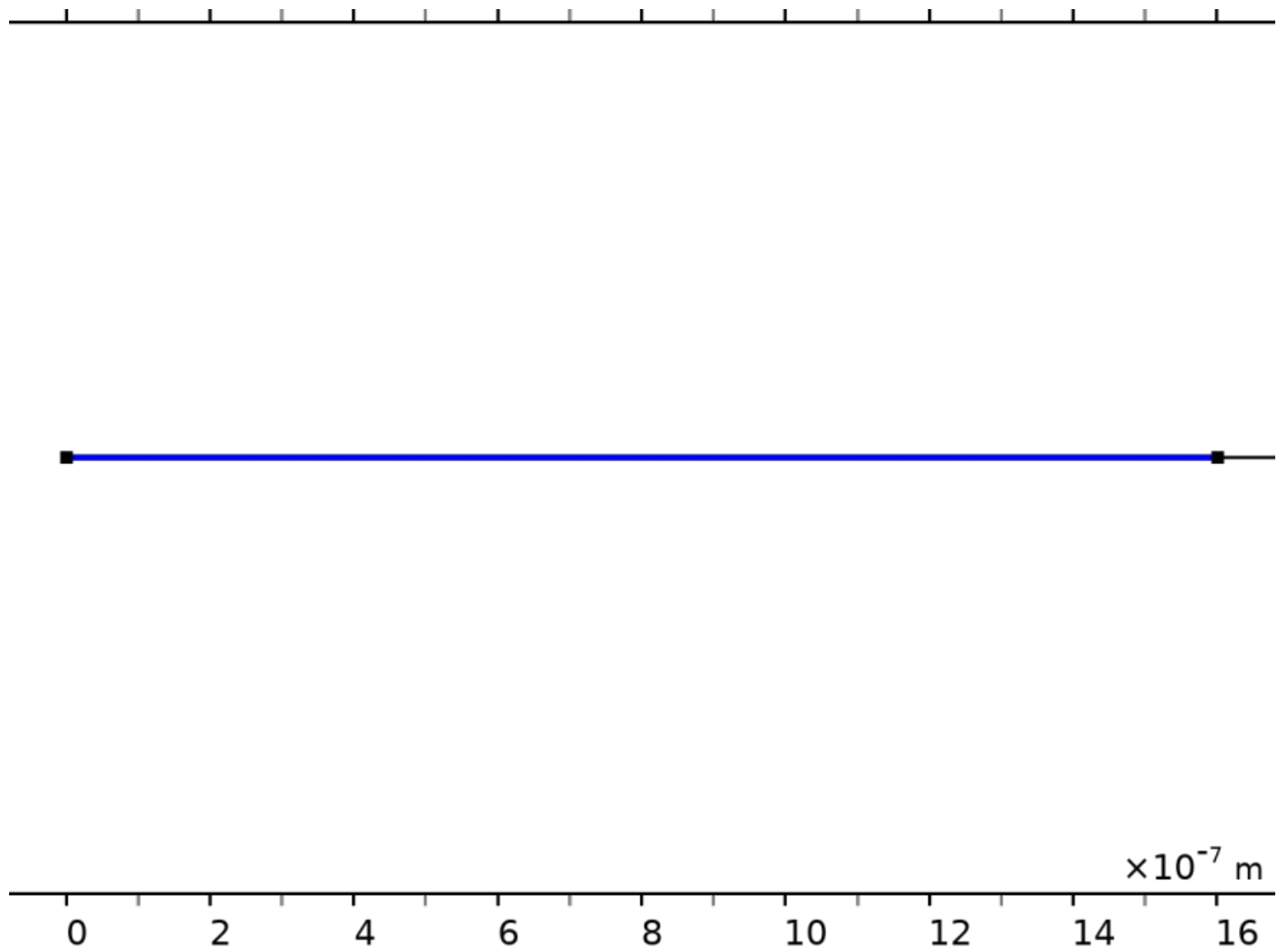

Polymer Film

SELECTION

Geometric entity level	Domain
Selection	Geometry geom1: Dimension 1: Domain 1

EQUATIONS

$$\frac{\partial c_i}{\partial t} + \nabla \cdot \mathbf{J}_i = R_i$$
$$\mathbf{J}_i = -D_i \nabla c_i$$

2.5.1 Interface Settings

Discretization

SETTINGS

Description	Value
Concentration	Linear

SETTINGS

Description	Value
Equation form	Study controlled

Cross-Sectional Area

SETTINGS

Description	Value	Unit
Cross-sectional area	1	m^2

Species Activity

SETTINGS

Description	Value
Species activity	Ideal

Transport Mechanisms

SETTINGS

Description	Value
Convection	Off
Migration in electric field	Off
Mass transfer in porous media	Off

2.5.2 Transport Properties 1

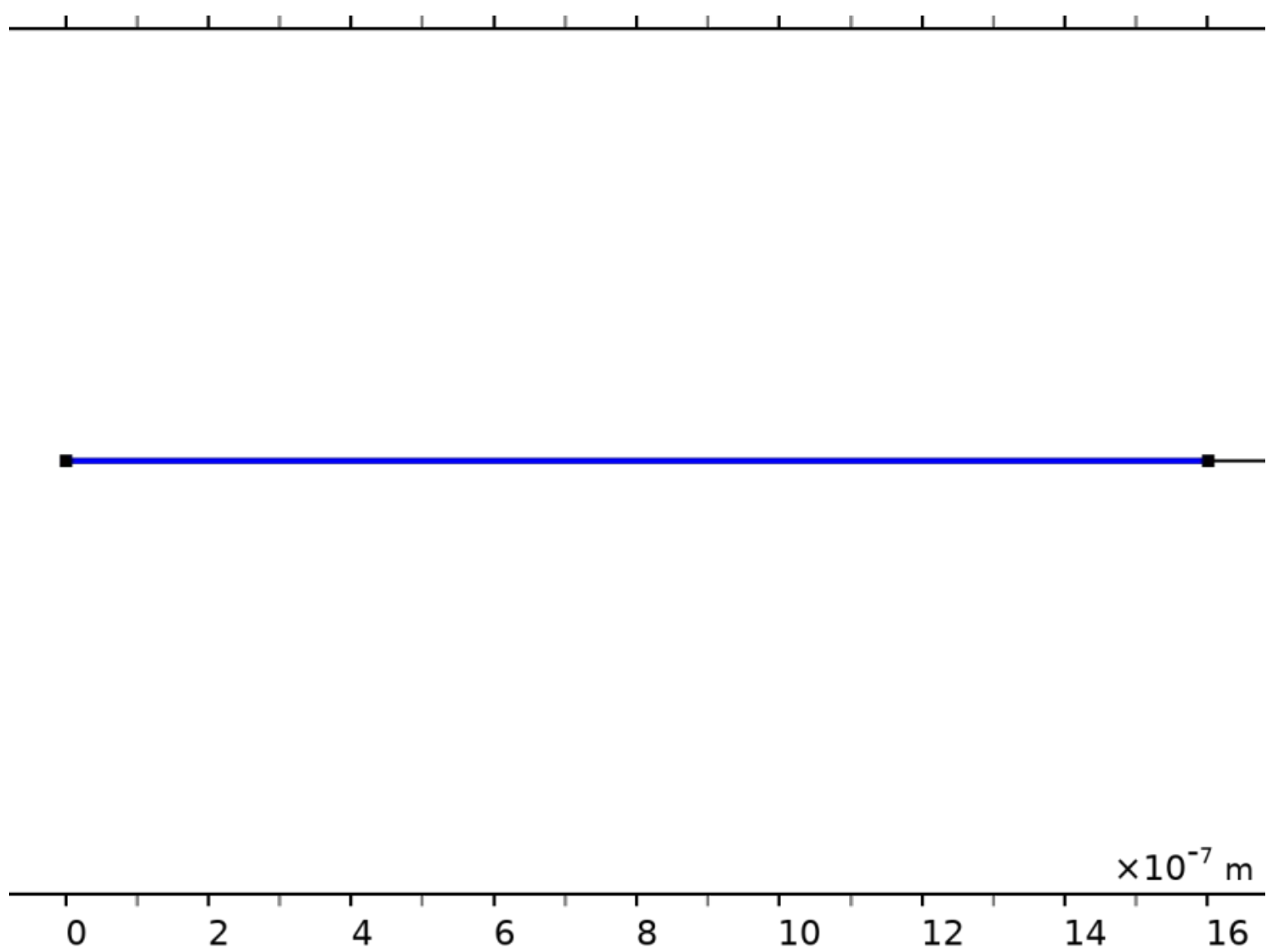

Transport Properties 1

SELECTION

Geometric entity level	Domain
Selection	Geometry geom1: Dimension 1: All domains

EQUATIONS

$$\frac{\partial c_i}{\partial t} + \nabla \cdot \mathbf{J}_i = R_i$$

$$\mathbf{J}_i = -D_i \nabla c_i$$

Diffusion

SETTINGS

Description	Value	Unit
Source	Material	
Material	None	
Diffusion coefficient	User defined	
Diffusion coefficient	0	m^2/s
Diffusion coefficient	User defined	
Diffusion coefficient	0	m^2/s
Diffusion coefficient	User defined	
Diffusion coefficient	0	m^2/s
Diffusion coefficient	User defined	
Diffusion coefficient	0	m^2/s
Diffusion coefficient	User defined	
Diffusion coefficient	0	m^2/s
Diffusion coefficient	User defined	
Diffusion coefficient	0	m^2/s

Coordinate System Selection

SETTINGS

Description	Value
Coordinate system	Global coordinate system

Model Input

SETTINGS

Description	Value	Unit
Temperature	User defined	
Temperature	T	K

2.5.3 No Flux 1

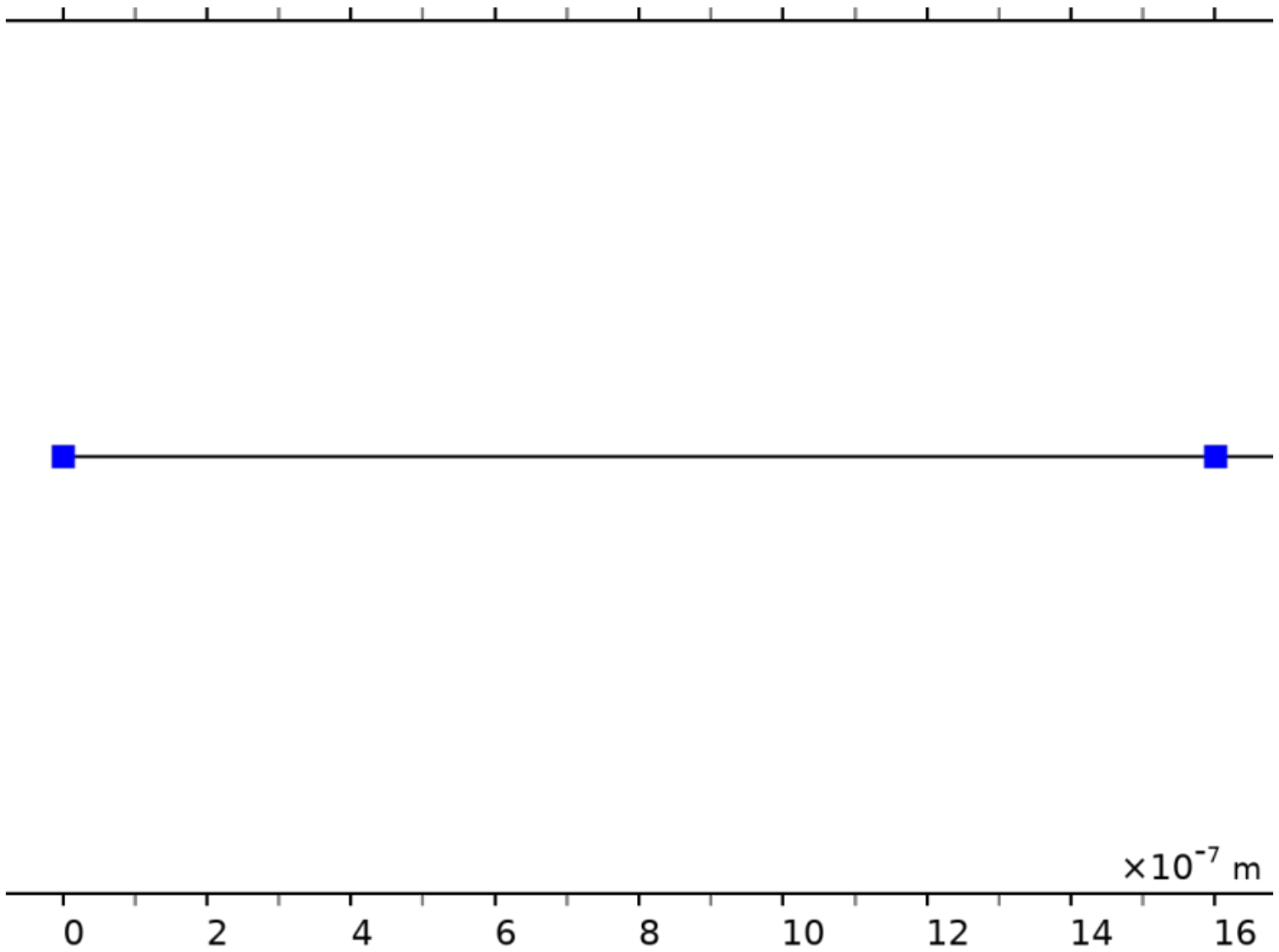

No Flux 1

SELECTION

Geometric entity level	Boundary
Selection	Geometry geom1: Dimension 0: All boundaries

EQUATIONS

$$-\mathbf{n}\cdot\mathbf{J}_i = 0$$

2.5.4 Initial Values 1

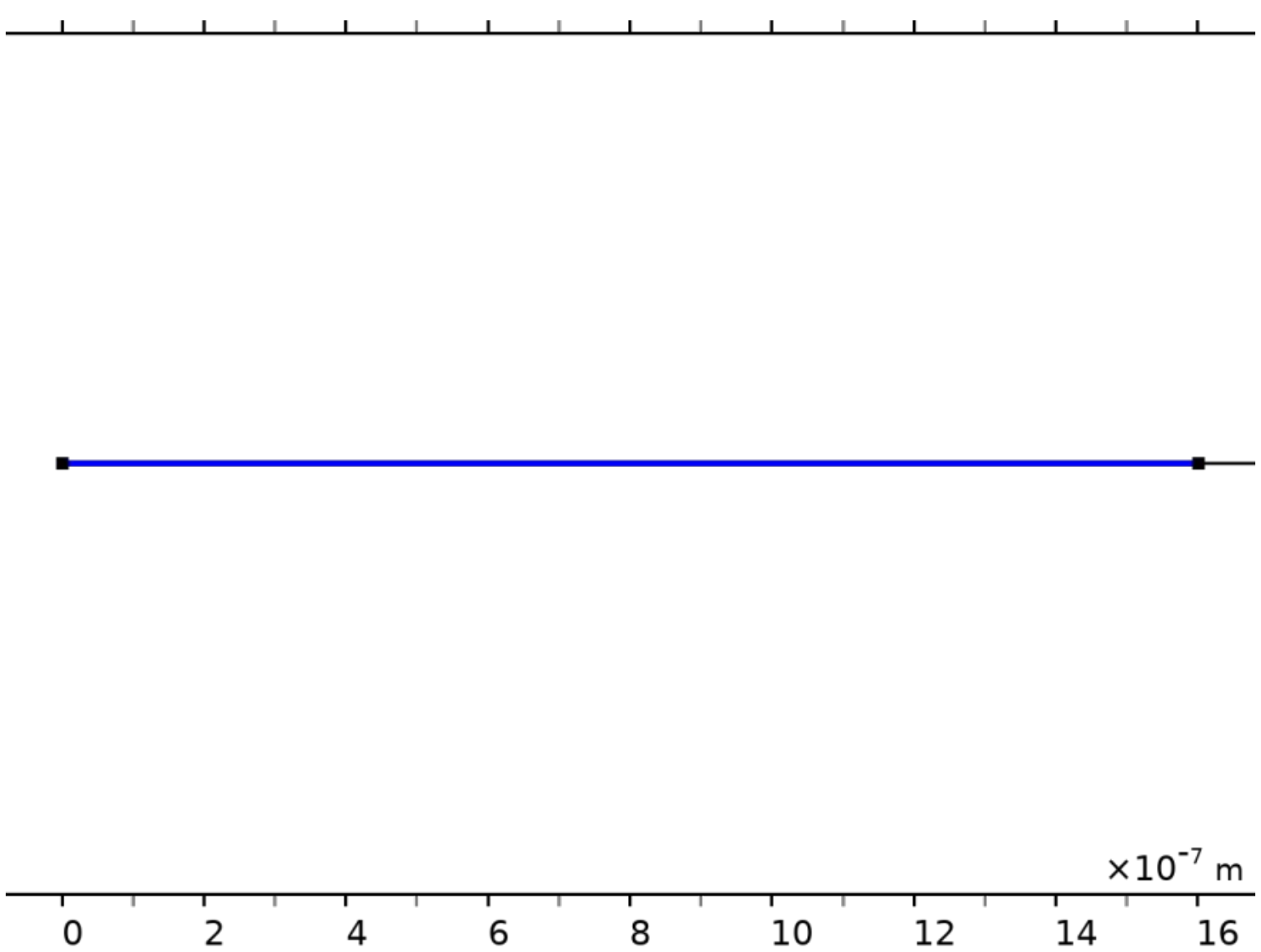

Initial Values 1

SELECTION

Geometric entity level	Domain
Selection	Geometry geom1: Dimension 1: All domains

Initial Values

SETTINGS

**Description**	**Value**	**Unit**
Concentration	cRNH3_p_in	mol/m³
Concentration	cRNH2_dp_in	mol/m³
Concentration	cR2NH2_p_in	mol/m³
Concentration	cR2NH_dp_in	mol/m³
Concentration	cR3NH_p_in	mol/m³
Concentration	cR3N_dp_in	mol/m³

2.5.5 Reactions 2

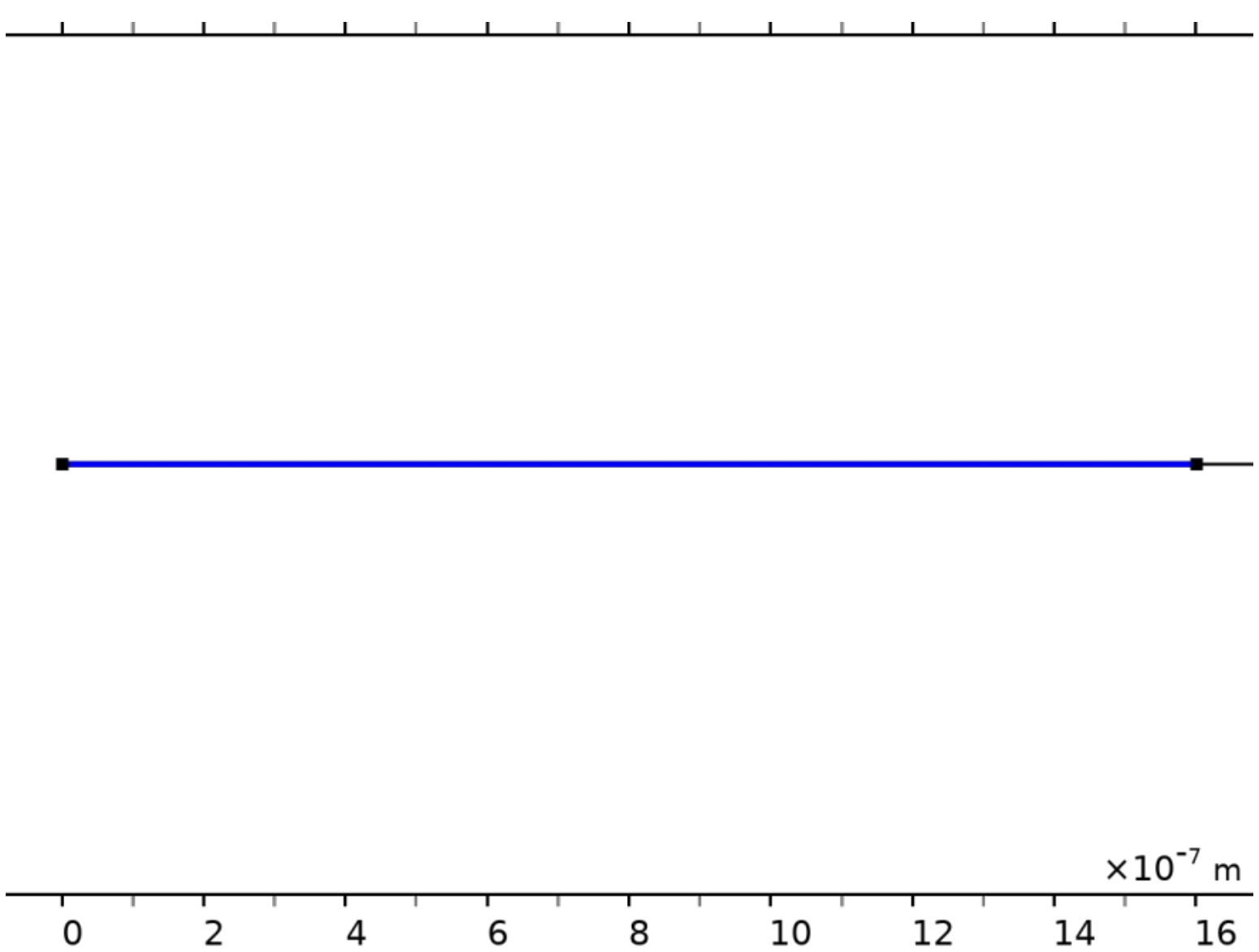

Reactions 2

SELECTION

Geometric entity level	Domain
Selection	Geometry geom1: Dimension 1: Domain 1

EQUATIONS

$$\frac{\partial c_i}{\partial t} + \nabla \cdot \mathbf{J}_i = R_i + S_i$$

Reaction Rates

SETTINGS

Description	Value	Unit
Chemistry	User defined	
Total rate expression	User defined	
Total rate expression	-r_side_chain_primary	mol/(m³·s)
Total rate expression	User defined	
Total rate expression	r_side_chain_primary	mol/(m³·s)
Total rate expression	User defined	
Total rate expression	-r_side_chain_secondary	mol/(m³·s)
Total rate expression	User defined	
Total rate expression	r_side_chain_secondary	mol/(m³·s)
Total rate expression	User defined	
Total rate expression	-r_side_chain_tertiary	mol/(m³·s)
Total rate expression	User defined	
Total rate expression	r_side_chain_tertiary	mol/(m³·s)

Heat Source

SETTINGS

Description	Value	Unit
Heat source	User defined	
Heat source	0	W/m³

2.6 EVENTS

USED PRODUCTS

COMSOL Multiphysics

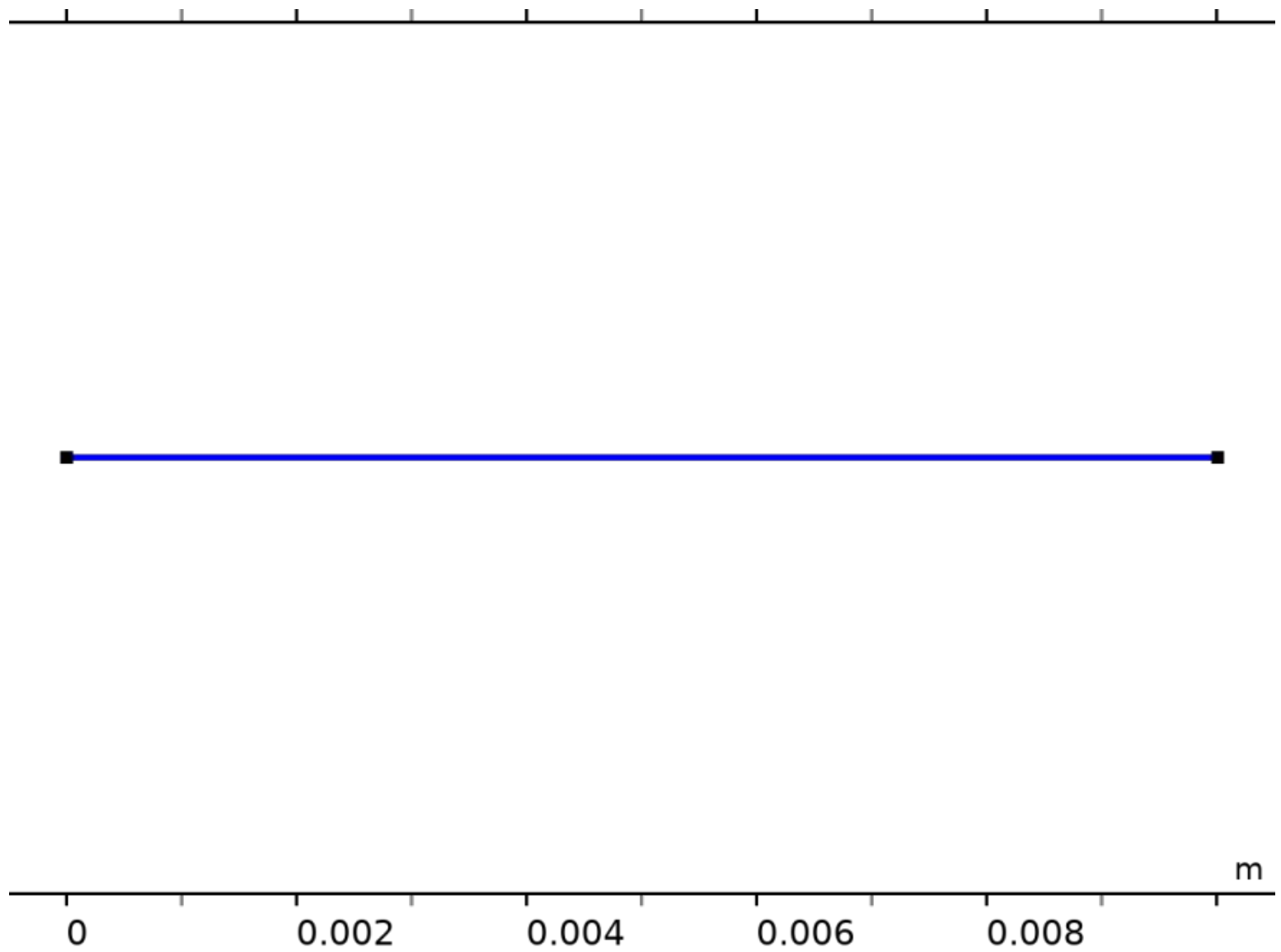

Events

SELECTION

Geometric entity level	Domain
Selection	Geometry geom1: Dimension 1: All domains

2.6.1 Interface Settings

Discretization

SETTINGS

**Description**	**Value**
Element order	Quadratic

SETTINGS

**Description**	**Value**
Equation form	Study controlled

2.6.2 Explicit Event 1

SELECTION

Geometric entity level	Entire model

Event Timings

SETTINGS

**Description**	**Value**	**Unit**
Start of event	0	s
Period of event	Inf	s

Description	Value	Unit
Use consistent initialization	On	

Reinitialization

SETTINGS

Description	Value

2.7 GLOBAL ODES AND DAES

USED PRODUCTS

COMSOL Multiphysics

SELECTION

Geometric entity level	Entire model

2.7.1 Interface Settings

SETTINGS

Description	Value
Equation form	Study controlled

2.7.2 Global Equations 1

SELECTION

Geometric entity level	Entire model

SETTINGS

Description	Value
Field name	ODE1

Global Equations

Name	f(u,ut,utt,t)	Initial value (u_0)	Initial value (ut_0)	Description
V_electrode	(intop1(j_HER*F_const +jC) - j_App(t)) / 1[A/m^2]	E0 + 0.1[mV]	0	

Units

Dependent variable quantity	Unit
none	

Source term quantity	Unit
dimensionless	

2.8 MESH 1

SETTINGS

Description	Value
Type	User - controlled mesh

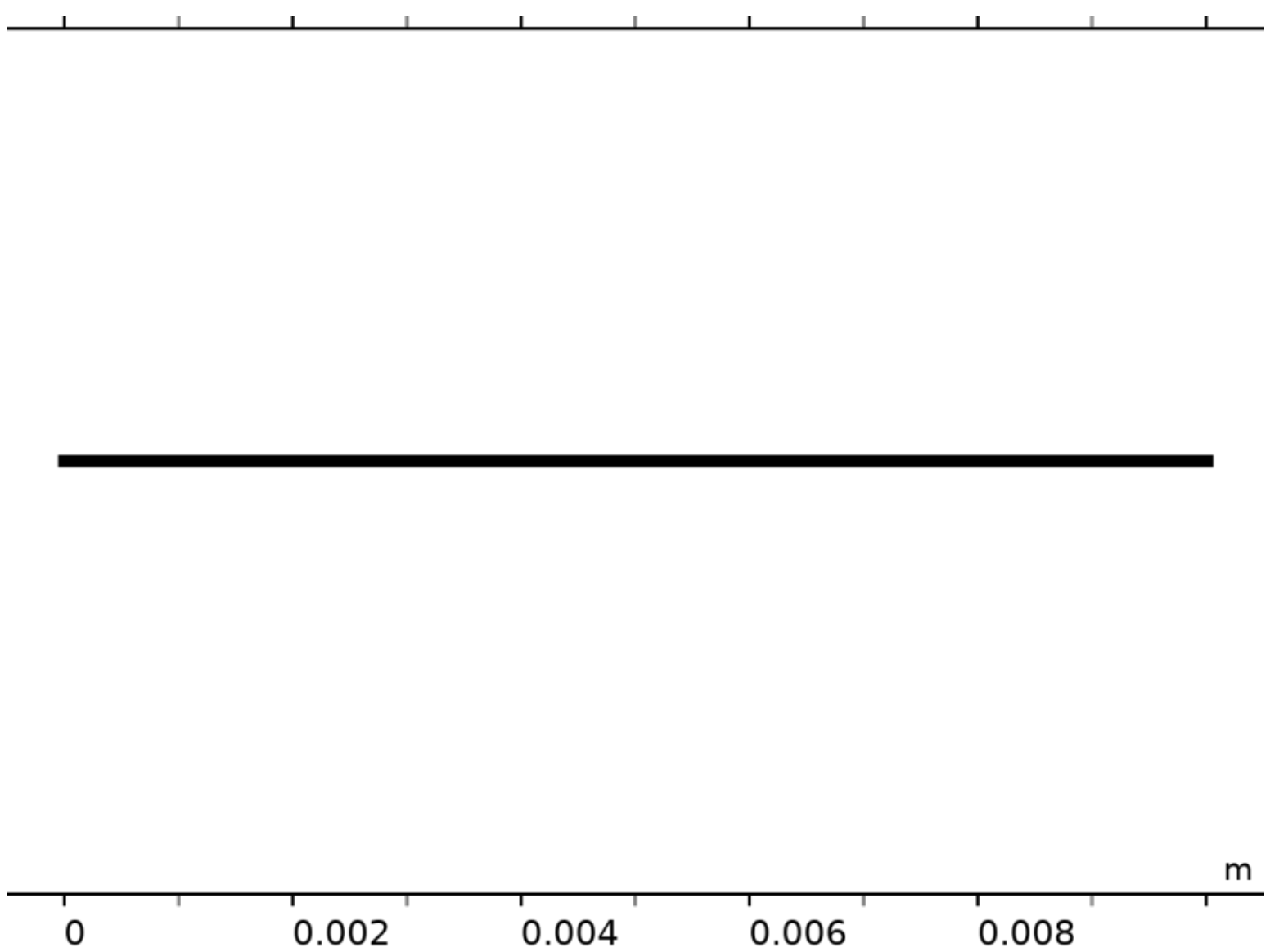

Mesh 1

2.8.1 Size (size)

SETTINGS

Description	Value
Maximum element size	3.7E-4
Minimum element size	1.25E-6
Curvature factor	0.25
Maximum element growth rate	1.25

2.8.2 Edge 1 (edg1)

SELECTION

Geometric entity level	Domain
Selection	Geometry geom1: Dimension 1: Domains 1–2

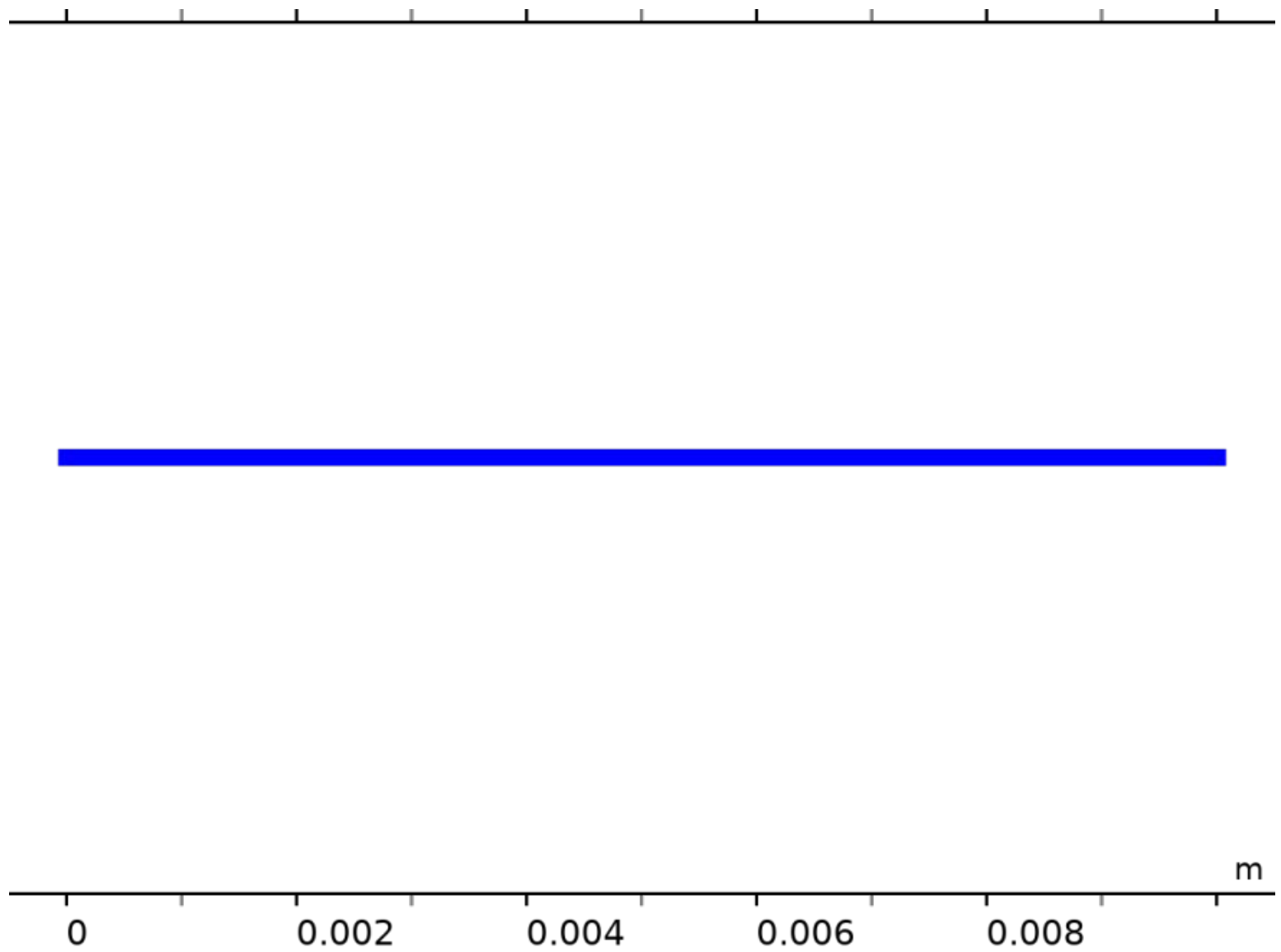

Edge 1

Electrode Surface (size2)

SELECTION

Geometric entity level	Boundary
Selection	Geometry geom1: Dimension 0: Boundary 1

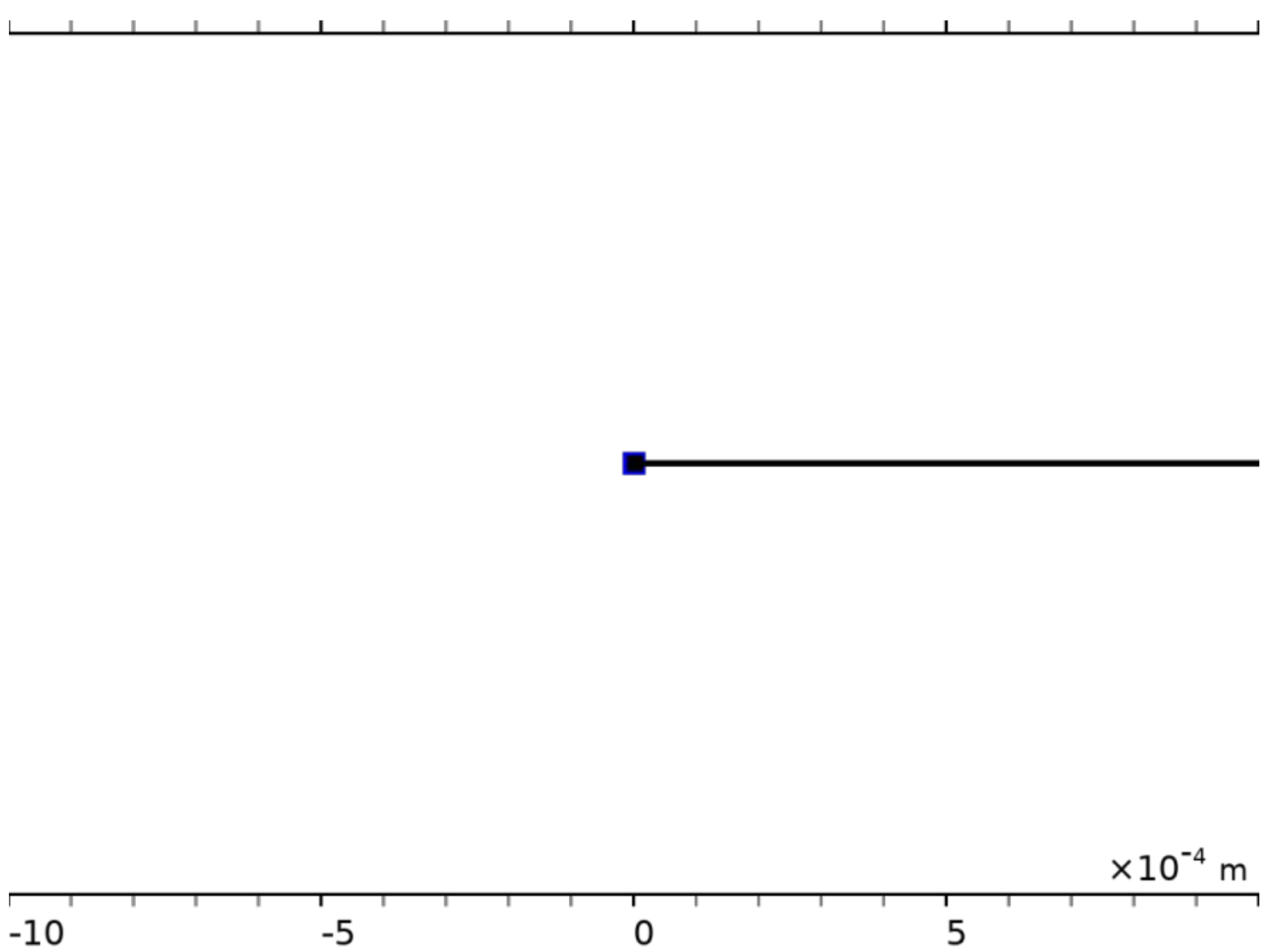

Electrode Surface

SETTINGS

Description	Value
Maximum element size	1[nm]/20

Description	Value
Maximum element growth rate	1.01

Electrolyte (size1)

SELECTION

Geometric entity level	Domain
Selection	Geometry geom1: Dimension 1: Domain 2

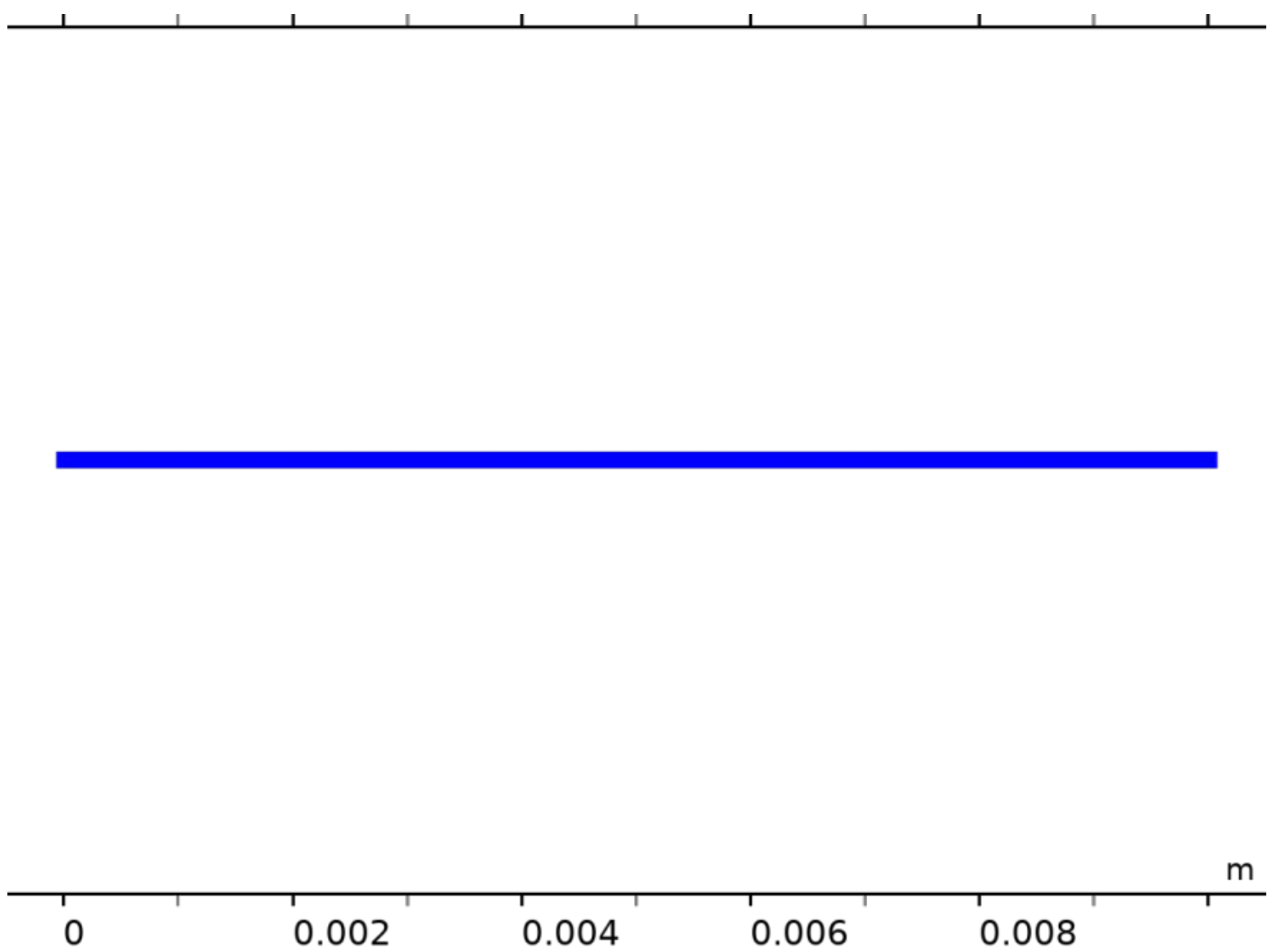

Electrolyte

SETTINGS

Description	Value
Maximum element size	L/100

3 Study 1 - LSV - Potential Sweep

COMPUTATION INFORMATION

Computation time	2 min 45 s

3.1 PARAMETRIC SWEEP - LSV

Parameter name	Parameter value list	Parameter unit
v	0.01	V/s
k_app1	1E-2	cm/s
k_app2	1E-16	cm/s
pH_b	6.2	
cAmine_tot	3.8	mol/m^3
d_film	1.6	um

STUDY SETTINGS

Description	Value
Sweep type	All combinations
Parameter name	{v, k_app1, k_app2, pH_b, cAmine_tot, d_film}
Unit	{V/s, cm/s, cm/s, , mol/m^3, um}

PARAMETERS

Parameter name	Parameter value list	Parameter unit
#v	0.01	V/s
#k_app1 (Electron transfer coeffeient for proton reduction)	0.01	cm/s
#k_app2 (Electron transfer coeffeient for water reduction)	1E-16	cm/s
#pH_b (Bulk pH of the Buffer)	6.2	
#cAmine_tot (Total concentration of amine groups)	3.8	mol/m^3
#d_film (Thickness of polymer layer)	1.6	um

3.2 TIME DEPENDENT

Times	Unit
range(0, Step_time, Scan_Duration[s])	s

STUDY SETTINGS

Description	Value
Include geometric nonlinearity	Off

STORE IN OUTPUT

Interface	Output	Selection
HER (tds3)	Physics controlled	
Phosphate (tds)	Physics controlled	
Polymer Film (tds4)	Physics controlled	
Events (ev)		
Global ODEs and DAEs (ge)		

MESH SELECTION

Component	Mesh
Component 1	Mesh 1

3.3 SOLVER CONFIGURATIONS

3.3.1 Solution 2

Compile Equations: Time Dependent (st1)

STUDY AND STEP

Description	Value
Use study	Study 1 - LSV - Potential Sweep
Use study step	Time Dependent

Dependent Variables 1 (v1)

GENERAL

Description	Value
Defined by study step	Step 1: Time Dependent

RESIDUAL SCALING

Description	Value
Method	Manual

INITIAL VALUE CALCULATION CONSTANTS

Constant name	Initial-value source
t	{range(0, Step_time, Scan_Duration[s])}
timestep	0.1[s]

Concentration (comp1.cH) (comp1_cH)

GENERAL

Description	Value
Field components	comp1.cH

Description	Value
Internal variables	{comp1.uflux.cH, comp1.dflux.cH, comp1.tds3.dt2Inv_cH}

Concentration (comp1.cH2PO4) (comp1_cH2PO4)

GENERAL

Description	Value
Field components	comp1.cH2PO4
Internal variables	{comp1.uflux.cH2PO4, comp1.dflux.cH2PO4, comp1.tds.dt2Inv_cH2PO4}

Concentration (comp1.cH3PO4) (comp1_cH3PO4)

GENERAL

Description	Value
Field components	comp1.cH3PO4
Internal variables	{comp1.uflux.cH3PO4, comp1.dflux.cH3PO4, comp1.tds.dt2Inv_cH3PO4}

Concentration (comp1.cHPO4) (comp1_cHPO4)

GENERAL

Description	Value
Field components	comp1.cHPO4
Internal variables	{comp1.uflux.cHPO4, comp1.dflux.cHPO4, comp1.tds.dt2Inv_cHPO4}

Concentration (comp1.cR2NH) (comp1_cR2NH)

GENERAL

Description	Value
Field components	comp1.cR2NH
Internal variables	{comp1.uflux.cR2NH, comp1.dflux.cR2NH, comp1.tds4.dt2Inv_cR2NH}

Concentration (comp1.cR2NH2) (comp1_cR2NH2)

GENERAL

Description	Value
Field components	comp1.cR2NH2
Internal variables	{comp1.uflux.cR2NH2, comp1.dflux.cR2NH2, comp1.tds4.dt2Inv_cR2NH2}

Concentration (comp1.cOH) (comp1_cOH)

GENERAL

Description	Value
Field components	comp1.cOH
Internal variables	{comp1.uflux.cOH, comp1.dflux.cOH, comp1.tds3.dt2Inv_cOH}

Concentration (comp1.cPO4) (comp1_cPO4)

GENERAL

Description	Value
Field components	comp1.cPO4
Internal variables	{comp1.uflux.cPO4, comp1.dflux.cPO4, comp1.tds.dt2Inv_cPO4}

Concentration (comp1.cRNH3) (comp1_cRNH3)

GENERAL

Description	Value
Field components	comp1.cRNH3
Internal variables	{comp1.uflux.cRNH3, comp1.dflux.cRNH3, comp1.tds4.dt2Inv_cRNH3}

Concentration (comp1.cRNH2) (comp1_cRNH2)

GENERAL

Description	Value
Field components	comp1.cRNH2
Internal variables	{comp1.uflux.cRNH2, comp1.dflux.cRNH2, comp1.tds4.dt2Inv_cRNH2}

Global Equations 1 (comp1.ODE1) (comp1_ODE1)

GENERAL

Description	Value
State components	comp1.V_electrode

Concentration (comp1.cR3_N) (comp1_cR3_N)

GENERAL

Description	Value
Field components	comp1.cR3_N
Internal variables	{comp1.uflux.cR3_N, comp1.dflux.cR3_N, comp1.tds4.dt2Inv_cR3_N}

Concentration (comp1.cR3_NH) (comp1_cR3_NH)

GENERAL

Description	Value
Field components	comp1.cR3_NH
Internal variables	{comp1.uflux.cR3_NH, comp1.dflux.cR3_NH, comp1.tds4.dt2Inv_cR3_NH}

Time-Dependent Solver 1 (t1)

GENERAL

Description	Value
Defined by study step	Step 1: Time Dependent

Description	Value
Relative tolerance	0.001

ABSOLUTE TOLERANCE

Field	Method	Tolerance method	Tolerance factor	Derivative tolerance method	Time derivative factor	Tolerance	Tolerance for time derivatives
Concentration (comp1.cH)	Use global	Factor	0.1	Automatic	1	0.001	0.001
Concentration (comp1.cH2PO4)	Use global	Factor	0.1	Automatic	1	0.001	0.001
Concentration (comp1.cH3PO4)	Use global	Factor	0.1	Automatic	1	0.001	0.001
Concentration (comp1.cHPO4)	Use global	Factor	0.1	Automatic	1	0.001	0.001
Concentration (comp1.cR2NH)	Use global	Factor	0.1	Automatic	1	0.001	0.001
Concentration (comp1.cR2NH2)	Use global	Factor	0.1	Automatic	1	0.001	0.001
Concentration (comp1.cOH)	Use global	Factor	0.1	Automatic	1	0.001	0.001
Concentration (comp1.cPO4)	Use global	Factor	0.1	Automatic	1	0.001	0.001
Concentration (comp1.cRNH3)	Use global	Factor	0.1	Automatic	1	0.001	0.001

Field	Method	Tolerance method	Tolerance factor	Derivative tolerance method	Time derivative factor	Tolerance	Tolerance for time derivatives
Concentration (comp1.cRNH2)	Use global	Factor	0.1	Automatic	1	0.001	0.001
Global Equations 1 (comp1.ODE1)	Use global	Factor	0.1	Automatic	1	0.001	0.001
Concentration (comp1.cR3_N)	Use global	Factor	0.1	Automatic	1	0.001	0.001
Concentration (comp1.cR3_NH)	Use global	Factor	0.1	Automatic	1	0.001	0.001

TIME STEPPING

Description	Value
Steps taken by solver	Intermediate
Maximum BDF order	2
Nonlinear controller	On

RESULTS WHILE SOLVING

Description	Value
Plot	On

RESULTS WHILE SOLVING

Plot group	Plot window
#Study 3 - CP - pH in film - Plot During Simulation	Graphics

Advanced (aDef)

ASSEMBLY SETTINGS

Description	Value
Reuse sparsity pattern	On

Fully Coupled 1 (fc1)

GENERAL

Description	Value
Linear solver	Direct, concentrations (tds) (Merged)

METHOD AND TERMINATION

Description	Value
Nonlinear method	Automatic highly nonlinear (Newton)
Maximum number of iterations	8

Direct, concentrations (tds) (Merged) (d1)

GENERAL

Description	Value
Solver	PARDISO
Pivoting perturbation	1E-13

3.3.2 Parametric Solutions 2

v=0.01, k_app1=0.01, k_app2=1E-16, pH_b=6.2, cAmine_tot=3.8, d_film=1.6 (su1)

GENERAL

Description	Value
Solution	v=0.01, k_app1=0.01, k_app2=1E-16, pH_b=6.2, cAmine_tot=3.8, d_film=1.6 (sol53)

4 Study 2 - CA - Constant Potential

COMPUTATION INFORMATION

Computation time	7 min 49 s

4.1 PARAMETRIC SWEEP

Parameter name	Parameter value list	Parameter unit
E_App	-0.4, -0.6	V
k_app1	1E-4, 0.1	cm/s

STUDY SETTINGS

Description	Value
Sweep type	All combinations
Parameter name	{E_App, k_app1}
Unit	{V, cm/s}

PARAMETERS

Parameter name	Parameter value list	Parameter unit
#E_App (CA - Applied Potential)	-0.4, -0.6	V
#k_app1 (Electron transfer coeffeient for proton reduction)	1E-4, 0.1	cm/s

4.2 TIME DEPENDENT

Times	Unit
range(-10,1,-1.1 [s]), range(-1,0.01,1 [s]), range(2,2,200 [s])	s

STUDY SETTINGS

Description	Value
Include geometric nonlinearity	Off

STUDY SETTINGS

Description	Value
Tolerance	User controlled
Relative tolerance	1E-4

STORE IN OUTPUT

Interface	Output	Selection
HER (tds3)	Physics controlled	
Phosphate (tds)	Physics controlled	
Polymer Film (tds4)	Physics controlled	

Interface	Output	Selection
Events (ev)		
Global ODEs and DAEs (ge)		

MESH SELECTION

Component	Mesh
Component 1	Mesh 1

4.3 SOLVER CONFIGURATIONS

4.3.1 Solution 8

Compile Equations: Time Dependent (st1)

STUDY AND STEP

Description	Value
Use study	Study 2 - CA - Constant Potential
Use study step	Time Dependent

Dependent Variables 1 (v1)

GENERAL

Description	Value
Defined by study step	Step 1: Time Dependent

RESIDUAL SCALING

Description	Value
Method	Manual

INITIAL VALUE CALCULATION CONSTANTS

Constant name	Initial-value source
t	{range(-10,1,-1.1 [s]), range(-1,0.01,1 [s]), range(2,2,200 [s])}[s]
timestep	0.21[s]

Concentration (comp1.cCat) (comp1_cCat)

GENERAL

Description	Value
Field components	comp1.cCat
Internal variables	{comp1.uflux.cCat, comp1.dflux.cCat, comp1.tds.dt2Inv_cCat}

Concentration (comp1.cH) (comp1_cH)

GENERAL

Description	Value
Field components	comp1.cH
Internal variables	{comp1.uflux.cH, comp1.dflux.cH, comp1.tds3.dt2Inv_cH}

Concentration (comp1.cH2) (comp1_cH2)

GENERAL

Description	Value
Field components	comp1.cH2
Internal variables	{comp1.uflux.cH2, comp1.dflux.cH2, comp1.tds3.dt2Inv_cH2}

Concentration (comp1.cH2O) (comp1_cH2O)

GENERAL

Description	Value
Field components	comp1.cH2O
Internal variables	{comp1.uflux.cH2O, comp1.dflux.cH2O, comp1.tds3.dt2Inv_cH2O}

Concentration (comp1.cH2PO4) (comp1_cH2PO4)

GENERAL

Description	Value
Field components	comp1.cH2PO4
Internal variables	{comp1.uflux.cH2PO4, comp1.dflux.cH2PO4, comp1.tds.dt2Inv_cH2PO4}

Concentration (comp1.cH3PO4) (comp1_cH3PO4)

GENERAL

Description	Value
Field components	comp1.cH3PO4
Internal variables	{comp1.uflux.cH3PO4, comp1.dflux.cH3PO4, comp1.tds.dt2Inv_cH3PO4}

Concentration (comp1.cHPO4) (comp1_cHPO4)

GENERAL

Description	Value
Field components	comp1.cHPO4
Internal variables	{comp1.uflux.cHPO4, comp1.dflux.cHPO4, comp1.tds.dt2Inv_cHPO4}

Concentration (comp1.cNH2) (comp1_cNH2)

GENERAL

Description	Value
Field components	comp1.cNH2
Internal variables	{comp1.uflux.cNH2, comp1.dflux.cNH2, comp1.tds4.dt2Inv_cNH2}

Concentration (comp1.cNH3) (comp1_cNH3)

GENERAL

Description	Value
Field components	comp1.cNH3
Internal variables	{comp1.uflux.cNH3, comp1.dflux.cNH3, comp1.tds4.dt2Inv_cNH3}

Concentration (comp1.cOH) (comp1_cOH)

GENERAL

Description	Value
Field components	comp1.cOH
Internal variables	{comp1.uflux.cOH, comp1.dflux.cOH, comp1.tds3.dt2Inv_cOH}

Concentration (comp1.cPO4) (comp1_cPO4)

GENERAL

Description	Value
Field components	comp1.cPO4
Internal variables	{comp1.uflux.cPO4, comp1.dflux.cPO4, comp1.tds.dt2Inv_cPO4}

Concentration (comp1.cR2_N) (comp1_cR2_N)

GENERAL

Description	Value
Field components	comp1.cR2_N
Internal variables	{comp1.uflux.cR2_N, comp1.dflux.cR2_N, comp1.tds4.dt2Inv_cR2_N}

Concentration (comp1.cR2_NH) (comp1_cR2_NH)

GENERAL

Description	Value
Field components	comp1.cR2_NH
Internal variables	{comp1.uflux.cR2_NH, comp1.dflux.cR2_NH, comp1.tds4.dt2Inv_cR2_NH}

Concentration (comp1.cRNH) (comp1_cRNH)

GENERAL

Description	Value
Field components	comp1.cRNH
Internal variables	{comp1.uflux.cRNH, comp1.dflux.cRNH, comp1.tds4.dt2Inv_cRNH}

Concentration (comp1.cRNH2) (comp1_cRNH2)

GENERAL

Description	Value
Field components	comp1.cRNH2
Internal variables	{comp1.uflux.cRNH2, comp1.dflux.cRNH2, comp1.tds4.dt2Inv_cRNH2}

Time-Dependent Solver 1 (t1)

GENERAL

Description	Value
Defined by study step	Step 1: Time Dependent
Relative tolerance	1E-4

ABSOLUTE TOLERANCE

Field	Method	Tolerance method	Tolerance factor	Derivative tolerance method	Time derivative factor	Tolerance	Tolerance for time derivatives
Concentration (comp1.cCat)	Use global	Factor	0.1	Automatic	1	0.001	0.001
Concentration (comp1.cH)	Use global	Factor	0.1	Automatic	1	0.001	0.001
Concentration (comp1.cH2)	Use global	Factor	0.1	Automatic	1	0.001	0.001
Concentration (comp1.cH2O)	Use global	Factor	0.1	Automatic	1	0.001	0.001
Concentration (comp1.cH2PO4)	Use global	Factor	0.1	Automatic	1	0.001	0.001
Concentration (comp1.cH3PO4)	Use global	Factor	0.1	Automatic	1	0.001	0.001
Concentration (comp1.cHPO4)	Use global	Factor	0.1	Automatic	1	0.001	0.001

Field	Method	Tolerance method	Tolerance factor	Derivative tolerance method	Time derivative factor	Tolerance	Tolerance for time derivatives
Concentration (comp1.cNH2)	Use global	Factor	0.1	Automatic	1	0.001	0.001
Concentration (comp1.cNH3)	Use global	Factor	0.1	Automatic	1	0.001	0.001
Concentration (comp1.cOH)	Use global	Factor	0.1	Automatic	1	0.001	0.001
Concentration (comp1.cPO4)	Use global	Factor	0.1	Automatic	1	0.001	0.001
Concentration (comp1.cR2_N)	Use global	Factor	0.1	Automatic	1	0.001	0.001
Concentration (comp1.cR2_NH)	Use global	Factor	0.1	Automatic	1	0.001	0.001
Concentration (comp1.cRNH)	Use global	Factor	0.1	Automatic	1	0.001	0.001
Concentration (comp1.cRNH2)	Use global	Factor	0.1	Automatic	1	0.001	0.001

TIME STEPPING

Description	Value
Steps taken by solver	Intermediate
Maximum BDF order	2
Nonlinear controller	On

Advanced (aDef)

ASSEMBLY SETTINGS

Description	Value
Reuse sparsity pattern	On

Fully Coupled 1 (fc1)

GENERAL

Description	Value
Linear solver	Direct, concentrations (tds) (Merged)

METHOD AND TERMINATION

Description	Value
Nonlinear method	Automatic (Newton)
Maximum number of iterations	8

Direct, concentrations (tds) (Merged) (d1)

GENERAL

Description	Value
Solver	PARDISO
Pivoting perturbation	1E-13

4.3.2 Parametric Solutions 3

E_App=-0.4, k_app1=1E-4 (su1)

GENERAL

Description	Value
Solution	E_App= - 0.4, k_app1=1E-4 (sol18)

E_App=-0.4, k_app1=0.1 (su2)

GENERAL

Description	Value
Solution	E_App= - 0.4, k_app1=0.1 (sol19)

E_App=-0.6, k_app1=1E-4 (su3)

GENERAL

Description	Value
Solution	E_App= - 0.6, k_app1=1E-4 (sol20)

E_App=-0.6, k_app1=0.1 (su4)

GENERAL

Description	Value
Solution	E_App= - 0.6, k_app1=0.1 (sol21)

5 Study 3 - CP - Constant Current

COMPUTATION INFORMATION

Computation time	2 min 20 s

5.1 PARAMETRIC SWEEP

Parameter name	Parameter value list	Parameter unit
k_app1	0.01	cm/s
pH_b	4.5	
j_pulse	-0.125, -0.25, -0.5	mA/cm^2
pKa6	7	
d_film	1.6	um
cAmine_tot	0, 3.8	M
Film_Phosphate_Scale	1	
Film_H_OH_Scale	1	
k_b1	5E10	M^-1*s^-1
Cdl	20	uF/cm^2

STUDY SETTINGS

Description	Value
Sweep type	All combinations
Parameter name	{k_app1, pH_b, j_pulse, pKa6, d_film, cAmine_tot, Film_Phosphate_Scale, Film_H_OH_Scale, k_b1, Cdl}
Unit	{cm/s, , mA/cm^2, , um, M, , , M^ - 1*s^ - 1, uF/cm^2}

PARAMETERS

Parameter name	Parameter value list	Parameter unit
#k_app1 (Electron transfer coeffeient for proton reduction)	0.01	cm/s
#pH_b (Bulk pH of the Buffer)	4.5	
#j_pulse	-0.125, -0.25, -0.5	mA/cm^2
#pKa6 (pKa of the secondary amine)	7	
#d_film (Thickness of polymer layer)	1.6	um
#cAmine_tot (Total concentration of amine groups)	0, 3.8	M
#Film_Phosphate_Scale (Used to reduce the diffusion coeffient of all 4 phosphate species by the same scaling)	1	

Parameter name	Parameter value list	Parameter unit
#Film_H_OH_Scale (Used to reduce the diffusion coeffient of all H+ and OH- species by the same scaling)	1	
#k_b1 (Assumed to be in the range 10^10 - 10^11 [M^-1 s^-1])	5E10	M^-1*s^-1
#Cdl (This is arbitary but needed to stabalise the feedback loop)	20	uF/cm^2

5.2 TIME DEPENDENT

Times	Unit
range(0,0.05,1 [s]), range(2,2,120 [s])	s

STUDY SETTINGS

Description	Value
Include geometric nonlinearity	Off

STUDY SETTINGS

Description	Value
Tolerance	User controlled
Relative tolerance	0.001

STORE IN OUTPUT

Interface	Output	Selection
HER (tds3)	Physics controlled	
Phosphate (tds)	Physics controlled	
Polymer Film (tds4)	Physics controlled	
Events (ev)		
Global ODEs and DAEs (ge)		

MESH SELECTION

Component	Mesh
Component 1	Mesh 1

5.3 SOLVER CONFIGURATIONS

5.3.1 Solution 22

Compile Equations: Time Dependent (st1)

STUDY AND STEP

Description	Value
Use study	Study 3 - CP - Constant Current
Use study step	Time Dependent

Dependent Variables 1 (v1)

GENERAL

Description	Value
Defined by study step	Step 1: Time Dependent

INITIAL VALUE CALCULATION CONSTANTS

Constant name	Initial-value source
t	{range(0[s], 0.05[s], 1[s]), range(2[s], 2[s], 120[s])}
timestep	0.12[s]

Concentration (comp1.cH) (comp1_cH)

GENERAL

Description	Value
Field components	comp1.cH
Internal variables	{comp1.uflux.cH, comp1.dflux.cH, comp1.tds3.dt2Inv_cH}

Concentration (comp1.cH2PO4) (comp1_cH2PO4)

GENERAL

Description	Value
Field components	comp1.cH2PO4
Internal variables	{comp1.uflux.cH2PO4, comp1.dflux.cH2PO4, comp1.tds.dt2Inv_cH2PO4}

Concentration (comp1.cH3PO4) (comp1_cH3PO4)

GENERAL

Description	Value
Field components	comp1.cH3PO4
Internal variables	{comp1.uflux.cH3PO4, comp1.dflux.cH3PO4, comp1.tds.dt2Inv_cH3PO4}

Concentration (comp1.cHPO4) (comp1_cHPO4)

GENERAL

Description	Value
Field components	comp1.cHPO4
Internal variables	{comp1.uflux.cHPO4, comp1.dflux.cHPO4, comp1.tds.dt2Inv_cHPO4}

Concentration (comp1.cOH) (comp1_cOH)

GENERAL

Description	Value
Field components	comp1.cOH
Internal variables	{comp1.uflux.cOH, comp1.dflux.cOH, comp1.tds3.dt2Inv_cOH}

Concentration (comp1.cPO4) (comp1_cPO4)

GENERAL

Description	Value
Field components	comp1.cPO4
Internal variables	{comp1.uflux.cPO4, comp1.dflux.cPO4, comp1.tds.dt2Inv_cPO4}

Concentration (comp1.cR2NH) (comp1_cR2NH)

GENERAL

Description	Value
Field components	comp1.cR2NH
Internal variables	{comp1.uflux.cR2NH, comp1.dflux.cR2NH, comp1.tds4.dt2Inv_cR2NH}

Concentration (comp1.cR2NH2) (comp1_cR2NH2)

GENERAL

Description	Value
Field components	comp1.cR2NH2
Internal variables	{comp1.uflux.cR2NH2, comp1.dflux.cR2NH2, comp1.tds4.dt2Inv_cR2NH2}

Concentration (comp1.cRNH2) (comp1_cRNH2)

GENERAL

Description	Value
Field components	comp1.cRNH2
Internal variables	{comp1.uflux.cRNH2, comp1.dflux.cRNH2, comp1.tds4.dt2Inv_cRNH2}

Concentration (comp1.cRNH3) (comp1_cRNH3)

GENERAL

Description	Value
Field components	comp1.cRNH3
Internal variables	{comp1.uflux.cRNH3, comp1.dflux.cRNH3, comp1.tds4.dt2Inv_cRNH3}

Global Equations 1 (comp1.ODE1) (comp1_ODE1)

GENERAL

Description	Value
State components	comp1.V_electrode

Concentration (comp1.cR3_N) (comp1_cR3_N)

GENERAL

Description	Value
Field components	comp1.cR3_N
Internal variables	{comp1.uflux.cR3_N, comp1.dflux.cR3_N, comp1.tds4.dt2Inv_cR3_N}

Concentration (comp1.cR3_NH) (comp1_cR3_NH)

GENERAL

Description	Value
Field components	comp1.cR3_NH
Internal variables	{comp1.uflux.cR3_NH, comp1.dflux.cR3_NH, comp1.tds4.dt2Inv_cR3_NH}

Time-Dependent Solver 1 (t1)

GENERAL

Description	Value
Defined by study step	Step 1: Time Dependent
Output times	{0, 0.05, 0.1, 0.15, 0.2, 0.25, 0.3, 0.35, 0.4, 0.45, 0.5, 0.55, 0.6, 0.65, 0.7, 0.75, 0.8, 0.85, 0.9, 0.95, 1, 2, 4, 6, 8, 10, 12, 14, 16, 18, 20, 22, 24, 26, 28, 30, 32, 34, 36, 38, 40, 42, 44, 46, 48, 50, 52, 54, 56, 58, 60, 62, 64, 66, 68, 70, 72, 74, 76, 78, 80, 82, 84, 86, 88, 90, 92, 94, 96, 98, 100, 102, 104, 106, 108, 110, 112, 114, 116, 118, 120}
Relative tolerance	0.001

ABSOLUTE TOLERANCE

Field	Method	Tolerance method	Tolerance factor	Derivative tolerance method	Time derivative factor	Tolerance	Tolerance for time derivatives
Concentration (comp1.cH)	Use global	Factor	0.1	Automatic	1	0.001	0.001
Concentration (comp1.cH2PO4)	Use global	Factor	0.1	Automatic	1	0.001	0.001
Concentration	Use global	Factor	0.1	Automatic	1	0.001	0.001

Field	Method	Tolerance method	Tolerance factor	Derivative tolerance method	Time derivative factor	Tolerance	Tolerance for time derivatives
(comp1.cH3PO4)							
Concentration (comp1.cHPO4)	Use global	Factor	0.1	Automatic	1	0.001	0.001
Concentration (comp1.cOH)	Use global	Factor	0.1	Automatic	1	0.001	0.001
Concentration (comp1.cPO4)	Use global	Factor	0.1	Automatic	1	0.001	0.001
Concentration (comp1.cR2NH)	Use global	Factor	0.1	Automatic	1	0.001	0.001
Concentration (comp1.cR2NH2)	Use global	Factor	0.1	Automatic	1	0.001	0.001
Concentration (comp1.cRNH2)	Use global	Factor	0.1	Automatic	1	0.001	0.001
Concentration (comp1.cRNH3)	Use global	Factor	0.1	Automatic	1	0.001	0.001
Global Equations 1 (comp1.ODE1)	Use global	Factor	0.1	Automatic	1	0.001	0.001
Concentration (comp1.cR3_N)	Use global	Factor	0.1	Automatic	1	0.001	0.001
Concentration	Use global	Factor	0.1	Automatic	1	0.001	0.001

Field	Method	Tolerance method	Tolerance factor	Derivative tolerance method	Time derivative factor	Tolerance	Tolerance for time derivatives
(comp1.cR3_NH)							

TIME STEPPING

Description	Value
Steps taken by solver	Intermediate
Initial step	1E-9
Maximum BDF order	2
Nonlinear controller	On

RESULTS WHILE SOLVING

Description	Value
Plot	On

RESULTS WHILE SOLVING

Plot group	Plot window
#Study 3 - CP - pH in film - Plot During Simulation	Graphics

Direct (dDef)

GENERAL

Description	Value
Solver	PARDISO

Advanced (aDef)

ASSEMBLY SETTINGS

Description	Value
Reuse sparsity pattern	On

Fully Coupled 1 (fc1)

GENERAL

Description	Value
Linear solver	Direct

METHOD AND TERMINATION

Description	Value
Damping factor	0.9
Jacobian update	Once per time step

Description	Value
Maximum number of iterations	8
Stabilization and acceleration	Anderson acceleration
Dimension of iteration space	5
Mixing parameter	0.9

5.3.2 Parametric Solutions 4

k_app1=0.01, pH_b=4.5, j_pulse=-0.125, pKa6=7, d_film=1.6, cAmine_tot=0, Film_Phosphate_Scale=1, Film_H_OH_Scale=1, k_b1=5E10, Cdl=20 (su1)

GENERAL

Description	Value
Solution	k_app1=0.01, pH_b=4.5, j_pulse= - 0.125, pKa6=7, d_film=1.6, cAmine_tot=0, Film_Phosphate_Scale=1, Film_H_OH_Scale=1, k_b1=5E10, Cdl=20 (sol54)

k_app1=0.01, pH_b=4.5, j_pulse=-0.125, pKa6=7, d_film=1.6, cAmine_tot=3.8, Film_Phosphate_Scale=1, Film_H_OH_Scale=1, k_b1=5E10, Cdl=20 (su2)

GENERAL

Description	Value
Solution	k_app1=0.01, pH_b=4.5, j_pulse= - 0.125, pKa6=7, d_film=1.6, cAmine_tot=3.8, Film_Phosphate_Scale=1, Film_H_OH_Scale=1, k_b1=5E10, Cdl=20 (sol55)

k_app1=0.01, pH_b=4.5, j_pulse=-0.25, pKa6=7, d_film=1.6, cAmine_tot=0, Film_Phosphate_Scale=1, Film_H_OH_Scale=1, k_b1=5E10, Cdl=20 (su3)

GENERAL

Description	Value
Solution	k_app1=0.01, pH_b=4.5, j_pulse= - 0.25, pKa6=7, d_film=1.6, cAmine_tot=0, Film_Phosphate_Scale=1, Film_H_OH_Scale=1, k_b1=5E10, Cdl=20 (sol56)

k_app1=0.01, pH_b=4.5, j_pulse=-0.25, pKa6=7, d_film=1.6, cAmine_tot=3.8, Film_Phosphate_Scale=1, Film_H_OH_Scale=1, k_b1=5E10, Cdl=20 (su4)

GENERAL

Description	Value
Solution	k_app1=0.01, pH_b=4.5, j_pulse= - 0.25, pKa6=7, d_film=1.6, cAmine_tot=3.8, Film_Phosphate_Scale=1, Film_H_OH_Scale=1, k_b1=5E10, Cdl=20 (sol57)

k_app1=0.01, pH_b=4.5, j_pulse=-0.5, pKa6=7, d_film=1.6, cAmine_tot=0, Film_Phosphate_Scale=1, Film_H_OH_Scale=1, k_b1=5E10, Cdl=20 (su5)

GENERAL

Description	Value
Solution	k_app1=0.01, pH_b=4.5, j_pulse= - 0.5, pKa6=7, d_film=1.6, cAmine_tot=0, Film_Phosphate_Scale=1, Film_H_OH_Scale=1, k_b1=5E10, Cdl=20 (sol58)

k_app1=0.01, pH_b=4.5, j_pulse=-0.5, pKa6=7, d_film=1.6, cAmine_tot=3.8, Film_Phosphate_Scale=1, Film_H_OH_Scale=1, k_b1=5E10, Cdl=20 (su6)

GENERAL

Description	Value
Solution	k_app1=0.01, pH_b=4.5, j_pulse= - 0.5, pKa6=7, d_film=1.6, cAmine_tot=3.8, Film_Phosphate_Scale=1, Film_H_OH_Scale=1, k_b1=5E10, Cdl=20 (sol59)

6 Results

6.1 PLOT GROUPS

6.1.1 Study 1 - LSV

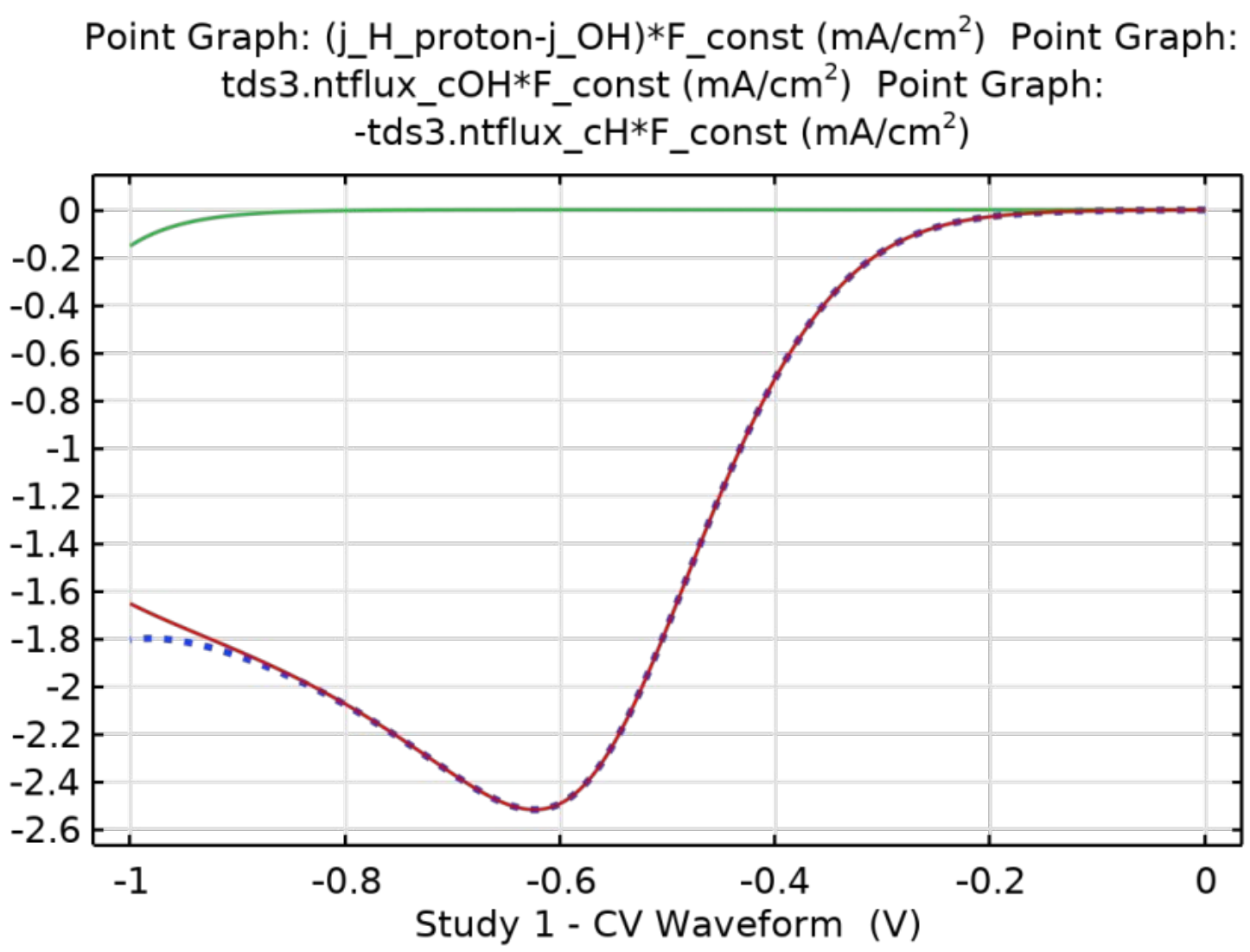

*Point Graph: (j_H_proton-j_OH)*F_const (mA/cm²) Point Graph: tds3.ntflux_cOH*F_const (mA/cm²) Point Graph: -tds3.ntflux_cH*F_const (mA/cm²)*

6.1.2 Study 3 - CP - pH in film - 120 s

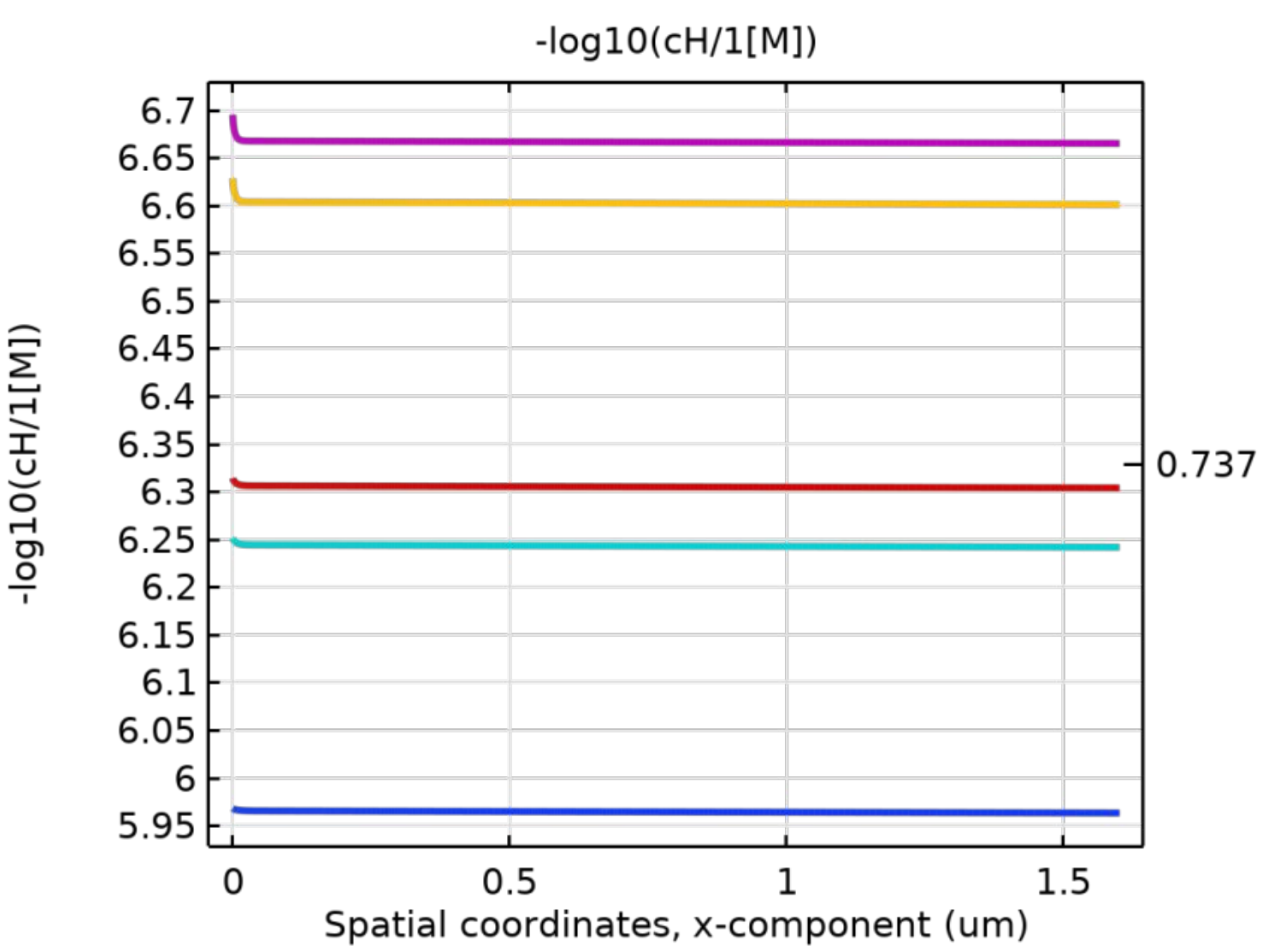

-log10(cH/1[M])

6.1.3 Study 3 - CP - Voltage vs Time

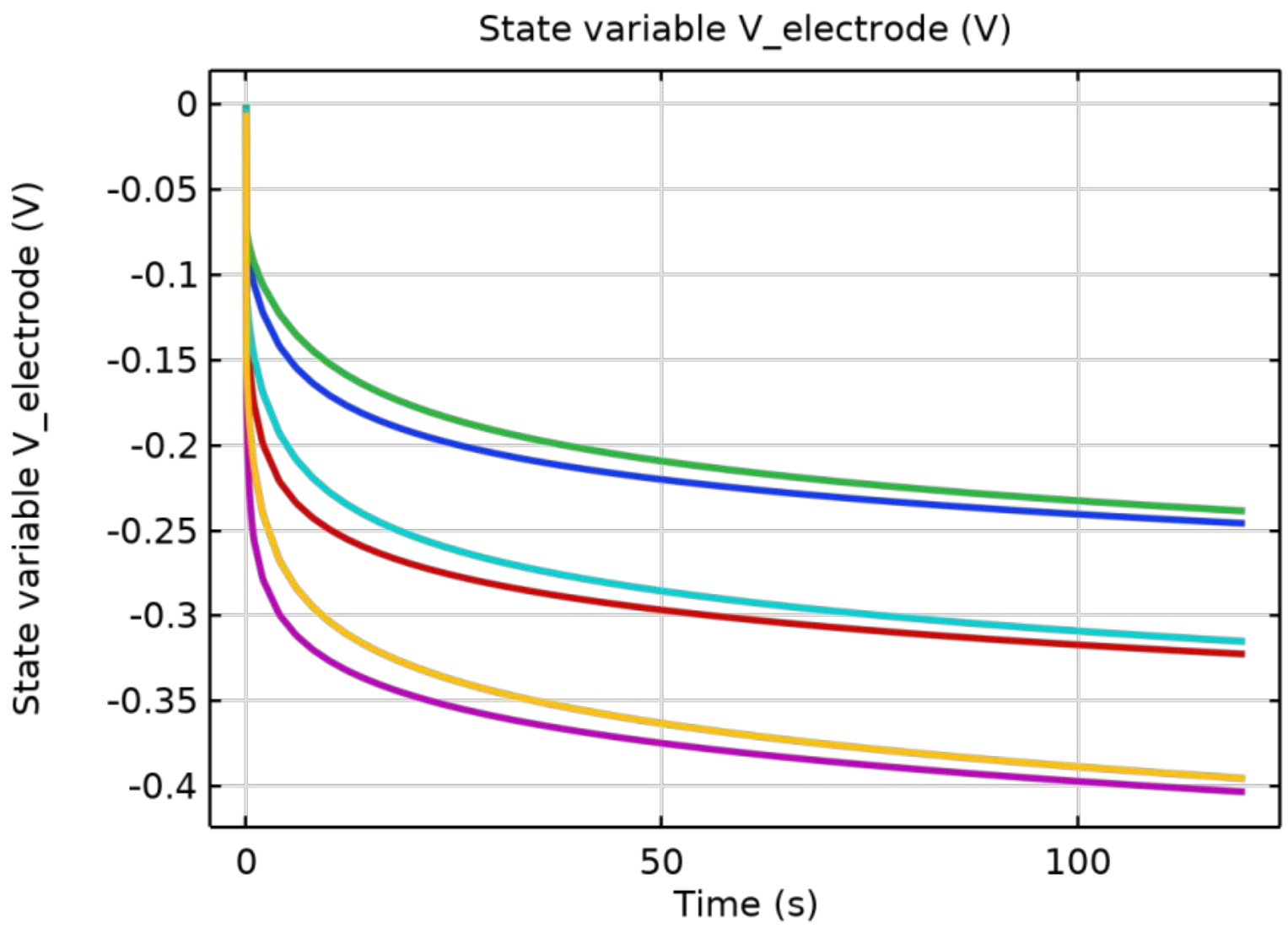

State variable V_electrode (V)

6.1.4 Study 3- CP - Average film pH

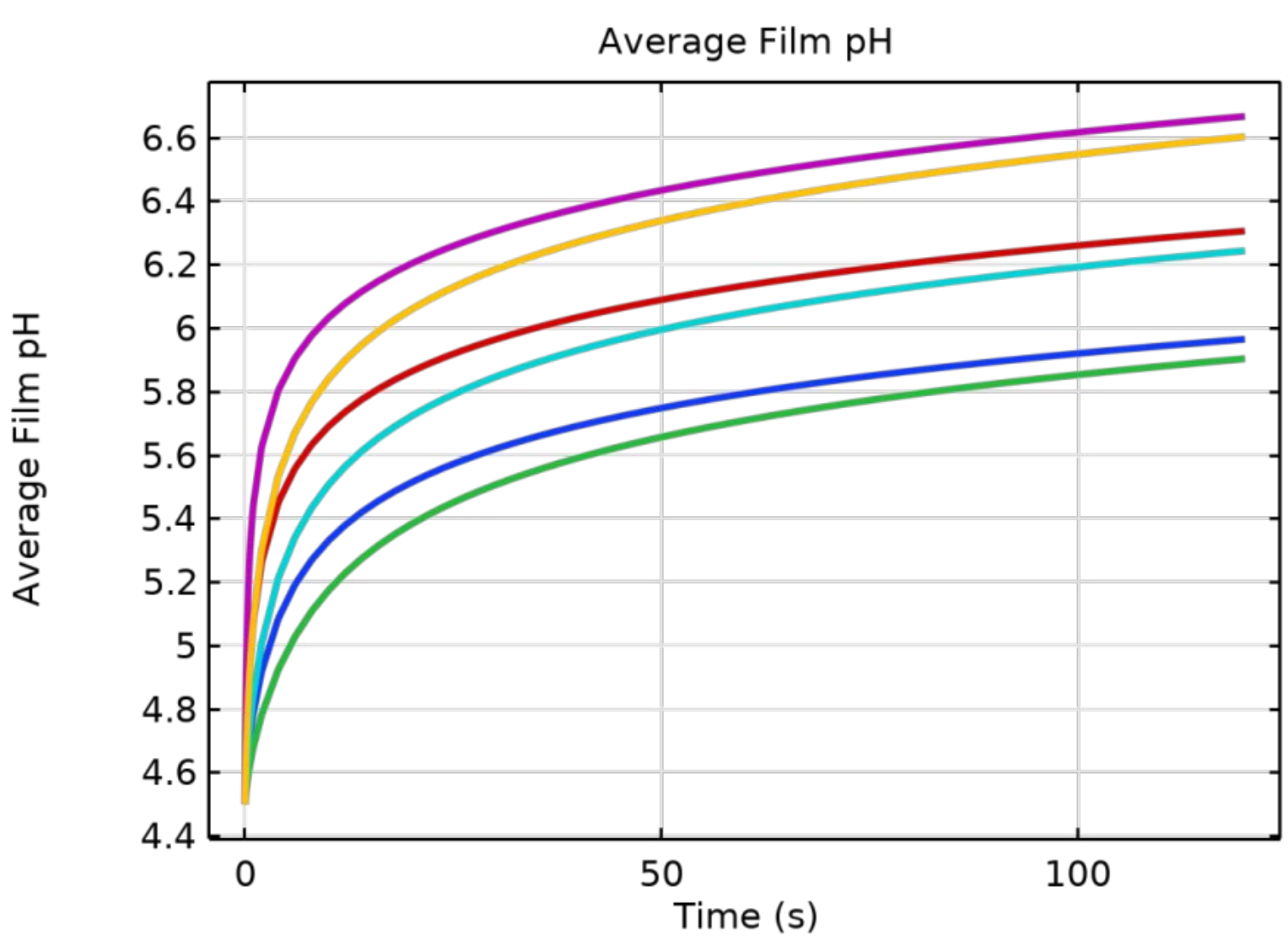

Average Film pH

6.1.5 Study 3 - CP - pH in film - Plot During Simulation

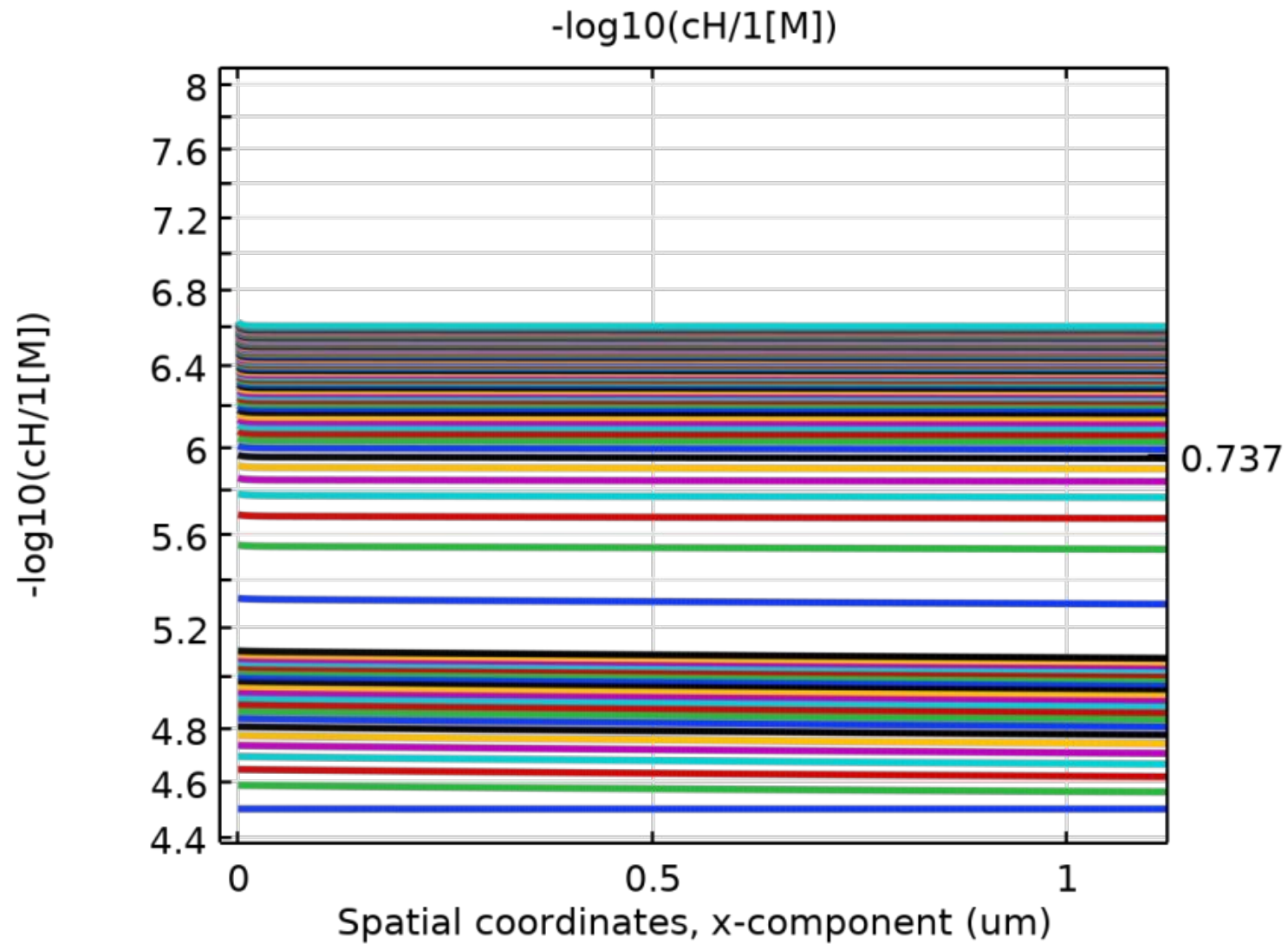

-log10(cH/1[M])

6.1.6 Probe Plot Group 52

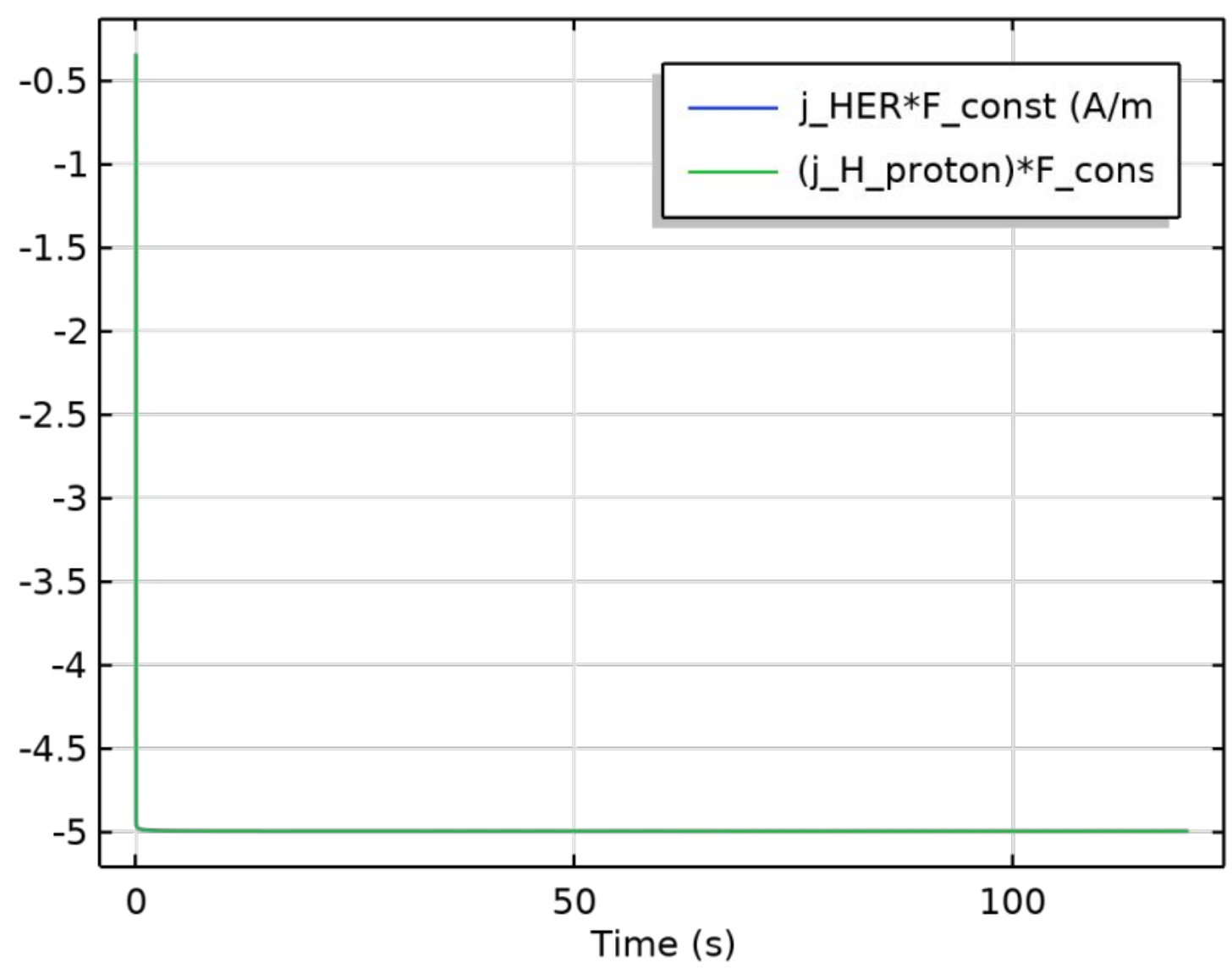

6.1.7 Probe Plot Group 53

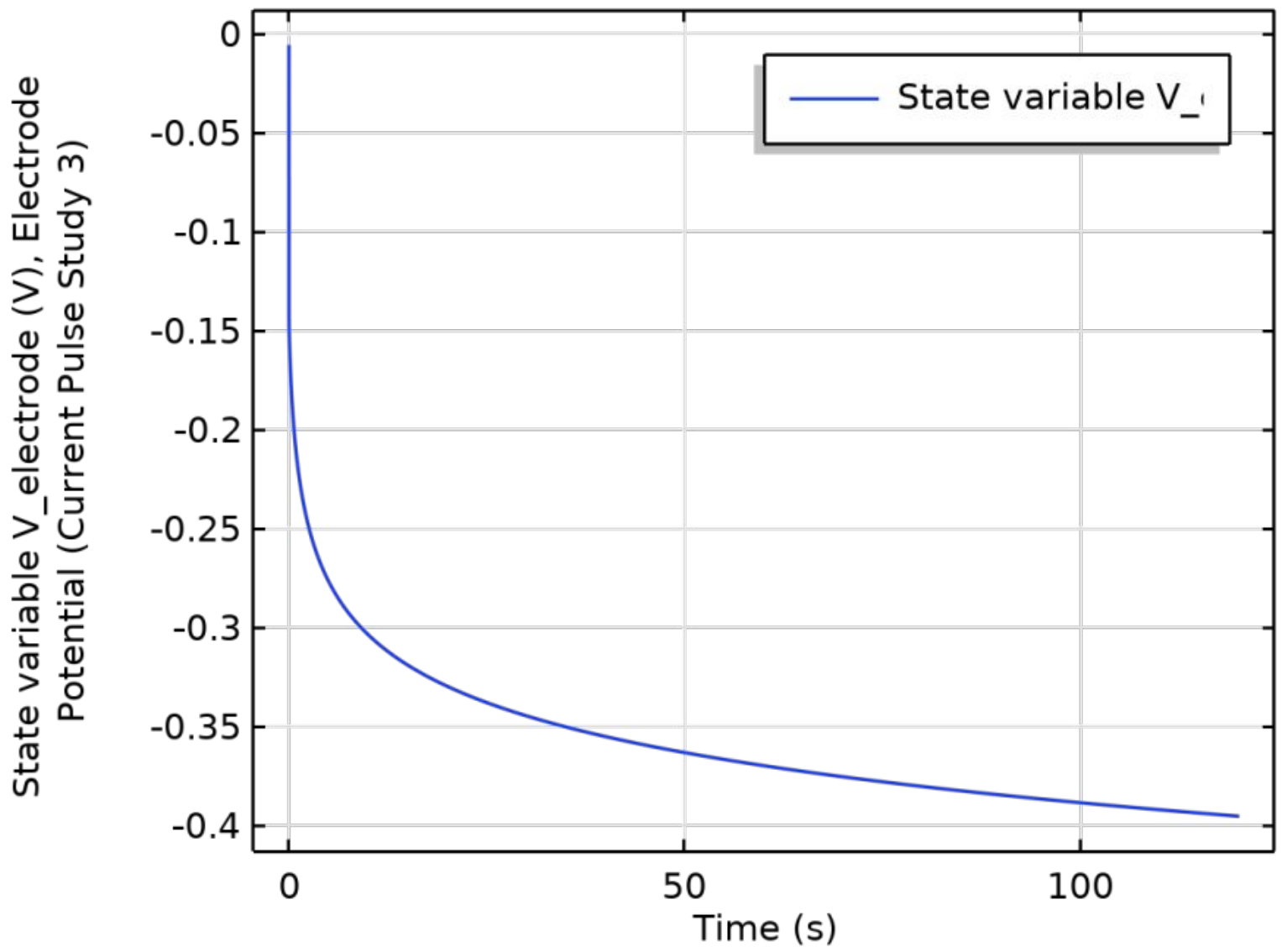

Supplement: Supplementary file 2 [file ja6c11297_si_002.pdf]
